# Reversible RNA ADP-ribosylation on uracil bases

**DOI:** 10.1093/nar/gkag289

**Published:** 2026-03-31

**Authors:** Yang Lu, Li Tang, Øyvind Strømland, Chatrin Chatrin, Kang Zhu, Deeksha Munnur, Joséphine Groslambert, Petra Mikolčević, Herwig Schüler, Gyula Timinszky, Guillaume Gabant, Marcin J Suskiewicz, Andreja Mikoč, Vincent Aucagne, Dragana Ahel, Qiang Liu, Ivan Ahel

**Affiliations:** Sir William Dunn School of Pathology, University of Oxford, OxfordOX1 3RE, United Kingdom; Carbohydrate-Based Drug Research Centre, Shanghai Institute of Materia Medica, Chinese Academy of Sciences, Shanghai 201203, China; Zhongshan Institute for Drug Discovery, Chinese Academy of Sciences, Zhongshan 528400, China; School of Pharmacy, University of the Chinese Academy of Sciences, Beijing 100049, China; Department of Biomedicine, University of Bergen, Bergen5020, Norway; Sir William Dunn School of Pathology, University of Oxford, OxfordOX1 3RE, United Kingdom; Sir William Dunn School of Pathology, University of Oxford, OxfordOX1 3RE, United Kingdom; Health Science Centre, East China Normal University, Shanghai 200241, China; Sir William Dunn School of Pathology, University of Oxford, OxfordOX1 3RE, United Kingdom; Sir William Dunn School of Pathology, University of Oxford, OxfordOX1 3RE, United Kingdom; Division of Molecular Biology, Ruđer Bošković Institute, Zagreb 10000, Croatia; Division of Biochemistry and Structural Biology, Department of Chemistry, Lund University, LundSE-22362, Sweden; Laboratory of DNA Damage and Nuclear Dynamics, Institute of Genetics, HUN-REN Biological Research Centre, Szeged 6726, Hungary; Centre de Biophysique Moléculaire, CNRS UPR 4301, affiliated with Université d’Orléans, Orléans45071,France; Centre de Biophysique Moléculaire, CNRS UPR 4301, affiliated with Université d’Orléans, Orléans45071,France; Division of Molecular Biology, Ruđer Bošković Institute, Zagreb 10000, Croatia; Centre de Biophysique Moléculaire, CNRS UPR 4301, affiliated with Université d’Orléans, Orléans45071,France; Sir William Dunn School of Pathology, University of Oxford, OxfordOX1 3RE, United Kingdom; Carbohydrate-Based Drug Research Centre, Shanghai Institute of Materia Medica, Chinese Academy of Sciences, Shanghai 201203, China; Zhongshan Institute for Drug Discovery, Chinese Academy of Sciences, Zhongshan 528400, China; School of Pharmacy, University of the Chinese Academy of Sciences, Beijing 100049, China; Sir William Dunn School of Pathology, University of Oxford, OxfordOX1 3RE, United Kingdom

## Abstract

ADP-ribosylation is a conserved modification that uses NAD^+^ as a co-substrate to regulate essential cellular processes, such as genome stability and transcription, with Poly(ADP-ribose) Polymerases (PARPs) serving as the major catalyzing enzymes in humans. Historically defined as a protein post-translational modification, ADP-ribosylation on nucleic acids has been increasingly recognized in recent years, particularly in bacterial systems, but remains poorly understood in higher organisms. Here, we identify human PARP10 as a candidate enzyme that ADP-ribosylates nucleic acid bases, showing apparent activity on uracil bases in RNA, and a relatively weaker activity toward thymine bases in DNA. Furthermore, we show that human TARG1, a neurodegenerative disorder-linked protein previously reported to hydrolyse thymine base ADP-ribosylation, also efficiently reverses uracil base ADP-ribosylation (U-ADPr). To improve the efficient characterization of the enzymes for U-ADPr reversal, we developed chemical probes. Using these probes, we demonstrated that human TARG1 and TARG1-like macrodomain proteins are the efficient hydrolases for U-ADPr reversal in humans, *Drosophila melanogaster*, and bacterial homologues. The widespread distribution of U-ADPr hydrolases among different organisms suggests the potential evolutionary conservation of U-ADPr as a biological signal.

## Graphical Abstract

**Graphical Abstract:**
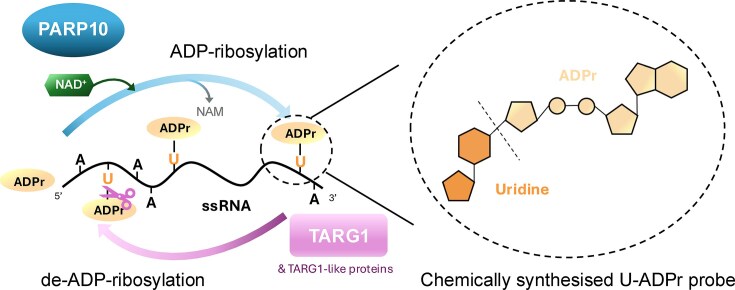

## Introduction

ADP-ribosylation is an evolutionarily conserved macromolecular modification that regulates many essential cellular processes, including DNA damage repair, transcription, metabolism, high-order organismal behaviours, and more [1–3]. This modification is catalyzed by a variety of ADP-ribosyltransferases (ARTs) that use nicotinamide adenine dinucleotide (NAD^+^) as a co-substrate, among which the PARPs constitute the largest family in humans [4]. ADP-ribosylation is reversible, and at least three evolutionary distinct enzyme families, including macrodomains, (ADP-ribosyl)hydrolases (ARHs), and NADARs (‘NAD- and ADP-ribose’-associated enzymes), have been shown to hydrolase and fine-tune ADP-ribosylation signalling [5, 6].

Over the past decades, ADP-ribosylation has been characterized predominantly as a post-translational modification of proteins, especially in the context of the DNA damage response and chromatin regulation in higher organisms [7–9], and more recently in antiviral immunity [10]. For example, PARP1, PARP2, and PARP3 function directly in DNA damage repair in humans [11–13], whereas PARP10 has been characterized as both an antiviral enzyme and a key regulator of DNA replication stress responses [14, 15]. More recently, emerging evidence has shown that nucleic acids, including DNA and RNA, can also serve as targets for ADP-ribosylation, a modification that appears to be widespread, at least in bacterial systems [16]. More specifically, reversible DNA ADP-ribosylation has been characterized on specific bases, most commonly thymine and guanine, and is regulated by bacterial toxin–antitoxin systems that control DNA replication, transcription, and anti-phage response [6, 17–20]. To date, RNA-base ADP-ribosylation has been described as irreversible, as illustrated by adenine modification catalysed by CmdT toxins as a part of microbial warfare mechanism [19]. Reversible RNA ADP-ribosylation has been only described on the 2′-hydroxyl group (2′-OH) of ribose in double-stranded RNA (dsRNA), where it is catalysed by RhsP2 toxin from *Pseudomonas aeruginosa*, and can be reversed by PARGs from the macrodomain hydrolase family [21, 22]. In higher organisms, particularly in humans, nucleic acid ADP-ribosylation remains poorly understood. Reversible ADP-ribosylation on phosphate groups at the ends of DNA or RNA has been reported in *in vitro* or overexpression systems [23–25]. ADP-ribosylation has also been observed on human telomeres, although the specific linkage remains unclear [26]. By contrast, base modifications have so far been demonstrated only on adenine bases in DNA, with their physiological roles still under investigation [27], and there are currently no reports of RNA base modifications in humans [28].

In this study, we analysed the catalytic activity of several recombinant human PARP ARTs and identified PARP10 as exhibiting notable activity in modifying uracil bases in single-stranded RNA (ssRNA), with lower activity toward thymine bases in single-stranded DNA (ssDNA). We further demonstrate that human TARG1, a macrodomain-containing hydrolase with a key role in neurodegenerative disorders and previously defined hydrolytic activity in thymine-ADPr (T-ADPr) reversal [29], also efficiently reverses uracil ADP-ribosylation (U-ADPr) catalysed by PARP10. Similarly, bacterial hydrolases, including *Thermus aquaticus* DarG and its homologous protein SCO6735 from *Streptomyces coelicolor* [30], are also catalytically active in reversing U-ADPr. To facilitate these studies, we developed chemical probes that enabled efficient characterization of different hydrolases acting on U-ADPr, confirming human TARG1 and TARG1-like macrodomain proteins as the major hydrolases responsible for reversing U-ADPr in humans, *Drosophila melanogaster*, and bacteria. Together, the presence of hydrolases capable of reversing U-ADPr suggests that this modification may be both widespread and evolutionarily conserved across diverse organisms.

## Materials and methods

### Materials

Cy3-labelled ssDNA and ssRNA oligonucleotides used in this study were purchased from Integrated DNA Technologies and Thermo Scientific. For annealing the double-stranded oligonucleotides, the two complementary oligonucleotide strands were mixed at equimolar concentrations in an annealing buffer containing 10 mM Tris–HCl (pH 7.5), 50 mM NaCl, and 1 mM ethylenediaminetetraacetic acid (EDTA). The mixture was incubated at 95°C for 5 min, then gradually cooled to room temperature for 2 h. Successful annealing was verified using a native 20% acrylamide gel. The specific sequences of the oligonucleotides are indicated below each gel in the Results section and listed in Supplementary Table S1.

### Recombinant protein expression and purification

To assemble the human PARP10 protein expression construct, complementary DNAs encoding the wild-type and G888W catalytic-mutant ART-domain fragment (PARP10_809–1017_), codon-optimized for *Escherichia coli* expression, were obtained from GeneArt (Thermo Fisher Scientific) and cloned into the pNIC28-Bsa4 expression vector (GenBank: EF198106.1) using ligase-independent cloning. This approach introduced a 22-residue N-terminal extension comprising a hexahistidine tag, followed by a Tobacco etch virus protease cleavage site.

To purify recombinant PARP10 (WT and G888W mutant) from the expression constructs described above, *E. coli* Rosetta (BL21 DE3) cells were transformed with respective constructs and grown in Lysogeny broth medium (Sigma) at 37°C supplemented with 50 µg/ml kanamycin and 35 µg/ml chloramphenicol. After culture reached an OD_600_ of 0.5, the temperature was reduced to 18°C, and protein expression was induced overnight with 0.5 mM Isopropyl β-D-1-thiogalactopyranoside (IPTG). Cells were then harvested and resuspended in lysis buffer containing 25 mM Tris–HCl (pH 7.5), 500 mM NaCl, 20 mM imidazole, 5 mM β-mercaptoethanol, cOmplete™ EDTA-free protease inhibitor (Roche), 2.5 U/ml benzonase (Merck Life Science), and 5 mM MgCl_2_, followed by high-pressure homogenization. Recombinant PARP10s were purified using ÄKTA fast protein liquid chromatography system (GE Healthcare Life Sciences) through a three-step procedure. First, His-tagged proteins were captured from the bacterial lysate on a HisTrap HP column (Cytiva), equilibrated and washed with buffer containing 25 mM Tris–HCl (pH 7.5), 500 mM NaCl, 20 mM imidazole, and 5 mM β-mercaptoethanol, and eluted with the same buffer in which imidazole was increased to 200 mM. Second, the eluted proteins were diluted in a no-salt buffer containing 25 mM Tris–HCl (pH 7.5) and 1 mM Dithiothreitol (DTT) to a final NaCl concentration of 50 mM, loaded to a HiTrap Heparin HP column (Cytiva) equilibrated with the same buffer, and eluted with a linear gradient to 1000 mM NaCl, eliminating nucleic acid contaminants. Third, fractions were analysed by sodium dodecyl sulphate–polyacrylamide gel electrophoresis (SDS-PAGE), and those containing the target protein were collected and further purified by size-exclusion chromatography on a HiLoad 16/600 Superdex 200 pg column (Cytiva) using buffer containing 25 mM Tris–HCl (pH 7.5), 150 mM NaCl, and 1 mM DTT for molecular-size separation. Fractions containing the target protein were collected, concentrated, flash-frozen in liquid nitrogen, and stored at –80°C.

**Figure 1.**
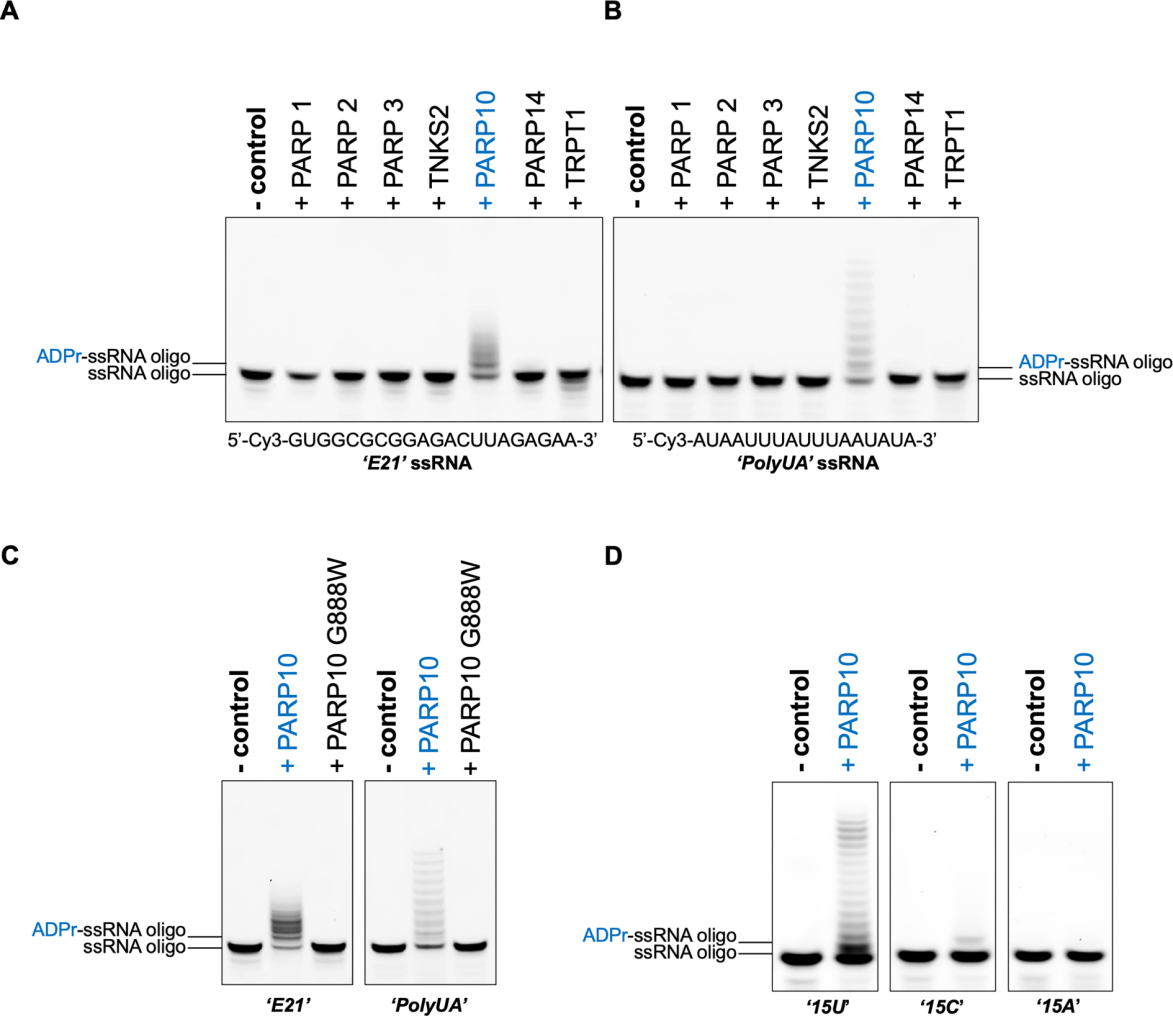
PARP10 preferentially catalyses ADP-ribosylation of RNA at the uracil base. (A, B) Among selected human PARP-family members and the PARP-like protein TRPT1, PARP10 is the only transferase that exhibits catalytic activity towards a ssRNA oligonucleotide, as determined by *in vitro* gel-shift ADP-ribosylation assays. Two ssRNA oligonucleotides were used for comparison: ‘*E21*’, a random sequence (**A**), and ‘*PolyUA*’, a UA-rich sequence (**B**), with the specific sequences indicated below the corresponding gel. (**C**) Wild-type PARP10 ADP-ribosylates both ‘*E21*’ and ‘*PolyUA*’ oligonucleotides, in contrast to its catalytic mutant G888W. (**D**) Chemically synthesized 15-nt ssRNA oligonucleotides with different bases (U, C, and A) were used to assess the sequence specificity of PARP10 (oligonucleotide sequences indicated in Supplementary Table S1). For all figures, in the ‘- control’, the enzyme was replaced with an equal volume of reaction buffer, while all other reaction conditions were kept identical.

For the PARPs shown in Fig. 1A and B, PARP1, PARP2, PARP3, and TRPT1 were constructed and expressed as full-length proteins, TNKS2 (PARP5B) contained sterile alpha motif and catalytic domain, and PARP14 included the KH8 and WWE domains along with the catalytic domain (1453–1801); constructs and purification protocols were performed as previously described [13, 24, 31–33]. Furthermore, for the human, *D. melanogaster*, and bacterial hydrolases, constructs and purification were carried out as described previously [22, 34–37].

*Streptomyces coelicolor* ARH-like hydrolases were cloned into pET28b and expressed in *E. coli* BL21(DE3) cells. Cultures were grown at 37°C in Terrific Broth medium (Difco) supplemented with 35 µg/ml kanamycin. At an OD_600_ of 0.8, expression of SCO0086, SCO2030, and SCO5809 was induced with 0.8 mM IPTG and incubation continued at 30°C for 3 h. Cells were harvested by centrifugation and resuspended in buffer containing 25 mM Tris–HCl (pH 7.5), 500 mM NaCl, and 10 mM imidazole, then lysed using lysozyme (1 mg/ml) and sonication. Cell debris was removed by centrifugation, and the His-tagged recombinant proteins were purified using TALON^®^ metal affinity chromatography (Clontech). The resin was washed with the same buffer containing increasing concentrations of imidazole (10, 20, and 40 mM), and the proteins were eluted with buffer containing 200 mM imidazole. Purified fractions were pooled, desalted and stored in buffer containing 25 mM Tris–HCl (pH 7.5), 50 mM NaCl, 1 mM EDTA, 1 mM DTT, and 10% glycerol at −80°C. The same protocol was used for SCO2028 except induction was performed with 0.1 mM IPTG and continued overnight at 16°C.

### Gel-based ADP-ribosylation activity assays

*In vitro* oligonucleotide ADP-ribosylation assays were performed in a buffer containing 20 mM HEPES-KOH (pH 7.6), 50 mM KCl, 5 mM MgCl_2_, and 1 mM DTT. For ADP-ribosylation assays, reactions (10 µl total volume) contained 1 µM ARTs, 0.3 µM or 1 µM Cy3-labelled ss/dsDNA or ss/dsRNA oligonucleotides, and 1 mM β-NAD⁺, and were incubated at 37°C for 60 min. For negative controls (shown as the ‘− control’ in figures), buffer was added in place of ARTs, serving as an internal reference for unmodified oligonucleotides. Reactions were terminated by heating at 95°C for 5 min and then cooled on ice. For de-ADP-ribosylation assays (10 µl final volume), ADP-ribosylated oligonucleotide samples were incubated with either reaction buffer (‘+ control’) or with 1 µM of the indicated hydrolase (except in Fig. 3E, left panel, where hydrolases were used at 0.04 µM) at 37°C for 30 min. Reactions were terminated by adding 10 µl urea loading dye [10 mM Tris–HCl (pH 8.0), 10 mM EDTA, 4 M urea], heating at 95°C for 5 min, and then cooling on ice before loading onto the gel for analysis. Final reaction products were analysed on urea-denaturing polyacrylamide gels by separating them in 1× Tris/Borate/EDTA buffer. The gel was analysed and imaged using the Molecular Imager PharosFX system (Bio-Rad) with 532 nm laser excitation to visualize Cy3-labelled oligonucleotides.

For all Results panels, representative results from two independent experiments are presented.

### MALDI-TOF mass spectrometry analyses

*In vitro* ADP-ribosylation reaction mixtures were desalted using ZipTip C18 (Millipore) prior to MALDI-TOF analysis. The matrix solution consisted of 3-hydroxy-picolinic acid prepared at 10 mg/ml in a solution of 10% acetonitrile and 1 mg/ml di-ammonium hydrogen citrate. Desalted sample and matrix solution were mixed at a 0.5:1 ratio directly on the AnchorChip^TM^ target and allowed to air-dry. MALDI-TOF-MS spectra were acquired on an UltrafleXtreme mass spectrometer (Bruker Daltonics) in the linear positive ion mode over an *m*/*z* range of 1600–20 000. External calibration was performed using a neighboring spot containing an oligonucleotide calibration standard (Bruker Daltonics). Spectra were processed using FlexAnalysis 3.4 software (Bruker Daltonics).

### HPLC-MS analyses

HPLC-MS analyses were carried out on an Agilent 1260 Infinity HPLC system, coupled with an Agilent 6120 mass spectrometer [electrospray ionization + mode] and fitted with a Chromolith® HighResolution RP-18 endcapped (monolithic) column (100 mm × 3 mm). As mobile phases, mixtures of 0.1% formic acid in H_2_O (A) and 0.1% formic acid in MeCN (B) were used. Gradient: 0% B for 5 min, then 0 to 90% B over 6 min, 0.6 ml/min flow rate.

### Electrophoretic mobility shift assay

Replication Protein A and PARP10, at concentrations of 1.5, 4.0, or 10.0 µM, were incubated with 0.2 µM Cy3-labelled ‘*E21*’ ssDNA or ssRNA in buffer containing 50 mM Tris–HCl (pH 8.0), 30 mM NaCl, 5 mM EDTA, 1 mM DTT, 100 µg/ml bovine serum albumin, and 5% glycerol. Each reaction, with a total volume of 10 µl, was incubated at room temperature for 10 min before loading onto a 6% native PAGE gel. The gel was analysed and imaged using the Molecular Imager PharosFX system (Bio-Rad) with 532 nm laser excitation to visualize Cy3-labelled oligonucleotides.

### Material and general procedures for chemical synthesis

Commercial reagents were used without further purification unless noted. Reactions were carried out under standard anhydrous conditions and monitored by TLC. Products were purified by silica gel or size-exclusion chromatography as appropriate. NMR spectra were recorded in common deuterated solvents, and HRMS analyses were performed using an Orbitrap instrument. Full experimental procedures and complete characterization data for all new compounds are provided in the Supplementary Information. ^1^H, ^13^C NMR, and 2D NMR spectra of all new compounds are shown in Supplementary Information (Chemical Synthesis Dataset).

### Hydrolase activity analysis using luminescence detection of ADP-ribose via AMP-Glo assays

The ADP-ribosylated uridine nucleotides (both α- and β-anomers) used in this study were synthesized as described in the Results section. The AMP-Glo assay was used to analyse the hydrolytic activity of selected enzymes in this study, following the method previously established for peptide-ADPr substrates [38]. In brief, assays were performed with 10 µM nucleotide substrate and 500 nM hydrolase in buffer containing 50 mM Tris-HCl [pH 7.5], 200 mM NaCl, 10 mM MgCl_2_, 1 mM DTT, and 0.2 µM Nudix hydrolase 5 (NUDT5) for 30 min at 30 °C. Reactions were analysed using the AMP-Glo™ assay kit (Promega) following the instructions provided by the manufacturer, with luminescence measured and recorded on a SpectraMax M5 plate reader with SoftMax Pro software (Molecular Devices). Final data were analysed using GraphPad Prism. All measurements were background-corrected relative to the luminescence signal obtained in the presence of NUDT5 but in the absence of hydrolases, which serves as the baseline control for adenosine monophosphate (AMP) generated from substrate processing by NUDT5. Data are presented as the mean ± standard deviation of three independent replicates.

## Results

### Human PARP10 catalyses RNA ADP-ribosylation at uracil bases

Human PARP family members have so far been shown to modify RNA substrates only at terminal phosphate groups [24]. To determine whether any human PARPs can directly ADP-ribosylate RNA bases, we selected a panel of PARPs representing multiple evolutionary clades and functional groups, as well as the TRPT1 PARP-like protein, to capture the breadth of ART activities [3, 39]. We expressed and purified recombinant PARP enzymes and TRPT1 (full-length proteins, shorter truncations, or catalytic fragments, as defined in ‘Materials and methods’ section) and assessed their activity on a 21-nt ssRNA oligonucleotide of randomized sequence (‘*E21*’) in the presence of NAD^+^, the PARP co-substrate for ADP-ribosylation (Fig. 1A). Strikingly, the PARP10 catalytic domain (hereafter referred to as PARP10) exhibited robust ADP-ribosylation, detected as a ladder-like mobility shift, indicative of multiple modification sites within the ssRNA oligonucleotide. No detectable activity was observed for other human PARPs tested (Fig. 1A), including DNA repair-associated PARPs 1–3, TNKS2 (PARP5B), PARP14, and the PARP-like protein TRPT1, most of which have been reported to act on DNA or RNA substrates with phosphorylated ends [23, 24, 40, 41]. We next tested an adenine- and uracil- rich (AU-rich) 17-nt ssRNA oligonucleotide (‘*PolyUA*’), designed based on naturally occurring tissue factor AU-rich element (ARE) sequences [42]. Interestingly, PARP10 catalysis produced an even more pronounced laddering effect, suggesting that adenine or uracil bases may serve as primary modification targets (Fig. 1B). Consistent with the previous observation on ‘*E21*’ ssRNA (Fig. 1A), none of the other PARPs exhibited detectable activity (Fig. 1B). The observed U-ADPr modifications depended on the catalytic activities of the ART domain of PARP10, as modifications were completely abolished in its catalytically inactive G888W mutant (Fig. 1C). Next, to identify the preferred base for PARP10-catalysed RNA modification, we tested PARP10 with NAD^+^ on synthesized 15-nt ssRNA oligonucleotides composed of different bases, including 15 consecutive uracil (‘*15U*’), cytosine (‘*15C*’), or adenine (‘*15A*’) bases. A 15-nt guanine ssRNA oligonucleotide could not be synthesized due to technical limitations. The results unambiguously demonstrate that PARP10 preferentially targets uracil-containing RNA (Fig. 1D), with a minor modification of cytosine-containing RNA (Fig. 1D) possibly reflecting spontaneous cytosine-to-uracil deamination, a well-documented phenomenon [43], followed by ADP-ribosylation by PARP10. We next assessed whether terminal phosphorylation influences PARP10-catalysed uracil ADP-ribosylation using ‘*15U*’ ssRNA oligonucleotides differing in the presence and position of 5′ or 3′ phosphates (Supplementary Table S1 and Supplementary Fig. S1). We found that phosphorylation had no substantial effect on PARP10-catalysed uracil ADP-ribosylation, although an additional mobility shift was observed, possibly reflecting modification of the terminal phosphate (Supplementary Fig. S1), consistent with previous discoveries [24].

**Figure 2.**
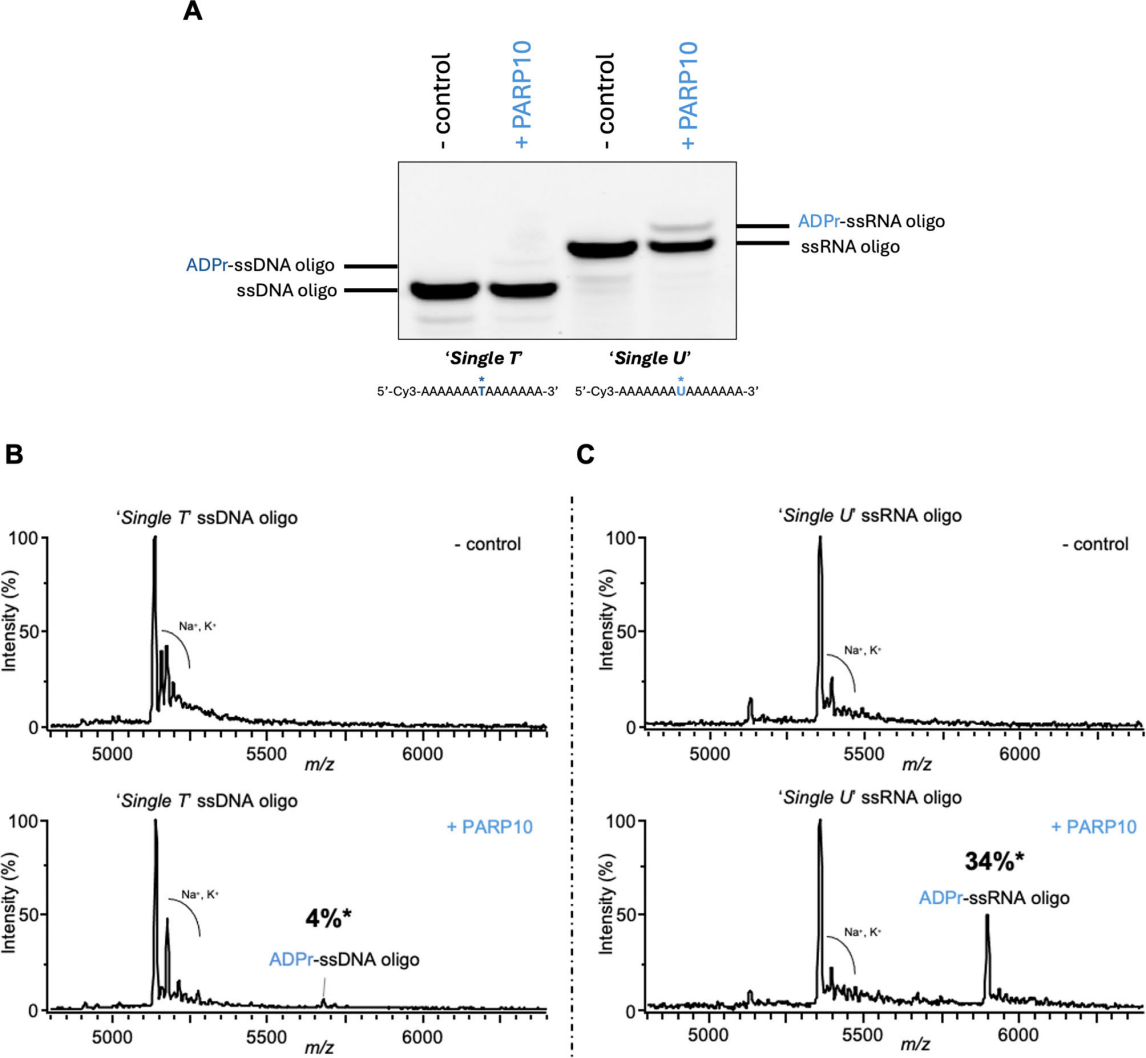
PARP10 shows higher ADP-ribosylation activity on RNA (uracil) compared to DNA (thymine). (**A**) *In vitro* ADP-ribosylation assays comparing the PARP10 catalytic activity on ssDNA and ssRNA oligonucleotides containing a single thymine (left; *'Single T'*) or uracil (right; *'Single U'*), respectively. The oligonucleotide sequences are shown below the gel, with the modification site marked by an asterisk. MALDI-TOF-MS mass spectra of incubation of *‘Single-T’* ssDNA (**B**) and *‘Single-U’* ssRNA (**C**) oligonucleotides with NAD^+^ in the absence and presence of PARP10. The mass-to-charge (*m*/*z*) of individual molecule species is shown on the *x*-axis, and intensity on the *y*-axis. The expected ADP-ribosylation mass shift (+541 Da) is observed predominantly for RNA compared to DNA oligonucleotides. *Assuming comparable ionization efficiencies for modified and unmodified species, the extent of modification is estimated to be ~4% for DNA and 34% for RNA.

**Figure 3.**
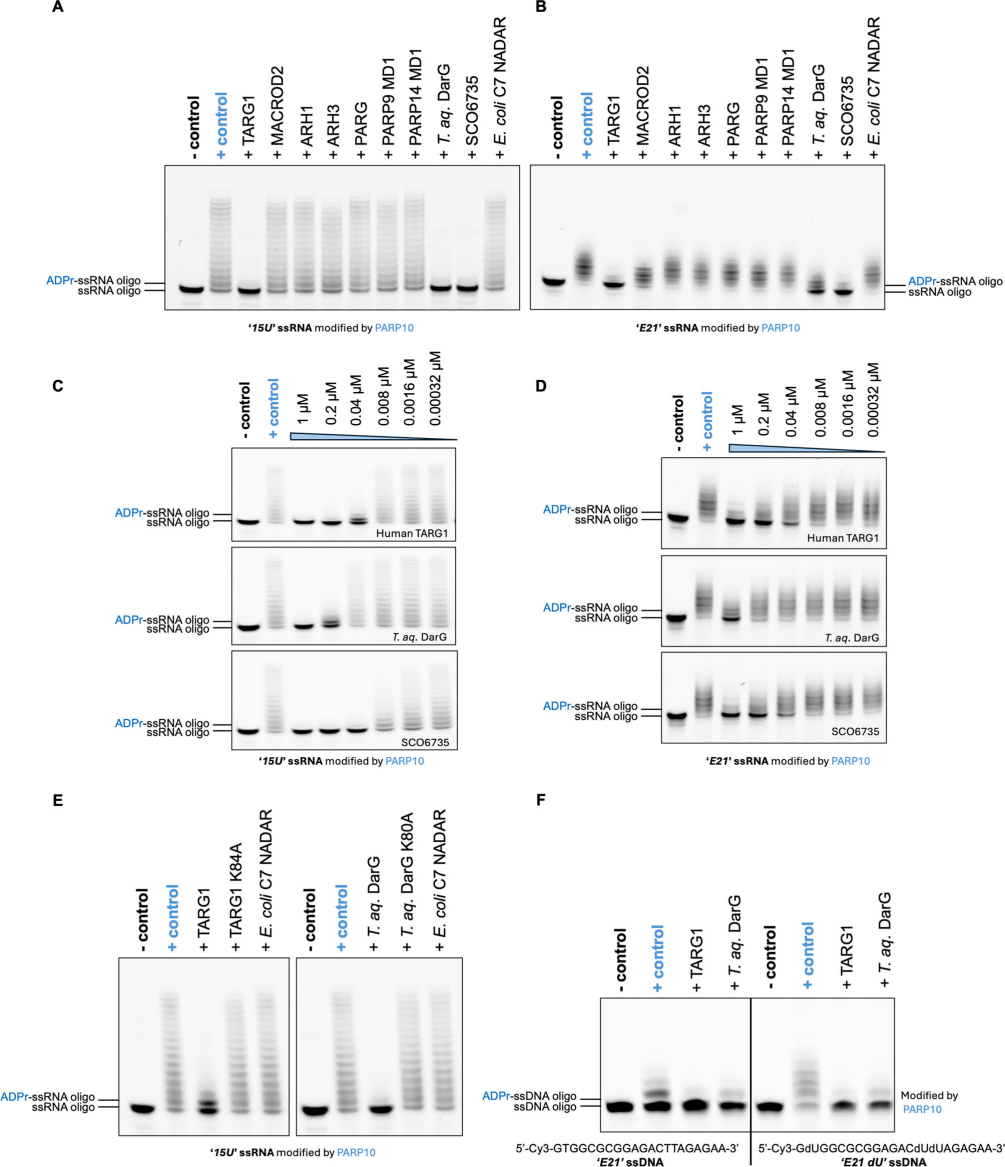
Reversal of PARP10-catalysed ssRNA ADP-ribosylation by human TARG1 and TARG1-like bacterial macrodomain ADP-ribosylhydrolases. *In vitro* de-ADP-ribosylation assays show that only human macrodomain hydrolase TARG1 and the bacterial macrodomain hydrolases *T. aquaticus* (*T. aq*. ) DarG and SCO6735 notably reversed PARP10-modified ssRNA oligonucleotides ‘*15U*’ (**A**) and ‘*E21*’ (**B**), whereas other human macrodomain-, ARH-family hydrolases, and bacterial NADAR-family hydrolases displayed no or negligible activity. Comparison of the hydrolytic efficiencies of human TARG1, *T. aquaticus* DarG, and SCO6735 on PARP10-modified ssRNA oligonucleotides ‘*15U*’ (**C**) and ‘*E21*’ (**D**). (**E**) Wild-type human TARG1 (left) and *T. aquaticus* DarG (right) reverse PARP10-catalysed ADP-ribosylation on uracil bases within the ‘*15U*’ ssRNA oligonucleotides compared to their catalytic mutants. *Escherichia coli* C7 NADAR serves as a negative control. (**F**) Comparison of PARP10 catalytic preference for thymine (left) versus deoxyuridine (right) within otherwise identical ‘*E21*’ ssDNA contexts, including the reversal of modifications by human TARG1 and *T. aquaticus* DarG. For all figures in this panel, the ‘+ control’ represents oligonucleotides subjected to PARP10-catalysed ADP-ribosylation in the presence of its co-substrate NAD^+^. In this corresponding ‘- control’, PARP10 was replaced with an equivalent volume of reaction buffer, while all other reaction components were kept identical.

To further investigate the preference of PARP10 for DNA versus RNA containing thymine or uracil, we utilized a 15-nt ssDNA oligonucleotide with a single thymine base in the middle, flanked by adenine (‘*Single T*’), compared to an equivalent ssRNA oligonucleotide containing uracil at the same position (‘*Single U*’). We observed that the uracil-containing RNA oligonucleotide (‘*Single U*’) was preferentially ADP-ribosylated by PARP10 compared to thymine-containing DNA oligonucleotide (‘*Single T*’) (Fig. 2A). ADP-ribosylation of the ‘*Single U*’ ssRNA manifested as a single upper band, suggesting that PARP10 catalyses uracil mono-ADP-ribosylation, and ladders observed above (Fig. 1) resulted from simultaneous ADP-ribosylation of multiple uracil bases. The reaction products were further analysed by Matrix-Assisted Laser Desorption/Ionization–Time of Flight (MALDI-TOF) mass spectrometry, which confirmed that 34% of ‘*Single U*’ ssRNA was modified with a single ADP-ribose unit in the conditions used (Fig. 2C; Supplementary Fig. S2D–F, and Supplementary Table S2). Under the same conditions, only 4% of ADP-ribosylation was observed on the single thymine-containing (‘*Single T*’) ssDNA oligonucleotide (Fig. 2B, Supplementary Fig. S2A–C, and Supplementary Table S2). These data suggest that, despite the chemical similarity between thymine and uracil, PARP10-catalysed ADP-ribosylation preferentially targets uracil in ssRNA over thymine in ssDNA. Next, given that PARP10 preferentially modifies uracil-containing ssRNA over thymine-containing ssDNA, we assessed its nucleic acid binding ability through electrophoretic mobility shift assays on native polyacrylamide gels. The ART of PARP10 bound efficiently to both ssDNA and ssRNA 21-nt substrates (‘*E21*’), with possibly slightly higher binding efficiency for RNA (Supplementary Fig. S3). To further investigate the PARP10 preference for RNA over DNA, deoxyuridine (dU) was incorporated into a 21-nt ssDNA oligonucleotide (‘*E21*’ dU) and compared with a thymine-containing control (‘*E21’* ssDNA). Consistently, the dU-containing oligonucleotide was more efficiently ADP-ribosylated, confirming that PARP10 preferentially targets uracil over thymine, even when uracil is present as dU in an ssDNA oligonucleotide (Fig. 3F and Supplementary Fig. S4A). In contrast, free uridine monophosphate nucleotide could not be significantly modified by PARP10 (Supplementary Fig. S4B). Next, to determine whether double-stranded nucleic acids could also serve as substrates for PARP10, we annealed Cy3-labelled ‘*E21*’ DNA and RNA oligonucleotides with their complementary sequences to generate dsDNA, dsRNA, and RNA/DNA hybrids (Supplementary Fig. S5A and B). ADP-ribosylation was markedly diminished on these double-stranded substrates compared with single-stranded nucleic acids (Supplementary Fig. S5B), indicating that PARP10 preferentially targets accessible, unpaired bases and hindered in base-paired contexts.

### Reversal of the PARP10-catalysed RNA ADP-ribosylation of uracil bases

We next asked whether any ADP-ribosylhydrolases could remove ADP-ribose from oligonucleotides modified by PARP10. To assess this, both ADP-ribosylated 15-nt *‘15U’* and 21-nt *‘E21’* ssRNA oligonucleotides were tested with a panel of purified, recombinant human hydrolases (Fig. 3A and B). Robust hydrolytic activity was observed for TARG1, whereas other human hydrolases exhibited no or only minimal activity (Fig. 3A and B). Human TARG1 is well established in hydrolysing ADP-ribose from thymine bases in DNA [29], and our results now reveal that it is also active on uracil bases in RNA. Furthermore, we tested two bacterial macrodomain-containing hydrolases previously shown to act on thymine-linked ADP-ribosylation: the DarG antitoxin from *T. aquaticus* [17] and its divergent homologue, SCO6735 from *S. coelicolor* [30]. Both enzymes are hydrolytically active on the ADP-ribosylated ssRNA substrates catalysed by PARP10 (Fig. 3A and B), with *T. aquaticus* DarG exhibiting notably lower hydrolytic efficiency compared with human TARG1 and SCO6735 (Fig. 3C and D). By contrast, the NADAR-domain ADP-ribosylhydrolase from *E. coli* (C7 NADAR), which was previously characterized for its hydrolytic activity toward guanine bases [6, 44], was hydrolytically inactive on PARP10-catalysed ssRNA oligonucleotides (Fig. 3E). Catalytic mutants of human TARG1 and *T. aquaticus* DarG abolished their hydrolytic activity on PARP10-catalysed ADP-ribosylated ‘*15U*’ ssRNA oligonucleotides compared with the wild-type enzymes (Fig. 3E). In addition, both human TARG1 and *T. aquaticus* DarG exhibited robust hydrolytic activity on ADP-ribosylated dU-containing ‘*E21*’ ssDNA oligonucleotides, comparable to that observed on ADP-ribosylated oligonucleotides containing thymine catalysed both by PARP10 (Fig. 3F).

### Chemical synthesis of both anomers of U-ADPr

**Figure 4.**
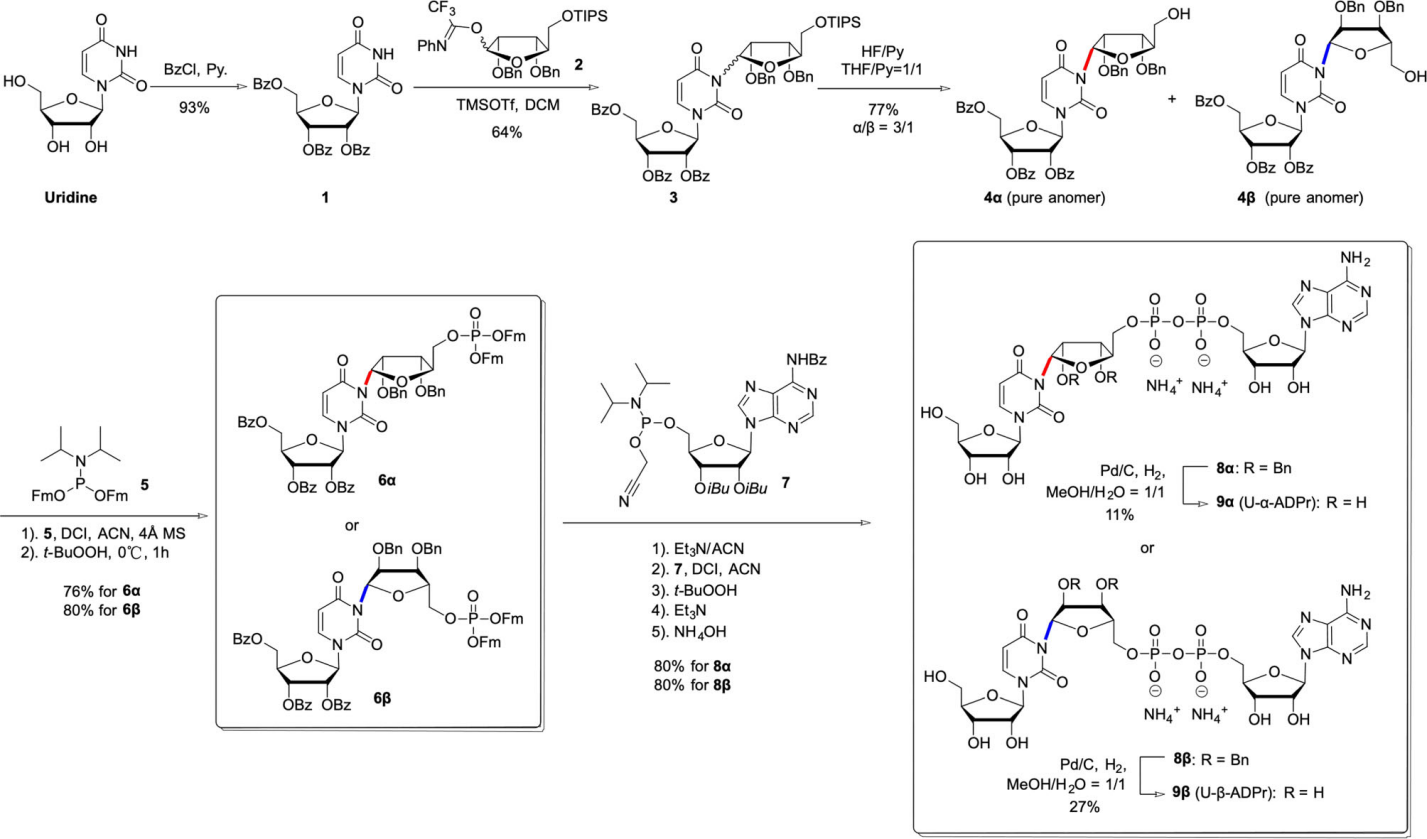
Chemical synthesis of U-ADPr affording both anomers, **9α** (U-α-ADPr) and **9β** (U-β-ADPr). Bz = benzoyl, TIPS = triisopropylsilyl, Bn = benzyl, Fm = 9-fluorenylmethyl, *iBu *= iso-butyryl, DCI = 4,5-dicyanoimidazole.

The most likely site of ADP-ribosylation within the uracil base is the imide *N*^3^ nitrogen, which is known to serve as an acceptor of another modification, methylation [45], and corresponds to the *N*^3^ of thymine, the site of DarT2-catalysed ADP-ribosylation [18]. Tools for studying ADP-ribosylation on uracil bases are currently lacking, so we chemically synthesized *N*^3^-ADP-ribosylated uridine (U-ADPr) for the first time to facilitate the characterization of its biochemical activities and future structural studies. The efficient chemical synthesis of well-defined U-ADPr in both anomeric forms (U-α-ADPr and U-β-ADPr) is essential for biological studies; however, the rare ribosyl imide linkage and the labile pyrophosphate unit render U-ADPr particularly challenging to construct. Building on our previously established stereocontrolled synthesis of a thymidine-ADPr probe [37], we report here the application of a similar strategy to the assembly of U-ADPr. As shown in Fig. 4, an imidate donor was employed to construct *N*^3^-ribosylated uridine, followed by formation of the pyrophosphate linkage via P(III)–P(V) coupling. The synthesis began with perbenzoylation of uridine to afford acceptor **1**, which underwent *N^3^*-ribosylation with the highly reactive *N*-phenyl-2,2,2-trifluoroacetimidate donor **2** [37, 46] to give **3** as an α/β mixture. After silyl deprotection with HF–pyridine, the anomers were separated to yield **4α** and **4β** (α/β ≈ 3:1, 77%). These anomers were advanced separately to obtain stereochemically defined U-ADPr. Notably, attempts to remove benzyl groups at earlier stages led to complications during global deprotection [see Supplementary Information (Chemical Synthesis Dataset)]; thus, benzyl cleavage was deferred to the final step.

The 5-OH of **4α/β** was phosphorylated with phosphoramidite 5 [47] and activated by 4,5-dicyanoimidazole (DCI); subsequent oxidation with *t*-BuOOH afforded phosphotriesters **6α/β** in 76% and 80% yield, respectively. Removal of the Fm groups with Et₃N generated the phosphomonoesters, which were coupled with adenosine phosphoramidite **7** [48] to form P(V)–P(III) intermediates. *In situ* oxidation furnished the pyrophosphate bearing a 2-cyanoethyl (CE) protecting group, which was removed with Et₃N, followed by ammonolysis of the remaining ester groups to give benzyl-protected U-ADPr **8α** and **8β** in excellent yields (80%, five steps). Final catalytic hydrogenolysis over Pd/C produced U-α-ADPr (**9α**) and U-β-ADPr (**9β**). Although partial reduction of the uracil ring was unavoidable, the desired products were readily purified by reverse-phase C18 chromatography to provide analytically pure material. In summary, this route enables efficient, stereo-controlled, milligram-scale synthesis of both anomeric U-ADPr molecules suitable for biological evaluation.

### Synthetic U-ADPr as a tool for characterizing ADP-ribosylhydrolases

**Figure 5.**
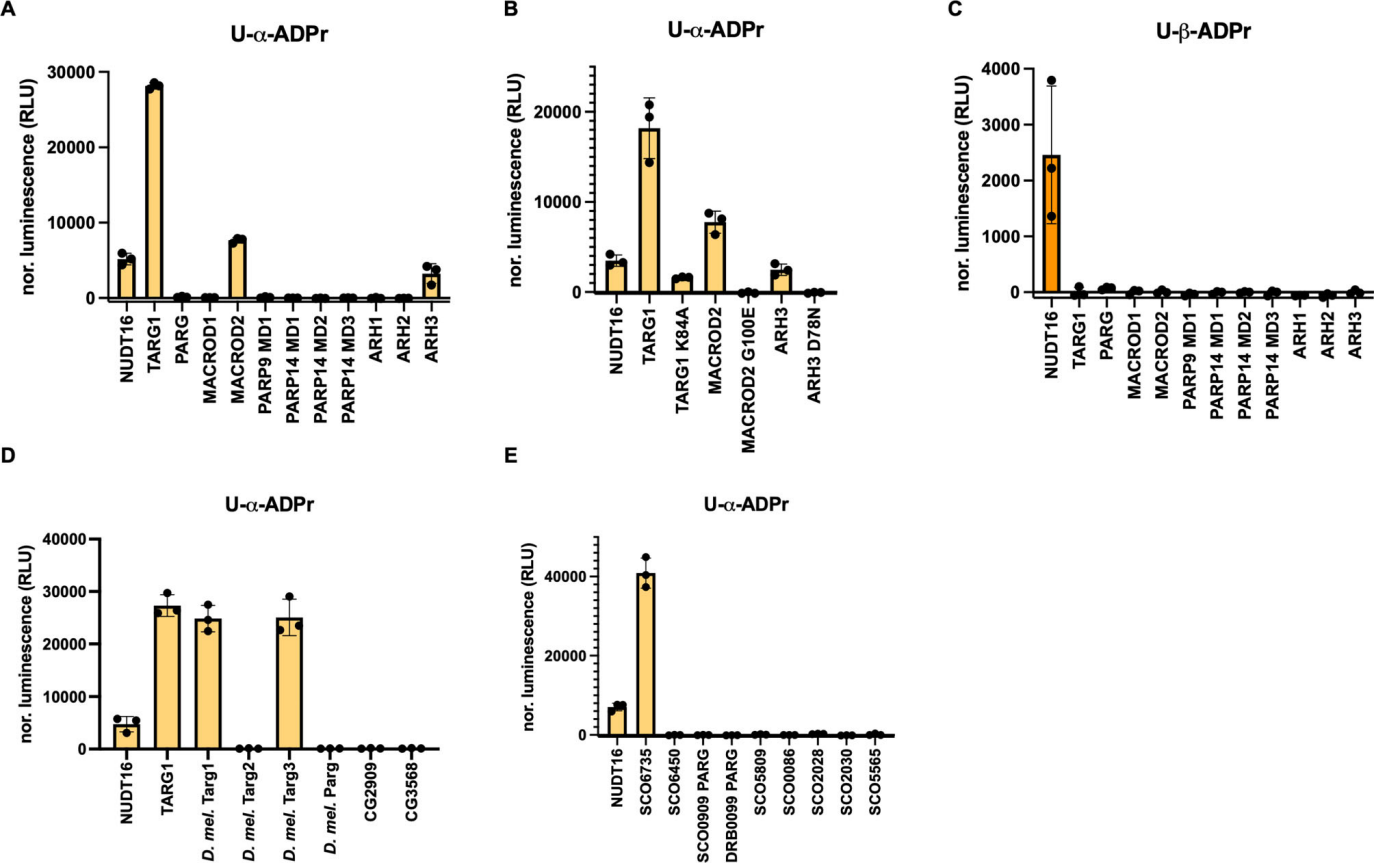
Hydrolytic activity of selected enzymes against chemically synthesized U-ADPr, assessed using the AMP-Glo assay. Panels (A–C) show human hydrolases: panels (**A**) and (**C**) illustrate activities against the α- and β-anomers of U-ADPr, respectively, and panel (**B**) compares the hydrolytic activities of wild-type TARG1, MACROD2, and ARH3 on U-α-ADPr with their catalytic mutants. Panel (**D**) shows *D. melanogaster* hydrolases, and panel (**E**) shows selected bacterial hydrolases from *S. coelicolor* and *D. radiodurans*. Hydrolytic activities were assessed by monitoring AMP release, either directly via NUDT16 as a positive control or indirectly by converting hydrolase-released ADP-ribose to adenosine monophosphate via NUDT5.

Having successfully synthesized both anomers of U-ADPr, we next aimed to further characterize the activities of a panel of human hydrolases previously tested on PARP10-modified oligonucleotides, as shown in Fig. 3A, using our pre-established AMP-Glo assay [38, 49] (Fig. 5A–C). Physiological ADP-ribose attachment is expected to generate the U-α-ADPr anomer through inversion of stereochemistry at the ribose C1'' position [1]. Therefore, alongside the α-anomer, we also included U-β-ADPr as a control to evaluate hydrolase specificity for the α-anomer. Furthermore, Nudix hydrolase 16 (NUDT16) was included as a positive technical control, as it hydrolyses the pyrophosphate linkage independently of the ADP-ribose conjugation [50]. Consistently, we observed strong hydrolytic activity with human TARG1 against U-α-ADPr (Fig. 5A). Human MACROD2 and ARH3 showed low but detectable activity (Fig. 5A). These hydrolytic activities were real, as they were abolished with the corresponding catalytic mutants compared with the wild-type enzymes [51, 52] (Fig. 5B). No other human hydrolases showed detectable activity on the chemically synthesized U-α-ADPr probe (Fig. 5A). Remarkably, all tested human hydrolases were inactive on the U-β-ADPr anomer, highlighting their preference for the α-anomer (Fig. 5C). The U-α-ADPr anomer is the likely preferred product in physiological contexts, as ARTs use β-NAD^+^ as a co-substrate and inverse the stereochemistry at the C1'' position during the reaction [1].

In addition to human hydrolases, we analysed a panel of *D. melanogaster* hydrolases (Fig. 5D). *D. melanogaster* encodes three human TARG1 paralogues, Targ1–3 [34], of which only Targ1 and Targ3 exhibited hydrolytic activity toward U-α-ADPr (Fig. 5D). Other known *D. melanogaster* hydrolases, including Parg [34] and the two divergent macrodomain-containing proteins CG2909 and CG3568 [35], were inactive on U-α-ADPr (Fig. 5D). Finally, we tested a set of bacterial enzymes, focusing on *S. coelicolor*, which encodes an extensive number of hydrolases [36]. Interestingly, only SCO6735 was catalytically active (Fig. 5E), consistent with our observations on the ssRNA oligonucleotide substrates shown in Fig. 3A and B. In contrast, the MACROD homologue SCO6450, PARG-like proteins from *S. coelicolor* (SCO0909) and *Deinococcus radiodurans* (DRB0099), ARH-like proteins SCO5909, SCO0086, SCO2028, and SCO2030, and the NADAR-containing protein SCO5665 all showed no detectable hydrolytic activity on U-α-ADPr (Fig. 5E). Notably, the perfect congruence between the hydrolase sensitivities of enzymatically obtained and synthetically prepared U-α-ADPr supports the conclusion that uracil is ADP-ribosylated by PARP10 at the same atom as in our synthetic probe, *N*^3^.

Collectively, these data demonstrate that our newly developed chemically synthesized U-ADPr anomers provide a valuable tool for the future characterization of proteins involved in uridine-targeted ADP-ribosylation.

## Discussion

ADP-ribosylation of nucleic acids is a widespread chemical modification found across all domains of life. In bacteria, this process is comparatively well characterized, with documented systems that modify DNA bases [6, 17, 18], RNA bases [19, 22], and ribose moieties within RNA [21]. In humans, nucleic acid ADP-ribosylation is much less understood. Although phosphate-linked ADP-ribosylation has been suggested *in vitro*, direct evidence of its existence in cells is lacking [40, 24, 25]. Furthermore, TARG1-sensitive DNA ADP-ribosylation catalysed by PARP1 has been reported at human telomeres, but the precise sites of modification remain unclear [26]. Importantly, to date, no evidence exists for ADP-ribosylation of RNA bases in humans.

In this study, we identify human PARP10 as an enzyme capable of ADP-ribosylation of nucleic acid bases, with its catalytic domain showing a clear catalytic preference for uracil bases in RNA and relatively weaker activity on thymine bases in DNA. This modification preference is further supported by the RNA binding ability of the ART domain of PARP10, highlighting its potential role in RNA modification in humans. We also show that human TARG1 is highly efficient at removing ADP-ribose from uracil bases, consistent with its previously reported activity on thymine bases [29]. Interestingly, *T. aquaticus* DarG exhibits lower hydrolytic efficiency than human TARG1 on uracil-ADP-ribosylation (U-ADPr), despite having comparable activity on thymine-ADP-ribosylation (T-ADPr), suggesting notable specificity for physiological role of bacterial DarTG system for targeting DNA as a toxin–antitoxin system, which is involved in the regulation of DNA replication, transcription, and the anti-phage response [17, 18, 53]. We also confirmed the presence of a U-ADPr hydrolase, SCO6735, in *S. coelicolo*r, one of the richest bacterial genera for ADP-ribosylation proteins [30, 36].

Further studies will be needed to validate the U-ADPr activity of PARP10 in cellular systems, where the regulation is likely to be complex. In addition to its catalytic (ART) domain, full-length PARP10 also contains RNA recognition motifs and K-homology (KH) domains, which are putative DNA/RNA-binding modules, as well as ubiquitin-interacting motifs [4, 54]. These auxiliary domains could possibly contribute to substrate recognition, enhancing both the efficiency and specificity of nucleic acid ADP-ribosylation and potentially directing PARP10 toward particular RNA sequences or structural motifs. Therefore, these features suggest that PARP10 may serve multiple physiological roles through uracil-base targeted ADP-ribosylation of nucleic acids. For example, it could tag misincorporated uridine nucleotides in DNA to modulate replication stress [15] or mark foreign DNA or RNA to influence innate immune responses [10]. Fittingly, PARP10 has a suggested role in translesion DNA synthesis that may be triggered by uracil base incorporation in DNA [55]. Furthermore, CRISPR screens revealed that cells overexpressing PARP10 rely on ataxia-telangiectasia mutated for survival [56]. Beyond potential DNA-associated functions, ADP-ribosylation of AREs in RNA may reshape their interaction landscape, potentially altering the recruitment of RNA-binding proteins such as Human antigen R and tristetraprolin [42, 57, 58]. In addition to affecting protein binding, uracil ADP-ribosylation could potentially modulate susceptibility to cleavage by endonucleases, such as uridine-specific coronavirus nonstructural protein (Nsp15), which processes longer RNA fragments to evade host immune recognition [59, 60]. Such modification may also influence RNA sensing, as RNA ADP-ribosylation could mark foreign RNA and modulate detection by RNA-sensing systems such as Retinoic acid-inducible Gene I [61]. Consistently with a role in antiviral defense, PARP10 has been shown to act as an antiviral enzyme against alphaviruses [14] and may also participate in other RNA-related processes, including stress granule formation and translation inhibition [28, 4]. Our data further indicate that PARP10 preferentially modifies ssRNA (Supplementary Fig. S5), suggesting that only loops or single-stranded regions of structured RNAs may serve as substrates. Dysregulation of the U-ADPr reversal may have physiological consequences, as human TARG1, which we demonstrated here to have a U-ADPr hydrolase activity, is known to bind RNA, interact with proteins involved in RNA metabolism [62], and its deficiency has been linked to severe neurodegenerative disease in humans [63].

Many other questions in the RNA ADP-ribosylation field remain open, and new tools are needed, such as mass spectrometry approaches, sequencing methods that can detect ADP-ribosylation at bases, and specific antibodies to facilitate their answers. Here, we have developed a synthetic U-ADPr probe, enabling faster and more quantitative identification and analysis of hydrolase activities. Our data show that human TARG1 and SCO6735 exhibit robust hydrolytic activity on chemically synthesized U-α-ADPr, consistent with observations on ssRNA oligonucleotide substrates, demonstrating the utility of the probes for defining hydrolase activity and specificity. The *D. melanogaster* hydrolases Targ1 and Targ3 were also newly identified to be active on U-ADPr using this probe. Importantly, the hydrolytic activities of human MACROD2 and ARH3 were revealed using this approach, as these enzymes show only negligible activity in conventional oligonucleotide-based ADP-ribosylation assays with denaturing urea gels, highlighting how this approach overcomes the limitations of previous methods. Overall, our initial hydrolysis activity analysis identified efficient U-ADPr hydrolases across a range of organisms, from bacteria to *D. melanogaster* to humans, suggesting that U-ADPr may be a widespread and evolutionary conserved modification.

Altogether, the findings presented in this study indicate that nucleic acid ADP-ribosylation is more widespread and may have a greater physiological impact than previously recognized. Moreover, the chemically synthesized U-ADPr probes developed here provide a powerful toolset to facilitate further investigations into all these currently unresolved questions.

## Supplementary Material

gkag289_Supplemental_File

## Data Availability

The data underlying this article are available in the article and in its online supplementary material.
