# Supplementary material for: Reversible RNA ADP-ribosylation on uracil bases
Source: Nucleic Acids Res. 2026 Mar 31;54(6):gkag289. doi: 10.1093/nar/gkag289 (PMC13036486; doi:10.1093/nar/gkag289)
Supplement: gkag289_Supplemental_File [file gkag289_supplemental_file.pdf]

Supplementary Information

for

Reversible RNA ADP-ribosylation on uracil bases

Yang Lu[1, +], Li Tang[2, 3, 4, +], Øyvind Strømland[5, +], Chatrin Chatrin[1, +], Kang Zhu[1,6], Deeksha Munnur[1], Joséphine Groslambert[1], Petra Mikolčević[7], Herwig Schüler[8], Gyula Timinszky[9], Guillaume Gabant[10], Marcin J Suskiewicz[10], Andreja Mikoč[7], Vincent Aucagne[10], Dragana Ahel[1], Qiang Liu[2,3,4]*, Ivan Ahel[1]*

[1]Sir William Dunn School of Pathology, University of Oxford, OX1 3RE, United Kingdom

[2]Carbohydrate-Based Drug Research Centre, Shanghai Institute of Materia Medica, Chinese Academy of Sciences, Shanghai, 201203, China

[3]Zhongshan Institute for Drug Discovery, Chinese Academy of Sciences, Zhongshan, 528400, China

[4]University of the Chinese Academy of Sciences, Beijing, 100049, China

[5]Department of Biomedicine, University of Bergen, 5020, Norway

[6]Health Science Centre, East China Normal University, Shanghai, 200241, China

[7]Division of Molecular Biology, Ruđer Bošković Institute, Zagreb,10000, Croatia

[8]Division of Biochemistry and Structural Biology, Department of Chemistry, Lund University, SE-22362, Lund, Sweden

[9]Laboratory of DNA Damage and Nuclear Dynamics, Institute of Genetics, HUN-REN Biological Research Centre, Szeged, 6726, Hungary

[10]Centre de Biophysique Moléculaire (CBM), Orléans, CNRS, UPR 4301, France

[+]These authors contributed equally to this work.

*Corresponding authors: liuqiang@simm.ac.cn, ivan.ahel@path.ox.ac.uk

Supplementary Table S1. DNA/RNA oligonucleotide substrates used in this study.

Oligonucleotide Name	Nucleic Acid Type	Sequence (5'-3')	Used in Figure
'*E21*' ssRNA	ssRNA	5'-Cy3-GUGGCGCGGAGACUUAGAGAA-3'	1A, C; 3B, D; S3; S4A; S5A, B
'*E21*' ssDNA	ssDNA	5'-Cy3-GTGGCGCGGAGACTTAGAGAA-3'	3F; S4A; S5A, B
'*E21 dU*'	ssDNA	5'-Cy3-GdUGGCGCGGAGACdUdUAGAGAA-3'	3F; S4A
'*E21*' dsRNA	dsRNA	5'-Cy3-GUGGCGCGGAGACUUAGAGAA-3'<br>3'-CACCGCGCCUCUGAAUCUCUU-5'	S5A, B
'*E21*' dsRNA/DNA hybrid	dsRNA/DNA hybrid	5'-Cy3-GUGGCGCGGAGACUUAGAGAA-3'<br>3'-CACCGCGCCTCTGAATCTCTT-5'	S5A, B
'*E21*' dsDNA	dsDNA	5'-Cy3-GTGGCGCGGAGACTTAGAGAA-3'<br>3'-CACCGCGCCTCTGAATCTCTT-5'	S5A, B
'*PolyUA*'	ssRNA	5'-Cy3-AUAAUUUAUUUAAUAUA-3'	1B, C
'*15U*'	ssRNA	5'-Cy3-UUUUUUUUUUUUUUU-3'	1D; 3A, C, E; S1
'*5Phos-15U-3Cy3*'	ssRNA	5'-Phos-UUUUUUUUUUUUUUU-Cy3-3'	S1
'*5Cy3-15U-3Phos*'	ssRNA	5'-Cy3-UUUUUUUUUUUUUUU-Phos-3'	S1
'*15C*'	ssRNA	5'-Cy3-CCCCCCCCCCCCCCC-3'	1D
'*15A*'	ssRNA	5'-Cy3-AAAAAAAAAAAAAAA-3'	1D
'*Single T*'	ssDNA	5'-Cy3-AAAAAAATAAAAAAA-3'	2A; S2; Table S2
'*Single U*'	ssDNA	5'-Cy3-AAAAAAAUAAAAAAA-3'	2A; S2; Table S2

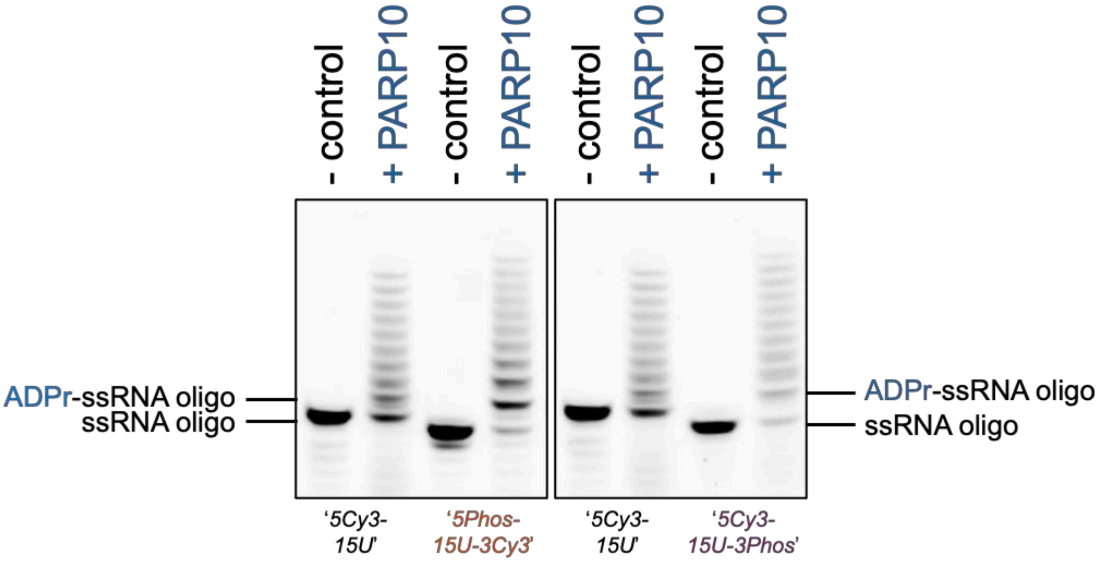

Supplementary Figure S1. PARP10 ADP-ribosylation activity was compared on Cy3-labeled ssRNA oligonucleotides, all containing the same '*15U*' sequence and differing in the presence and position of terminal phosphate (5' or 3'), using gel-based ADP-ribosylation activity assays. In the '− control', PARP10 was replaced with an equal volume of reaction buffer, while all other reaction conditions were kept the same.

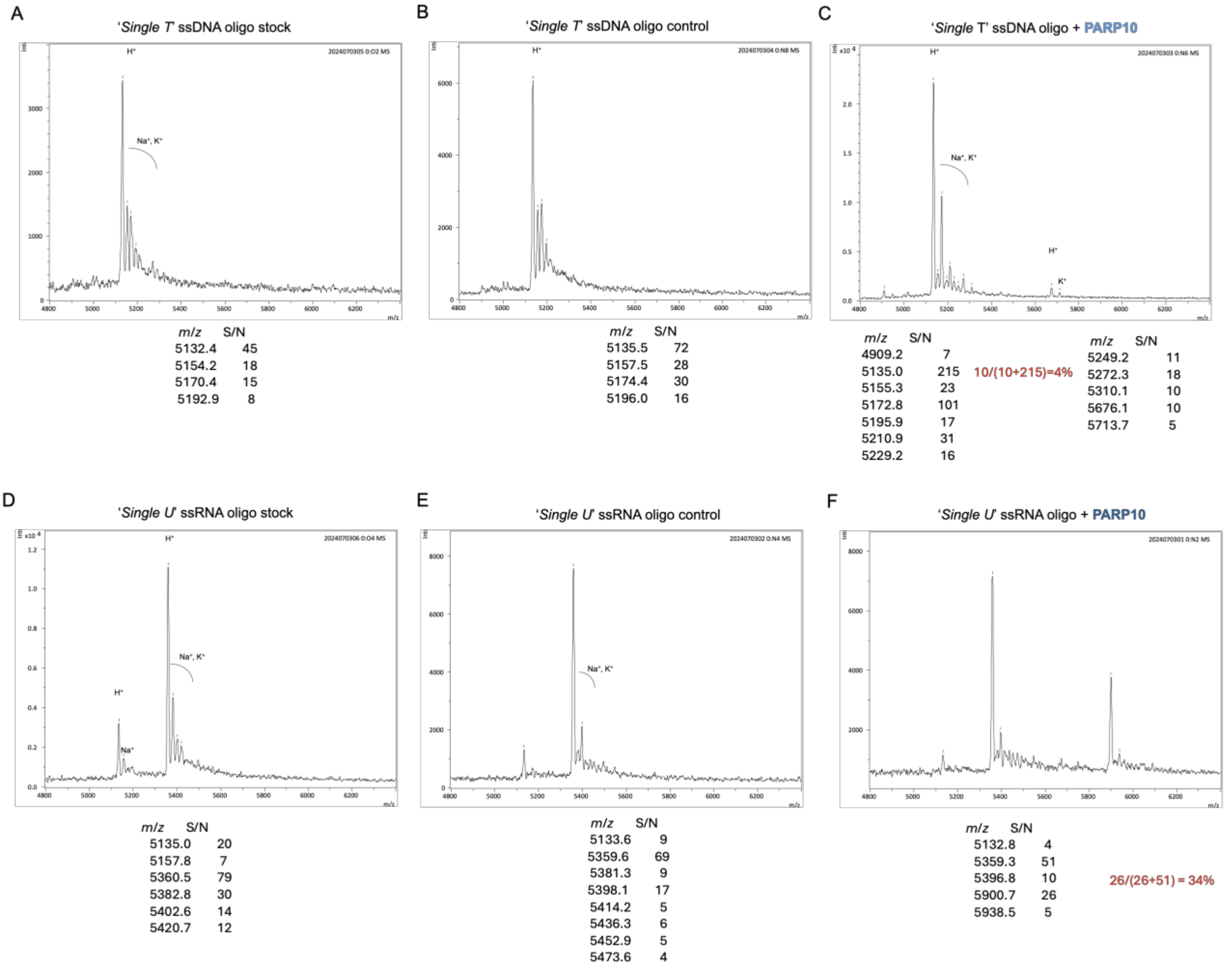

Supplementary Figure S2. Zoom on the MALDI-TOF-MS mass spectra of '*Single-T*' ssDNA stock solution **(A)**, control incubation of '*Single-T*' ssDNA with NAD$^+$ **(B)**, reaction of '*Single-T*' ssDNA with NAD$^+$ in the presence of PARP10, **(C)**, of '*Single-U*' ssRNA stock solution **(D)**, control incubation of '*Single-U*' ssRNA with NAD$^+$ **(E)**, reaction of '*single-U*' ssRNA with NAD$^+$ in the presence of PARP10 **(F)**. S/N: signal to noise ratio. Intens.: signal intensity.

Supplementary Table S2. Calculated and experimentally determined [M]$^+$ values (average mass).

Compound	Molecular formula	Calculated average mass [M]$^+$	Experimental average mass [M]$^+$	Calculated ΔM	Experimental ΔM
ssDNA '*Single T*' oligo	$C_{179}H_{218}N_{74}O_{79}P_{15}^+$	5134.8	5135.0	541.3	541.1
ADPr-ssDNA '*Single T*' oligo	$C_{194}H_{239}N_{79}O_{92}P_{17}^+$	5676.1	5676.1		
ssRNA '*Single U*' oligo	$C_{178}H_{216}N_{74}O_{94}P_{15}^+$	5360.7	5359.3	541.3	541.4
ADPr-ssRNA '*Single U*' oligo	$C_{193}H_{237}N_{79}O_{107}P_{17}^+$	5902.0	5900.7		

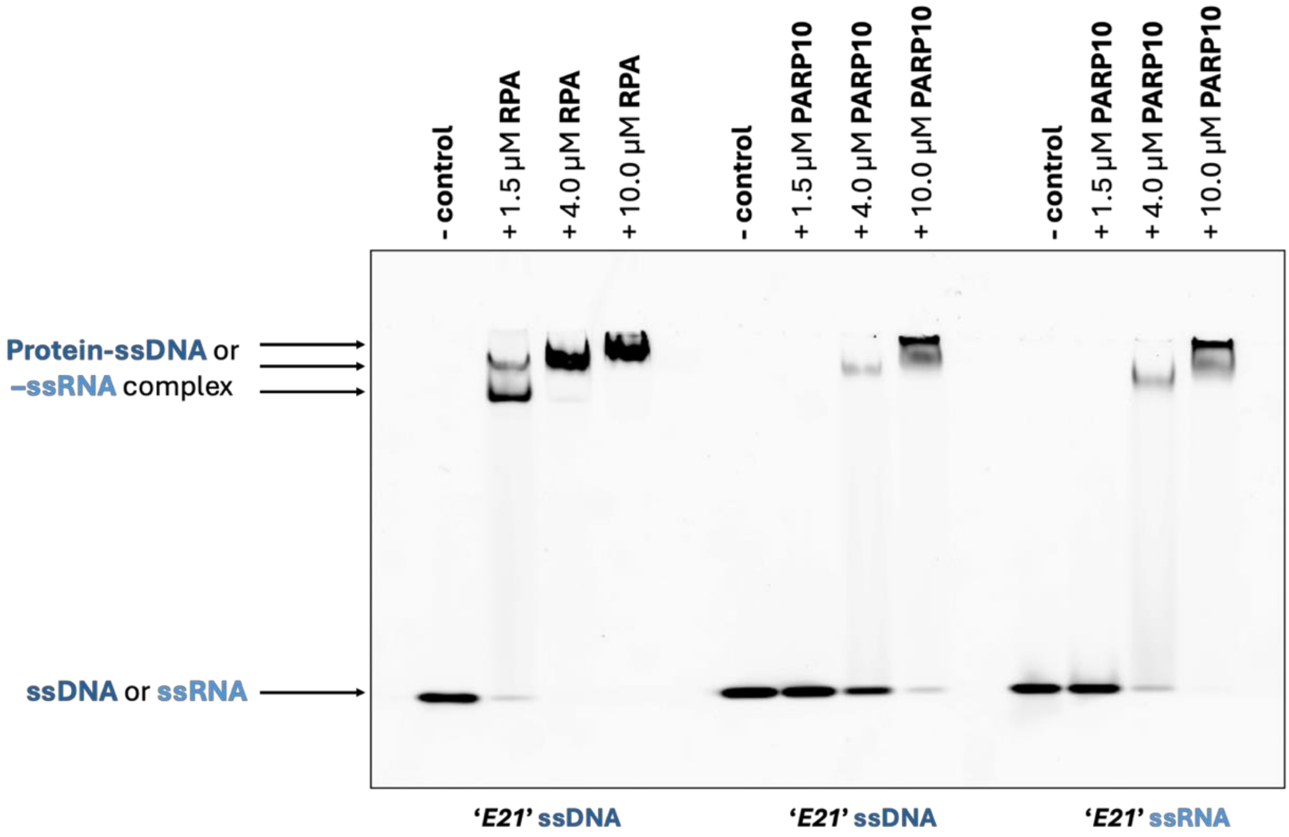

Supplementary Figure S3. EMSA showing the binding of PARP10 to '*E21*' ssDNA and ssRNA oligonucleotides at increasing concentrations (1.5, 4, and 10 µM). Replication Protein A (RPA), a ssDNA binding protein, was included as a positive control.

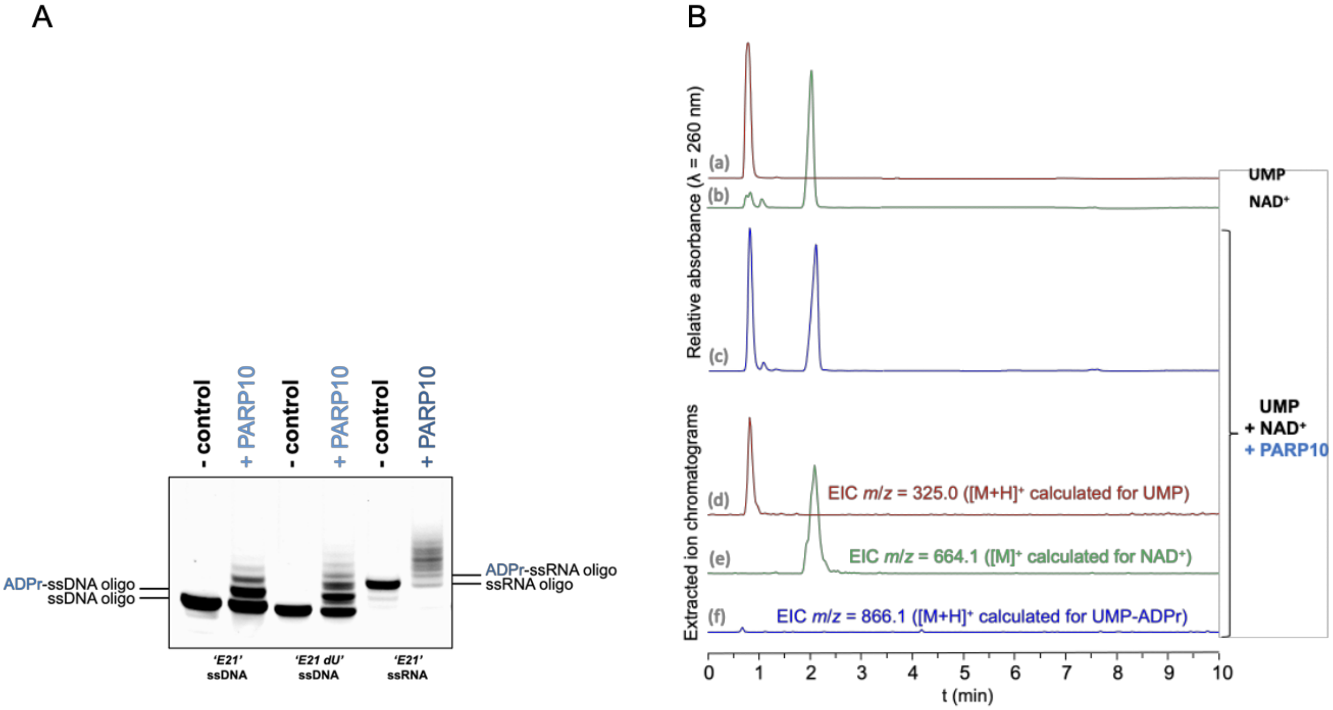

Supplementary Figure S4. (A) Comparison of PARP10 ADP-ribosylation activity on '*E21*' sequence oligonucleotides (Supplementary Table S1), including T-containing ssDNA, dU-containing ssDNA, and U-containing ssRNA, using an *in vitro* gel-based ADP-ribosylation activity assay. **(B)** LC-MS analysis of the attempt of PARP10-mediated ADP-ribosylation of uridine monophosphate (UMP).

Traces (a) and (b): starting co-substrates UMP and NAD^+, respectively (UV, λ = 260 nm). Trace (c): crude reaction mixture (UV, λ = 260 nm), showing the absence of apparition of novel HPLC peaks upon incubation with the enzyme. Trace (d), (e) and (f): extracted ion chromatogram (EIC) for co-substrates UMP and NAD^+, and product UMP-ADPr, respectively. The small peaks in the UMP-ADPr EIC are random baseline noise: examination of all the corresponding mass spectra dismiss the possibility of one corresponding to small amount of the product formed.

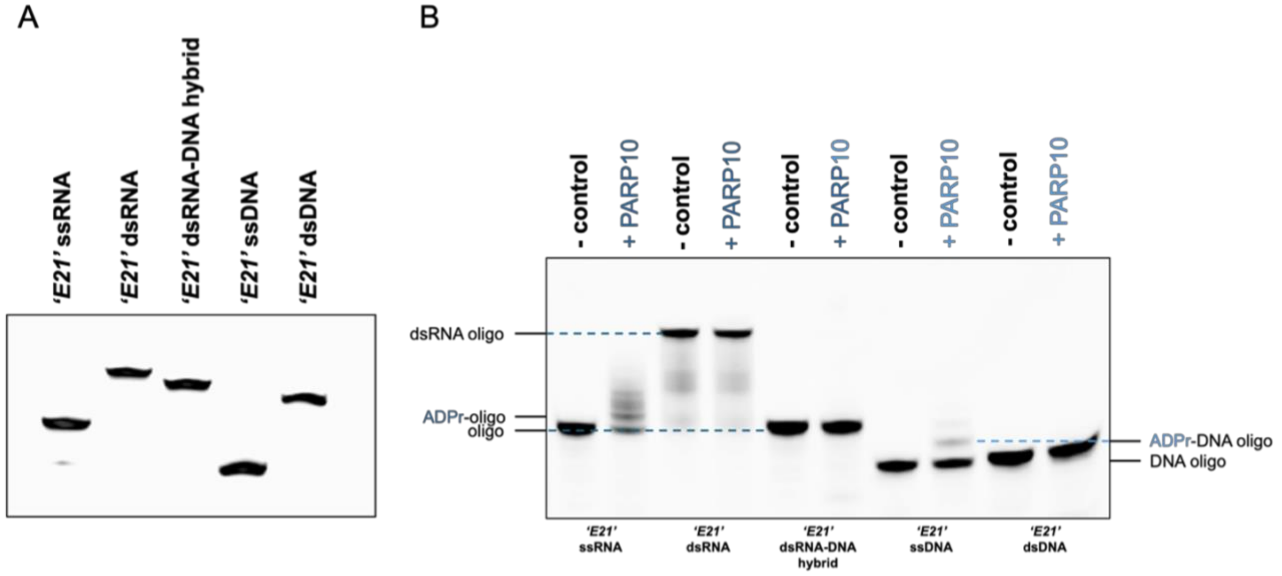

Supplementary Figure S5. Comparison of PARP10 substrate preference for single-stranded and double-stranded nucleic acid oligonucleotides. (A) Native 20% polyacrylamide gel electrophoresis analysis of annealed oligonucleotides alongside their single-stranded counterparts to confirm duplex formation prior to enzymatic assays. **(B)** Comparison of PARP10-catalysed ADP-ribosylation activity on single-stranded oligonucleotides and the corresponding double-stranded substrates (Sequences are listed in Supplementary Table S1), including RNA/DNA hybrid duplexes, assessed using an *in vitro* gel-based ADP-ribosylation activity assay.

Supplementary Information

Chemical Synthesis Dataset

for

Reversible RNA ADP-ribosylation on uracil bases

General Methods

All chemicals were purchased from commercial suppliers and used without further purification unless otherwise stated. Anhydrous solvents were dried over 3 Å or 4 Å molecular sieves prior to use. Solvents used for extraction and chromatography were of technical or HPLC grade. Reactions were monitored by TLC using silica gel plates (F254). Visualization was performed under UV (254 nm) and/or by staining with sulfuric acid–methanol or ceric ammonium molybdate followed by heating. Silica column chromatography was carried out on silica gel (300 mesh, LookChem) or automated using a SepaBean T system equipped with a silica column (40-60 µm, 60 Å). Size-exclusion chromatography was performed on SephadexTM LH-20 (DCM/MeOH = 1/1 as the eluent) or Bio-Gel® P2 (H_2O as the eluent). ^1H, ^{13}C, and 2D NMR spectra (COSY, HSQC, HMBC, NOESY when required) were recorded at 500 or 600 MHz in $CDCl_3$, or D_2O. Full spectra are shown in Supplementary Figures S1-S79. LC-MS analysis was performed on an Agilent 1260 Infinity II-6125 system using an analytical C18 column (3 × 150 mm, 2.8 µm) and a binary solvent system of water (A) and acetonitrile (B). Detection was monitored at 254 and 360 nm. High-resolution mass spectrometry (HRMS) data were acquired on a Thermo Scientific Exactive Plus Orbitrap mass spectrometer equipped with electrospray ionization (ESI) in positive and negative modes. Data processing was conducted using Thermo Xcalibur software. Characterization data are reported without separation of diastereomers unless otherwise required for structural assignment. Full synthetic procedures and characterization (NMR, LC-MS, HRMS) for all intermediates and final compounds are provided in the following sections.

U-α-ADPr using route A

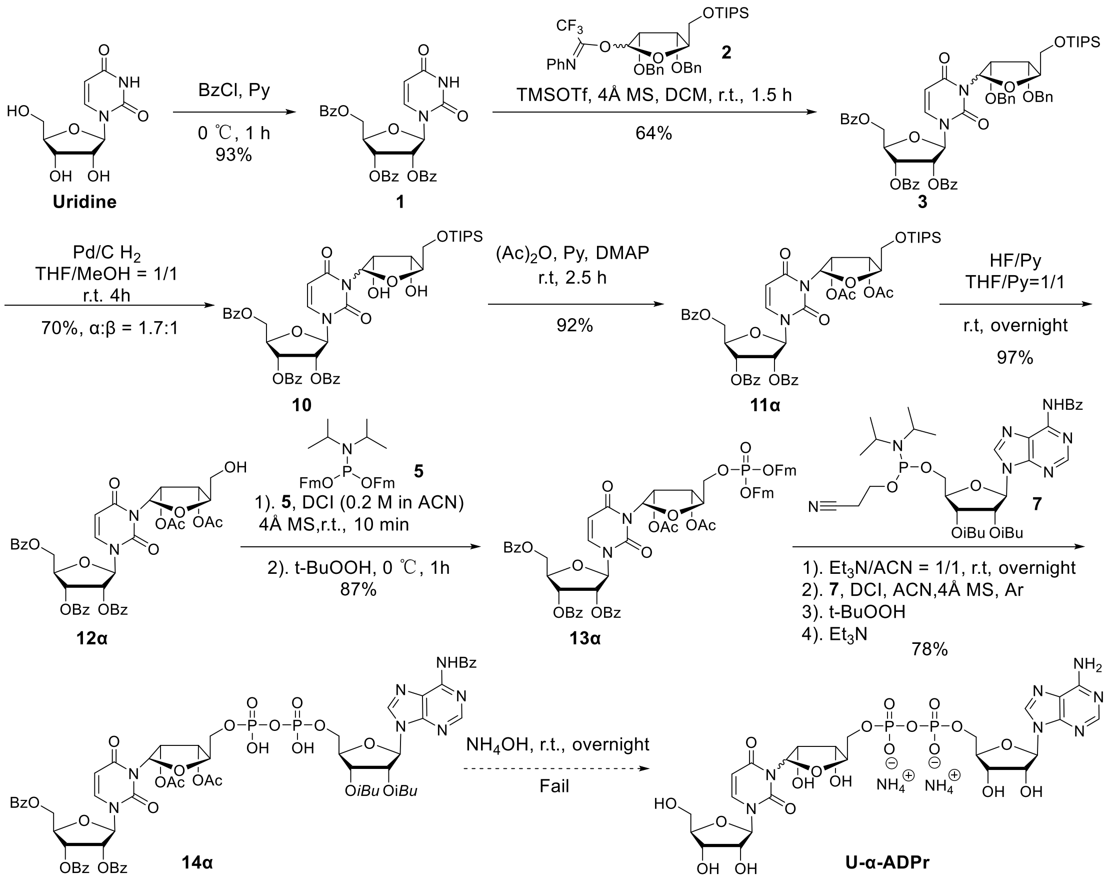

Scheme S1. fail to synthesize U-α-ADPr by route A

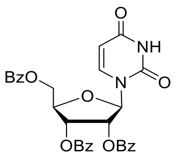

1-(2′,3′,5′-tri-*O*-benzoyl-β-D-ribofuranosyl)-uracil (1)

The uridine (4.88 g, 20.00 mmol) in pyridine (80.0 mL) at 0 °C was treated with BzCl (7.7 mL, 66 mmol). Then the reaction mixture was stirred vigorously for 1 h. The reaction mixture was quenched with MeOH, evap. in vacuo. The residue was further purified by silica gel chromatography (DCM/EA = 100/0 – 1/1) to afford compound **1** (10.32 g, 18.56 mmol, 93%) as white solid. Spectral data was in accordance with literature(1). **Rf** = 0.6 (PE/EA = 1/1). **^{1}H NMR** (500 MHz, DMSO-*d*$_6$) δ 11.52 (s, 1H, NH), 8.01 (dd, *J* = 8.3, 1.4 Hz, 2H), 7.89 (ddd, *J* = 11.9, 8.4, 1.4 Hz, 4H), 7.84 (d, *J* = 8.1 Hz, 1H, H5), 7.68 – 7.62 (m, 3H), 7.53 – 7.48 (m, 2H), 7.47 – 7.42 (m, 4H), 6.16 (d, *J* = 3.9 Hz, 1H), 5.96 – 5.89 (m, 2H), 5.68 (d, *J* = 8.0 Hz, 1H), 4.77 – 4.68 (m, 2H), 4.64 (dd, *J* = 11.9, 5.5 Hz, 1H).

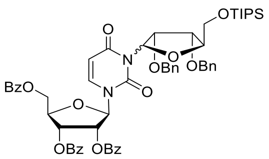

1-(2′,3′,5′-tri-*O*-benzoyl-β-D-ribofuranosyl)-3-[2″,3″-di-*O*-benzyl-5″-*O*-(triisopropylsilyl)-α/β-D-ribofuranosyl]-uracil (3)

Compound **1** (2.0 g, 3.6 mmol) and **2**(2) (4.7 g, 7.2 mmol) were co-evaporated with toluene (3 ×). Dry DCM (2 mL) and freshly activated 4Å molecular sieves (3.6 g) were added to the mixture. The mixture was stirred under argon at room temperature for 0.5 h. Next, TMSOTf (130 μL, 0.7 mmol) was added. The reaction mixture was stirred at room temperature for 1.5 h and then was quenched by addition of triethylamine. The reaction mixture was concentrated under reduced pressure and purified by silica gel chromatography (PE/EA = 10/1 – 4/1) to obtain compound **3** as a white foam and as α/β = 3/1 anomeric mixture (2.4 g, 2.3 mmol, 64%). **Rf** = 0.5 (PE/EA = 4/1). β isomer: **^{1}H NMR** (600 MHz, Chloroform-*d*) δ 8.11 (d, *J* = 9.8 Hz, 2H, arom.), 7.99 (d, *J* = 9.6 Hz, 2H, arom.), 7.92 (d, *J* = 7.2 Hz, 2H, arom.), 7.64 – 7.57 (m, 2H, arom.), 7.54 (d, *J* = 7.5 Hz, 1H, arom.), 7.50 (t, *J* = 7.8 Hz, 2H, arom.), 7.41 (t, *J* = 7.8 Hz, 2H, arom.), 7.35 (d, *J* = 5.9 Hz, 3H, arom., H6), 7.33 – 7.26 (m, 8H, arom.), 7.26 – 7.21 (m, 2H, arom.), 6.43 (d, *J* = 3.0 Hz, 1H, H1"), 6.23 (d, *J* = 5.1 Hz, 1H, H1'), 5.86 (t, *J* = 5.5 Hz, 1H, H3'), 5.72 (t, *J* = 5.6 Hz, 1H, H2'), 5.56 (d, *J* = 8.1 Hz, 1H, H5), 4.83 (dd, *J* = 12.3, 2.8 Hz, 1H, H5'), 4.75 – 4.71 (m, 1H, H4'), 4.67 (dd, *J* = 12.2, 3.8 Hz, 1H, H5'), 4.57 – 4.39 (m, 5H, H2", Bn-CH$_2$), 4.29 (s, 1H, H3"), 4.11 – 4.06 (m, 1H, H4"), 3.88 (dd, *J* = 11.0, 4.1 Hz, 1H, H5"), 3.80 (dd, *J* = 11.1, 5.8 Hz, 1H, H5"), 1.03 (m, 21H, TIPS). **^{13}C NMR** (151 MHz, Chloroform-*d*) δ 166.2, 165.4, 165.4, 161.7, 138.3, 137.8, 133.9, 133.9, 130.1, 130.0, 129.8, 129.3, 128.9, 128.8, 128.7, 128.7, 128.5, 128.4, 128.4, 128.0, 128.0, 127.7 (arom.), 103.0 (C5), 88.9 (C1'), 87.8 (C1"), 83.2 (C4"), 80.6 (C4'), 77.7 (C2'), 77.3 (C3"), 74.1 (C2'), 72.5 (Bn-CH$_2$), 71.2 (C3'), 64.0 (C5"), 63.7 (C5'), 18.1 (TIPS), 12.1 (TIPS). **HR-MS (ESI) m/z:** [M+Na]$^+$ Calcd for $C_{58}H_{64}N_2O_{13}Si$ 1047.4070, Found 1047.4072. α isomer: **^{1}H NMR** (500 MHz, Chloroform-*d*) δ 8.10 (d, *J* = 7.7 Hz, 2H, arom.), 7.94 (t, *J* = 7.3 Hz, 4H, arom.), 7.54 (m, 5H, arom.), 7.40 – 7.26 (m, 10H, H6, arom.), 7.22 – 7.10 (m, 5H, arom.), 6.56 (d, *J* = 5.7 Hz, 1H, H1″), 6.37 (s, 1H, H1′), 5.87 (t, *J* = 5.3 Hz, 1H, H3′), 5.71 (t, *J* = 5.6 Hz, 1H, H2′), 5.50 (d, *J* = 8.1 Hz, 1H, H5), 4.84 (dd, *J* = 12.1, 2.7 Hz, 1H, H5′), 4.73 – 4.50 (m, 6H, H5′, H4′, H4″ & Bn-CH$_2$), 4.35 (d, *J* = 12.0 Hz, 1H, Bn-CH$_2$), 4.18 (m, 2H, H3″ & H2″), 3.91 (d, *J* = 13.6 Hz, 1H, H5″), 3.73 (dd, *J* = 11.6, 2.4 Hz, 1H, H5″), 1.11 – 0.92 (m, 21H, TIPS). **^{13}C NMR** (151 MHz, Chloroform-*d*) δ 166.2, 165.4, 165.3, 162.6 (C4), 149.4 (C2), 138.3, 137.9, 137.5,

133.8, 133.8, 133.7, 130.1, 130.0, 129.8, 129.4, 128.9, 128.8, 128.8, 128.7, 128.6, 128.6, 128.5, 128.4, 128.0, 127.9, 127.8, 127.7, 102.6 (C5), 88.3 (C1′), 86.3 (C1″), 82.7 (C4″), 80.3 (C4′), 76.6 (C3″), 74.4 (Bn-CH_2), 74.0 (C2′), 72.8 (Bn-CH_2), 71.1 (C3′), 63.8 (C5′), 61.8 (C5″), 18.1 (TIPS), 18.1 (TIPS), 12.0 (TIPS). **HR-MS (ESI) m/z:** $[M+H]^+$ Calcd for $C_{58}H_{64}N_2O_{13}Si$ 1025.4250, Found 1025.4253.

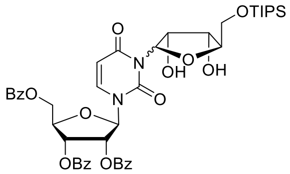

1-(2′,3′,5′-tri-*O*-benzoyl-β-ᴅ-ribofuranosyl)-3-[5″-*O*-(triisopropylsilyl)-α/β-ᴅ-ribofuranosyl]-uracil (10)

A mixture of **3** (2.27 g, 2.22 mmol), 1.14 g of 10% Pd/C in a 1:1 solution of methanol/THF (74 ml) was hydrogenated at room temperature and atmospheric pressure during 4 h. The mixture was filtered through a pad of Celite and the filtrate was concentrated under reduced pressure. The crude material was purified by flash silica gel chromatography (PE/EA = 2/1 – 1/1 – 1/2) to give compound **10** as white foam (α isomer: 832 mg, β isomer: 477 mg, yield = 70%). **Rf** (α isomer) = 0.7 (DCM/MeOH = 30/1). **Rf** (β isomer) = 0.4 (DCM/MeOH = 30/1). α isomer: **^{1}H NMR** (500 MHz, Chloroform-*d*) δ 8.09 (d, *J* = 7.0 Hz, 2H, arom.), 7.99 – 7.93 (m, 4H, arom.), 7.60 (m, 3H), 7.49 (t, *J* = 7.7 Hz, 2H, arom.), 7.46 (d, *J* = 8.1 Hz, 1H, H6), 7.39 (m, 4H, arom.), 6.74 (s, 1H, H1″), 6.33 (d, *J* = 5.2 Hz, 1H, H1′), 5.87 (t, *J* = 5.3 Hz, 1H, H3′), 5.75 (t, *J* = 5.5 Hz, 1H, H2′), 5.65 (d, *J* = 8.1 Hz, 1H, H5), 4.84 (dd, *J* = 12.3, 2.8 Hz, 1H, H5′), 4.73 (d, *J* = 3.8 Hz, 1H, H4′), 4.69 – 4.62 (m, 2H, H5′ & H3′-OH), 4.56 (q, *J* = 7.3 Hz, 1H, H2″), 4.35 – 4.29 (m, 1H, H3″), 4.24 (m, 1H, H4″), 3.79 (d, *J* = 2.5 Hz, 2H, H5″), 3.04 (d, *J* = 8.0 Hz, 1H, H2″-OH), 1.06 (m, 21H, TIPS). **^{13}C NMR** (151 MHz, Chloroform-*d*) δ 166.2, 165.4, 165.4, 163.3, 151.4, 138.1 (C6), 133.9, 133.9, 130.1, 130.0, 129.8, 129.3, 129.0, 128.7, 128.7, 128.6, 103.7 (C5), 88.9 (C1′), 87.539 (C4″), 86.3 (C1″), 80.7 (C4′), 74.1 (C2′), 72.3 (C2″), 72.3 (C3″), 71.1 (C3′), 64.9 (C5″), 63.7 (C5′), 18.1 (TIPS), 18.1 (TIPS), 12.0 (TIPS). **HR-MS (ESI) m/z:** $[M+H]^+$ Calcd for $C_{44}H_{52}N_2O_{13}Si$ 845.3311, Found 845.3311. β isomer: **^{1}H NMR** (500 MHz, Chloroform-*d*) δ 8.09 (d, *J* = 7.7 Hz, 2H, arom.), 7.95 (dd, *J* = 14.5, 7.8 Hz, 4H, arom.), 7.57 (m, 3H), 7.48 (t, *J* = 7.6 Hz, 2H, arom.), 7.42 – 7.34 (m, 5H, H6, arom.), 6.34 (s, 1H, H1"), 6.19 (d, *J* = 4.9 Hz, 1H, H1'), 5.88 (t, *J* = 5.6 Hz, 1H, H3'), 5.75 (t, *J* = 5.5 Hz, 1H, H2'), 5.61 (d, *J* = 8.1 Hz, 1H, H5), 4.82 (dd, *J* = 12.3, 2.8 Hz, 1H, H5'), 4.71 (m, 1H, H4'), 4.66 (dd, *J* = 12.2, 4.0 Hz, 1H, H5'), 4.60 (d, *J* = 6.9 Hz, 2H, H2" & H3"), 3.99 (m, 1H, H5"), 3.90 – 3.83 (m, 2H, H4"),

3.10 (s, 1H, 2"-OH), 2.64 (s, 1H, 3"-OH), 1.05 (d, *J* = 6.6 Hz, 21H, TIPS). **^{13}C NMR** (151 MHz, Chloroform-*d*) δ 166.2, 165.5, 165.4, 161.9, 150.2, 138.5, 134.0, 133.9, 133.8, 130.0, 130.0, 129.8, 129.3, 128.9, 128.8, 128.7, 128.6, 103.2 (C5), 89.7 (C1'), 89.2 (C1"), 82.6 (C4"), 80.5 (C4'), 74.1 (C2'), 72.8 (C2" or C3"), 72.6 (C2" or C3"), 71.2 (C3'), 64.8 (C5"), 63.7 (C5'), 18.1 (TIPS), 12.0 (TIPS). **HR-MS (ESI) m/z:** $[M+H]^+$ Calcd for $C_{44}H_{52}N_2O_{13}Si$ 845.3311, Found 845.3315.

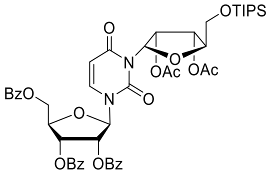

1-(2′,3′,5′-tri-*O*-benzoyl-β-ᴅ-ribofuranosyl)-3-[2″,3″-di-*O*-acetyl-5″-*O*-(triisopropylsilyl)-α-ᴅ-ribofuranosyl]-uracil (11α)

A mixture of **10α** (832 mg, 0.99 mmol), acetic anhydride (928 μL, 9.9 mmol) and DMAP (6 mg, 0.05 mmol) in pyridine (5 ml) was stirred at room temperature for overnight. The mixture was concentrated under reduced pressure. The crude material was purified by silica gel chromatography (PE/EA = 4/1 – 2/1 – 1/1) to give **11α** as white foam (849 mg, 92%.). **Rf** = 0.5 (PE/EA = 2/1). **^{1}H NMR** (500 MHz, Chloroform-*d*) δ 8.12 – 8.06 (m, 2H, arom.), 8.00 – 7.96 (m, 2H, arom.), 7.93 – 7.89 (m, 2H, arom.), 7.62 – 7.53 (m, 3H, arom.), 7.47 (t, *J* = 7.7 Hz, 2H, arom.), 7.40 (t, *J* = 7.8 Hz, 2H, arom.), 7.38 – 7.30 (m, 3H, H6, arom.), 6.78 (d, *J* = 6.7 Hz, 1H, H1″), 6.18 (s, 1H, H1′), 5.89 (t, *J* = 5.6 Hz, 1H, H3′), 5.84 – 5.79 (m, 1H, H2′), 5.60 (d, *J* = 8.1 Hz, 1H, H5), 5.57 (t, *J* = 7.0 Hz, 1H, H2″), 5.46 – 5.38 (m, 1H, H3″), 4.83 (dd, *J* = 12.1, 3.1 Hz, 1H, H5′), 4.75 – 4.70 (m, 2H, H4′ & H4″), 4.65 (dd, *J* = 12.1, 4.3 Hz, 1H, H5′), 3.88 (m, 2H, H5″), 2.07 (s, 3H, OAc), 1.94 (s, 3H, OAc), 1.13 – 1.03 (m, 21H, TIPS). **^{13}C NMR** (151 MHz, Chloroform-*d*) δ 170.4, 169.3, 166.2, 165.5, 165.5, 162.0, 149.8, 138.5, 133.9, 133.9, 133.8, 130.0, 130.0, 129.8, 129.4, 128.9, 128.7, 128.7, 128.6, 128.6 (arom.), 103.0 (C5), 90.0 (C1′), 84.9 (C1″), 84.0 (C4″), 80.4 (C4′), 73.8 (C2′), 71.4 (C3′), 70.7 (C3″), 70.5 (C2″), 63.9 (C5′), 62.9 (C5″), 20.8 (OAc), 20.4 (OAc), 18.1 (TIPS), 18.0 (TIPS), 12.0 (TIPS). **HR-MS (ESI) m/z:** $[M+H]^+$ Calcd for $C_{48}H_{56}N_2O_{15}Si$ 929.3523, Found 929.3525.

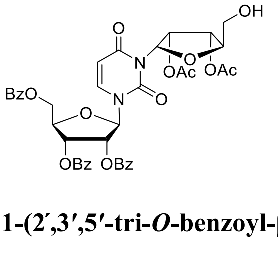

1-(2′,3′,5′-tri-*O*-benzoyl-β-ᴅ-ribofuranosyl)-3-(2″,3″-di-*O*-acetyl-α-ᴅ-ribofuranosyl)-uracil (12α)

Compound **11α** (849 mg, 0.915 mmol) was co-evaporated with toluene (3×), then hook up to oil pump to dryness. Then **11α** was dissolved in pyridine/THF (6 mL, 1/1 v/v) and HF·pyridine (900 μL) was added. The reaction was stirred for 10 h at room temperature. Then the reaction was quenched by aq. $NaHCO_3$ (sat.). The reaction mixture was extracted with EA, the combined organic layer was dried over Na_2SO_4, concentrated under reduced pressure and purified by silica gel chromatography (PE/EA = 2/1 – 1/1 – 1/2) to obtain **12α** (686 mg, 0.89 mmol, 97%). **Rf** = 0.5 (PE/EA = 1/1). **^{1}H NMR** (500 MHz, Chloroform-*d*) δ 8.11 – 8.06 (m, 2H, arom.), 8.02 – 7.97 (m, 2H, arom.), 7.93 – 7.88 (m, 2H, arom.), 7.64 – 7.52 (m, 3H, arom.), 7.47 (t, *J* = 7.8 Hz, 2H, arom.), 7.41 (t, *J* = 7.8 Hz, 2H, arom.), 7.35 (t, *J* = 8.0 Hz, 3H, H6, arom.), 6.82 (d, *J* = 6.4 Hz, 1H, H1″), 6.19 (d, *J* = 5.1 Hz, 1H, H1′), 5.92 – 5.86 (m, 1H, H3′), 5.84 – 5.78 (m, 1H, H2′), 5.62 (d, *J* = 8.1 Hz, 1H, H5), 5.59 (t, *J* = 6.7 Hz, 1H, H2″), 5.34 – 5.27 (m, 1H, H3″), 4.83 (dd, *J* = 12.1, 3.1 Hz, 1H, H5′), 4.77 (m, 1H, H4″), 4.73 (q, *J* = 4.5 Hz, 1H, H4′), 4.67 (dd, *J* = 12.1, 4.3 Hz, 1H, H5′), 3.85 (d, *J* = 10.1 Hz, 1H, H5″), 3.64 (d, *J* = 12.5 Hz, 1H, H5″), 2.08 (s, 3H, OAc), 1.98 (s, 3H, OAc). **^{13}C NMR** (151 MHz, Chloroform-*d*) δ 170.4, 169.3, 166.2, 165.5, 161.9, 149.8, 138.7, 134.0, 134.0, 133.8, 130.0, 130.0, 129.8, 129.4, 128.9, 128.7, 128.7, 128.5, 102.8 (C5), 90.0 (C1′), 84.4 (C1″), 82.6 (C4″), 80.5 (C4′), 73.8 (C2′), 71.5 (C3′), 70.3 (C2″), 70.1 (C3″), 63.9 (C5′), 61.4 (C5″), 20.7 (OAc), 20.5 (OAc). **HR-MS (ESI) m/z:** $[M+H]^+$ Calcd for $C_{39}H_{36}N_2O_{15}$ 773.2188, Found 773.2189

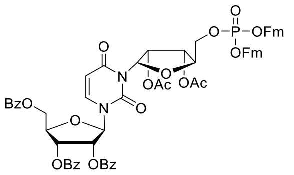

1-(2′,3′,5′-tri-*O*-benzoyl-β-D-ribofuranosyl)-3-[2″,3″-di-*O*-acetyl-5″-*O*-(di-flourenyl)-α-D-ribofuranosyl]-uracil (13α)

Compound **12α** (686 mg, 0.89 mmol), DCI activator (4,5-dicyanoimidozle, 209 mg) and freshly activated 4Å molecular sieves were added into flash. **5** (3) (0.2 M in ACN, 6.6 mL, 1.33 mmol) were added into the mixture and the reaction was stirred for 20 min at room temperature after which *t*-BuOOH (5.5 M in decane, 1.6 mL, 8.86 mmol) was added at 0 ºC. The reaction was stirred at same temperature for 30 minutes and quenched by aq. $NaHCO_3$ (sat.). The mixture was filtered and extracted with EtOAc (3×), washed with brine. The combined organic layer was dried over Na_2SO_4, concentrated under reduced pressure and purified by flash silica gel chromatography (PE/EA = 2/1 – 3/2 – 1/1 – 2/3- 1/2) to **13α**

obtain (938 mg, 87%) as white foam. **Rf** = 0.2 (PE/EA = 1/1). **^{31}P NMR** (202 MHz, Chloroform-*d*) δ -1.61. **^{1}H NMR** (600 MHz, Chloroform-*d*) δ 8.07 (d, *J* = 7.0 Hz, 2H, arom.), 7.98 (d, *J* = 8.5 Hz, 2H, arom.), 7.88 (d, *J* = 7.0 Hz, 2H, arom.), 7.73 – 7.65 (m, 4H, arom.), 7.59 (q, *J* = 6.6, 6.2 Hz, 2H, arom.), 7.50 (m, 7H), 7.40 (t, *J* = 7.7 Hz, 2H, arom.), 7.34 (m, 7H, H6, arom.), 7.27 – 7.23 (m, 4H, arom.), 6.74 (d, *J* = 6.5 Hz, 1H, H1″), 6.17 (d, *J* = 5.0 Hz, 1H, H1′), 5.86 (t, *J* = 5.6 Hz, 1H, H3′), 5.78 (t, *J* = 5.7 Hz, 1H, H2′), 5.58 (d, *J* = 8.1 Hz, 1H, H5), 5.50 (t, *J* = 6.9 Hz, 1H, H2″), 5.20 (t, *J* = 7.4 Hz, 1H, H3″), 4.90 – 4.79 (m, 2H, H4″ & H5′), 4.72 (m, 1H, H4′), 4.65 (dd, *J* = 12.2, 4.2 Hz, 1H, H5′), 4.32 – 4.20 (m, 4H, Fm-CH & Fm-CH$_2$), 4.19 – 4.09 (m, 3H, Fm-CH, H5″ & Fm-CH$_2$), 4.02 (m, 1H, H5″), 2.03 (s, 3H, OAc), 1.96 (s, 3H, OAc). **^{13}C NMR** (151 MHz, Chloroform-*d*) δ 169.9, 169.2, 166.1, 165.5, 165.5, 161.7, 149.9, 143.3, 143.2, 143.1, 141.5, 141.4, 138.5, 134.0, 133.9, 133.8, 130.0, 129.9, 129.8, 129.3, 128.9, 128.7, 128.6, 128.5, 127.9, 127.9, 127.3, 127.2, 125.4, 125.3, 125.3, 125.3, 120.1, 120.1, 120.0, 102.9 (C5), 89.9 (C1′), 84.3 (C1″), 80.7 (d, *J* = 7.5 Hz, C4″), 80.4 (C4′), 73.7 (C2′), 71.5 (C3′), 70.0 (C3″), 69.8 (C2″), 69.5 (dd, *J* = 10.2, 5.9 Hz, Fm-CH$_2$), 66.1 (d, *J* = 5.2 Hz, C4″), 63.8 (C4′), 48.0 (dd, *J* = 7.8, 4.6 Hz, Fm-CH), 20.6 (OAc), 20.4 (OAc). **HR-MS (ESI) m/z:** [M+H]$^+$ Calcd for $C_{67}H_{57}N_2O_{18}P$ 1209.3417, Found 1209.3419.

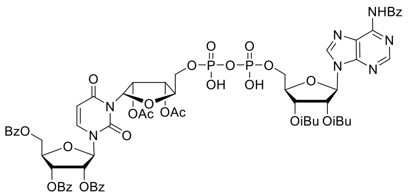

3-(2″,3″-di-*O*-acetyl-α-D-ribofuranosyl)phosphoryl-5″-*O*-(*N*6-benzoyl-9-(2‴,3‴-di-*O*-isobutyryl-β-D-ribofuranosyl)adenine)pyrophosphoryl-1-(2′,3′,5′-tri-*O*-benzoyl-β-D-ribofuranosyl)uracil (14α)

A solution of **13α** (121 mg, 0.1 mmol) and Et$_3$N (2 ml) in anhydrous MeCN (2 ml) was stirred for overnight at room temperature. The solution was concentrated under reduced pressure and the crude residue was co-evaporated with a mixture of anhydrous MeCN (3x). DCI (30 mg, 0.25 mmol) was added and the mixture was co-evaporated with anhydrous MeCN (3x) before adding anhydrous MeCN (2.5 ml). **7**(4) (142 mg, 0.2 mmol) was co-evaporated with anhydrous toluene (2x) before adding dropwise to the white suspension using anhydrous MeCN (1.5 ml). The reaction mixture was vigorously stirred under argon atmosphere for 15 min before adding *t*-BuOOH (5.5 M, 182 µl, 1 mmol) at 0 °C. After 30 min, Et$_3$N (3 ml) was added and the mixture was stirred for 4h. The solution was concentrated under reduced

pressure. Purification by size exclusion chromatography LH-20 (DCM/MeOH = 1/1) and flash C18 (H_2O/ACN = 95/5 – 0/100), then subsequent concentrated provided the title compound **14α** (110 mg, 78% over 4 steps) as a white foam. **Rf** = 0.3 (DCM/MeOH = 10/1).

U-β-ADPr using route A

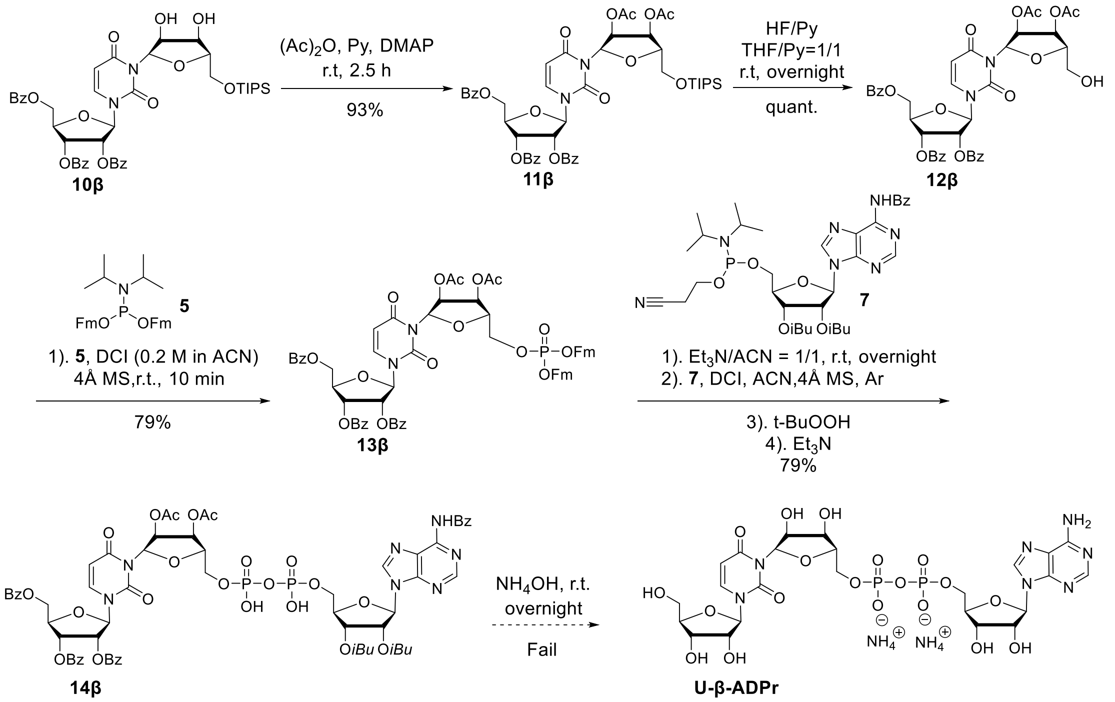

Scheme S2. Synthesis of U-β-ADPr by route A fail.

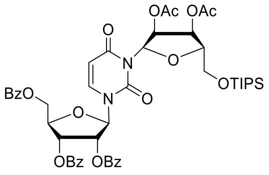

1-(2′,3′,5′-tri-*O*-benzoyl-β-D-ribofuranosyl)-3-[2″,3″-di-*O*-acetyl-5″-*O*-(triisopropylsilyl)-β-D-ribofuranosyl]-uracil (11β)

A mixture of **10β** (477 mg, 0.57 mmol), acetic anhydride (532 µL, 5.65 mmol) and DMAP (3.5 mg, 0.028 mmol) in pyridine (5 ml) was stirred at room temperature for overnight. The mixture was concentrated under reduced pressure. The crude material was purified by silica gel chromatography (PE/EA = 4/1 – 2/1 – 1/1) to give compound **11β** as white foam (493 mg, 0.53 mmol, 93%.). **Rf** = 0.6 (PE/EA = 2/1). **^{1}H NMR** (500 MHz, Chloroform-*d*) δ 8.12 – 8.06 (m, 2H, arom.), 7.96 (d, *J* = 8.3 Hz, 4H, arom.), 7.60 (d, *J* = 7.4 Hz, 1H, arom.), 7.59 – 7.53 (m, 2H, arom.), 7.51 – 7.44 (m, 2H, arom.), 7.41 – 7.34 (m, 5H, H6, arom.), 6.36 (d, *J* = 2.8 Hz, 1H, H1″), 6.33 (d, *J* = 5.7 Hz, 1H, H1′), 5.91 – 5.86 (m, 1H, H3′), 5.77 (dd,

J = 6.6, 2.9 Hz, 1H, H2″), 5.67 (t, J = 5.5 Hz, 1H, H2′), 5.63 (dd, J = 16.6, 7.7 Hz, 2H, H3″ & H5), 4.83 (dd, J = 11.9, 2.4 Hz, 1H, H5′), 4.73 – 4.63 (m, 2H, H5′ & H4′), 4.18 – 4.10 (m, 1H, H4″), 3.92 (m, 2H, H5″), 2.06 (s, 6H, OAc), 1.11 – 1.02 (m, 21H, TIPS). **^{13}C NMR** (151 MHz, Chloroform-*d*) δ 169.9, 169.6, 166.2, 165.5, 161.5, 138.1, 133.9, 133.9, 133.8, 130.1, 130.0, 129.8, 129.4, 128.9, 128.7, 128.7, 128.7, 128.5, 103.3 (C5), 88.4 (C1′), 86.7 (C1″), 82.2 (C4″), 80.8 (C4′), 74.0 (C2′), 73.3 (C2″), 71.4 (C3′), 71.3 (C2″), 64.1 (C5″), 63.9 (C5′), 20.8 (OAc), 20.6 (OAc), 18.0 (TIPS), 12.0 (TIPS). **HR-MS (ESI) m/z:** $[M+H]^+$ Calcd for $C_{48}H_{56}N_2O_{15}Si$ 929.3523, Found 929.3529.

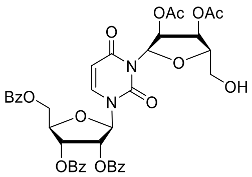

1-(2′,3′,5′-tri-*O*-benzoyl-β-D-ribofuranosyl)-3-[2″,3″-di-*O*-acetyl-β-D-ribofuranosyl]-uracil (12β)

Compound **11β** (493 mg, 0.53 mmol) was co-evaporated with toluene (3×), then hook up to oil pump to dryness. Then **11β** was dissolved in pyridine/THF (6 mL, 1/1 v/v) and HF·pyridine (900 μL) was added. The reaction was stirred for 10 h at room temperature. Then the reaction was quenched by aq. $NaHCO_3$ (sat.). The reaction mixture was extracted with EA, the combined organic layer was dried over Na_2SO_4, concentrated under reduced pressure and purified by silica gel chromatography (PE/EA = 2/1 – 1/1 – 1/2) to obtain **12β** (421 mg, 0.53 mmol, quant.). **Rf** = 0.3 (PE/EA = 1/1). **^{1}H NMR** (500 MHz, Chloroform-*d*) δ 8.10 (d, J = 9.8 Hz, 2H, arom.), 7.96 (t, J = 8.9 Hz, 4H, arom.), 7.63 – 7.54 (m, 3H, arom.), 7.49 (t, J = 7.8 Hz, 2H, arom.), 7.43 – 7.37 (m, 5H, H6, arom.), 6.46 (d, J = 5.1 Hz, 1H, H1″), 6.34 (d, J = 5.4 Hz, 1H, H1′), 5.91 – 5.87 (m, 1H, H3′), 5.86 – 5.82 (m, 1H, H2″), 5.71 (t, J = 5.7 Hz, 1H, H2′), 5.68 – 5.62 (m, 2H, H3″ & H5), 4.84 (dd, J = 12.2, 2.8 Hz, 1H, H5′), 4.75 – 4.70 (m, 1H, H4′), 4.67 (dd, J = 12.2, 3.8 Hz, 1H, H5′), 4.16 (m, 1H, H4″), 3.89 (d, J = 12.4 Hz, 1H, H5″), 3.75 (m, 1H, H5″), 3.20 (d, J = 8.7 Hz, 1H, H5″-OH), 2.10 (s, 3H, OAc), 2.02 (s, 3H, OAc). **^{13}C NMR** (151 MHz, Chloroform-*d*) δ 169.9, 169.8, 166.2, 165.5, 165.5, 162.0, 150.6, 138.4, 134.0, 133.9, 133.8, 130.1, 130.0, 129.8, 129.3, 128.9, 128.7, 128.7, 128.5 (arom.), 103.4 (C5), 88.5 (C1″), 85.9 (C1′), 83.4 (C4″), 80.8 (C4′), 74.0 (C2′), 71.8 (C2″), 71.1 (C3′), 70.7 (C3″), 63.7 (C5′), 62.2 (C5″), 20.7 (OAc), 20.6 (OAc). **HR-MS (ESI) m/z:** $[M+H]^+$ Calcd for $C_{39}H_{36}N_2O_{15}$ 773.2188, Found 773.2189.

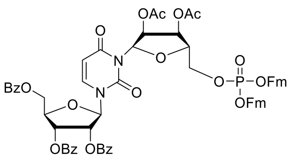

1-(2′,3′,5′-tri-*O*-benzoyl-β-ᴅ-ribofuranosyl)-3-[2″,3″-di-*O*-acetyl-5″-*O*-(di-flourenyl)-β-ᴅ-ribofuranosyl]-uracil (13β)

Compound **13β** (421 mg, 0.545 mmol), DCI activator (4,5-dicyanoimidozle, 129 mg) and freshly activated 4Å molecular sieves were added into flash. **5**(3) (0.2 M in ACN, 4.1 mL, 0.82 mmol) were added into the mixture and the reaction was stirred for 20 min at room temperature after which *t*-BuOOH (5.5 M in decane, 1 mL, 5.45 mmol) was added at 0 ºC. The reaction was stirred at same temperature for 30 minutes and quenched by aq. $NaHCO_3$ (sat.). The mixture was filtered and extracted with EtOAc (3×), washed with brine. The combined organic layer was dried over Na_2SO_4, concentrated under reduced pressure and purified by flash silica gel chromatography (PE/EA = 2/1 – 3/2 – 1/1 – 2/3- 1/2) to **13β** obtain (519 mg, 79%) as white foam. **Rf** = 0.3 (PE/EA = 1/1). **^{1}H NMR** (500 MHz, Chloroform-*d*) δ 8.09 – 8.05 (m, 2H, arom.), 7.93 (d, *J* = 8.4 Hz, 4H, arom.), 7.68 (t, *J* = 8.0 Hz, 4H, arom.), 7.61 – 7.46 (m, 9H, arom.), 7.38 – 7.27 (m, 9H, H6, arom.), 7.25 – 7.21 (m, 4H, arom.), 6.42 (s, 1H, H1″), 6.25 (s, 1H, H1′), 5.82 (d, *J* = 5.3 Hz, 1H, H3′), 5.70 (dd, *J* = 6.6, 2.5 Hz, 1H, H2″), 5.64 – 5.58 (m, 2H, H2′ & H3″), 5.51 (d, *J* = 8.1 Hz, 1H, H5), 4.73 (d, *J* = 13.1 Hz, 1H, H5′), 4.61 (dd, *J* = 12.4, 3.5 Hz, 1H, H5′), 4.56 (s, 1H, H4′), 4.25 (m, H4″, Fm-CH_2, Fm-CH & H5″), 4.14 (dd, *J* = 7.0, 3.6 Hz, 2H, H5″ & Fm-CH), 2.07 (s, 3H, OAc), 2.01 (s, 3H, OAc). **^{31}P NMR** (202 MHz, Chloroform-*d*) δ -1.7. **^{13}C NMR** (151 MHz, Chloroform-*d*) δ 169.6, 169.3, 166.0, 165.3, 165.3, 161.2, 143.2, 143.1, 143.1, 143.0, 141.3, 133.8, 133.8, 133.7, 130.0, 129.8, 129.6, 129.2, 128.8, 128.6, 128.6, 128.6, 128.3, 127.8, 127.1, 125.3, 125.3, 125.2, 125.2, 120.0, 119.9, 119.9, 103.1 (C5), 80.6 (C4′), 80.0 (d, *J* = 7.6 Hz, C4″), 73.8, 73.2 (C2″), 71.0 (C3′), 70.1, 69.5 (dd, *J* = 6.1, 4.1 Hz, C5″), 66.9 (d, *J* = 4.3 Hz, Fm-CH_2), 63.6 (C5′), 47.9 (dd, *J* = 7.7, 2.2 Hz, Fm-CH), 20.6 (OAc), 20.4 (OAc). **HR-MS (ESI) m/z:** $[M+H]^+$ Calcd for $C_{67}H_{57}N_2O_{18}P$ 1209.3417, Found 1209.3419.

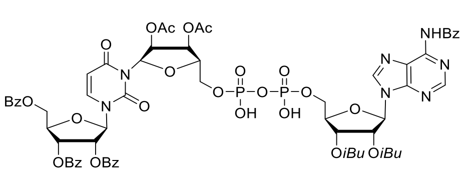

3-(2″,3″-di-*O*-acetyl-β-ᴅ-ribofuranosyl)phosphoryl-5″-*O*-(*N*6-benzoyl-9-(2‴,3‴-di-*O*-isobutyryl-β-ᴅ-ribofuranosyl)adenine)pyrophosphoryl-1-(2′,3′,5′-tri-*O*-benzoyl-β-ᴅ-ribofuranosyl)uracil (14β)

A solution of **13β** (121 mg, 0.1 mmol) and Et_3N (2 ml) in anhydrous MeCN (2 ml) was stirred for overnight at room temperature. The solution was concentrated under reduced pressure and the crude residue was co-evaporated with a mixture of anhydrous MeCN (3x). DCI (30 mg, 0.25 mmol) was added and the mixture was co-evaporated with anhydrous MeCN (3x) before adding anhydrous MeCN (1.5 ml). **7**(4) (142 mg, 0.2 mmol) was co-evaporated with anhydrous toluene (2x) before adding dropwise to the white suspension using anhydrous MeCN (1.5 ml). The reaction mixture was vigorously stirred under argon atmosphere for 15 min before adding *t*-BuOOH (5.5 M, 182 μl, 1 mmol) at 0 °C. After 30 min, Et_3N (3 ml) was added and the mixture was stirred for 4h. The solution was concentrated under reduced pressure. Purification by size exclusion chromatography LH-20 (DCM/MeOH = 1/1) and flash C18 (H_2O/ACN = 95/5 – 0/100), then subsequent concentrated provided the title compound **14β** (112 mg, 79% over 4 steps) as a white foam. **Rf** = 0.3 (DCM/MeOH = 10/1).

U-α-ADPr using route B

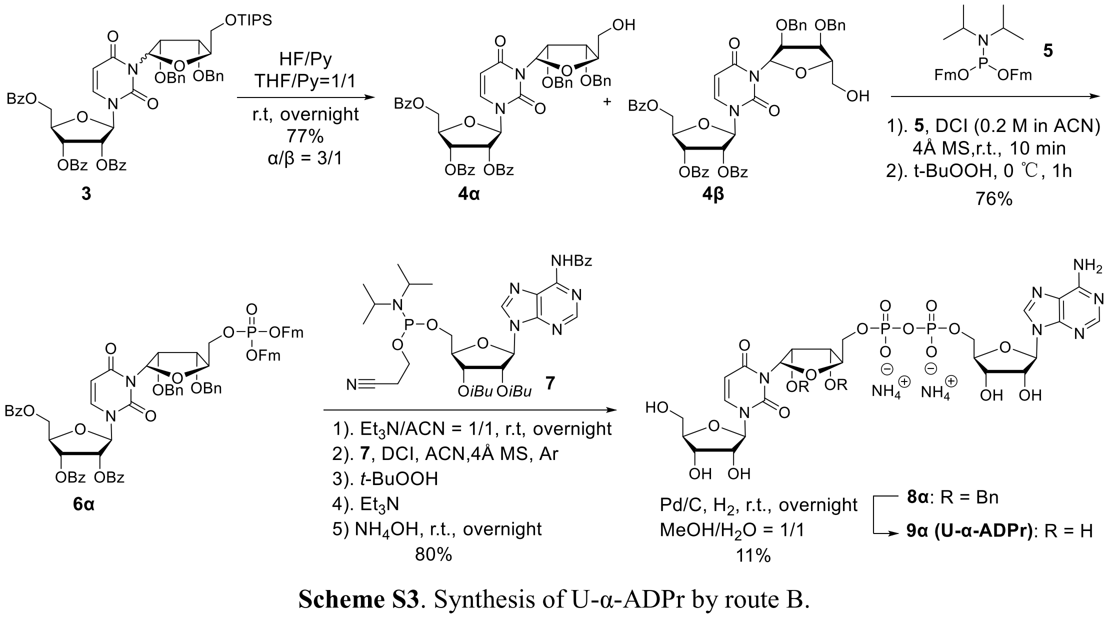

Scheme S3. Synthesis of U-α-ADPr by route B.

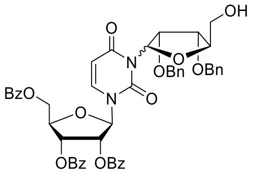

1-(2′,3′,5′-di-*O*-benzoyl-β-D-ribofuranosyl)-3-[2″,3″-di-*O*-benzyl-α/β-D-ribofuranosyl]-uracil (4)

Compound **3** (2.92 g, 2.84 mmol) was co-evaporated with toluene (3×), then hook up to oil pump to

dryness. Then compound **3** was dissolved in pyridine/THF (10 mL, 1/1 v/v) and HF·pyridine (1.5 mL) was added. The reaction was stirred for overnight at room temperature. Then the reaction was quenched by aq. $NaHCO_3$ (sat.). The reaction mixture was extracted with DCM. The combined organic layer was dried over Na_2SO_4, concentrated under reduced pressure and purified by flash silica gel chromatography (PE/EA = 4/1- 2/1 -1/1 - 1/2) to obtain compound **4** as white foam (α isomer: 1.16 g, β isomer: 730 mg, yield = 77%). **Rf** (α isomer) = 0.3 (PE/EA = 1/1). **Rf** (β isomer) = 0.5 (PE/EA = 1/1). β isomer: **^{1}H NMR** (500 MHz, Chloroform-*d*) δ 8.10 (d, *J* = 6.7 Hz, 2H, arom.), 8.00 (d, *J* = 6.7 Hz, 2H, arom.), 7.90 (d, *J* = 8.0 Hz, 2H, arom.), 7.64 – 7.57 (m, 2H, arom.), 7.56 – 7.48 (m, 3H, arom.), 7.44 – 7.38 (m, 3H, H6, arom.), 7.35 (t, *J* = 7.7 Hz, 2H, arom.), 7.32 – 7.30 (m, 3H, arom.), 7.27 (m, 6H, arom.), 7.22 (m, 1H, arom.), 6.51 (d, *J* = 4.0 Hz, 1H, H1′′), 6.28 (d, *J* = 5.2 Hz, 1H, H1′), 5.86 (t, *J* = 5.4 Hz, 1H, H3′), 5.70 (t, *J* = 5.6 Hz, 1H, H2′), 5.59 (d, *J* = 8.2 Hz, 1H, H5), 4.84 (dd, *J* = 12.3, 2.8 Hz, 1H, H5′), 4.73 (m, 1H, H4′), 4.68 (dd, *J* = 12.3, 3.7 Hz, 1H, H5′), 4.59 (d, *J* = 11.4 Hz, 1H, Bn-CH_2), 4.54 (s, 2H, Bn-CH_2), 4.49 – 4.45 (m, 1H, H2′′), 4.44 – 4.40 (m, 2H, H3′′ & Bn-CH_2), 4.16 – 4.11 (m, 1H, H4′′), 3.86 (d, *J* = 12.4 Hz, 1H, H5′′), 3.67 – 3.60 (m, 1H, H5′′), 2.94 (d, *J* = 7.5 Hz, 1H, 5′′-OH). **^{13}C NMR** (151 MHz, Chloroform-*d*) δ 166.1, 165.4, 165.4, 161.9, 150.5, 138.1, 138.0, 137.7, 134.0, 134.0, 133.9, 130.0, 130.0, 129.8, 129.3, 128.9, 128.7, 128.7, 128.7, 128.5, 128.5, 128.2, 128.0, 128.0, 127.9, 103.4 (C5), 88.8 (C1′), 87.6 (C1′′), 83.5 (C4′′), 80.7 (C4′), 78.3 (C2′′), 76.1 (C3′′), 74.1 (C2′), 72.8 (Bn-CH_2), 72.6 (Bn-CH_2), 71.1 (C3′), 63.6 (C5′), 62.3 (C5′′). **HR-MS (ESI) m/z:** $[M+H]^+$ Calcd for $C_{49}H_{44}N_2O_{13}$ 869.2916, Found 869.2913. α isomer: **^{1}H NMR** (500 MHz, Chloroform-*d*) δ 8.10 – 8.07 (m, 2H, arom.), 7.97 – 7.90 (m, 4H, arom.), 7.59 – 7.49 (m, 3H, arom.), 7.45 (t, *J* = 7.8 Hz, 2H, arom.), 7.39 – 7.26 (m, 11H, H6, arom.), 7.21 – 7.17 (m, 2H, arom.), 7.16 – 7.12 (m, 2H, arom.), 6.58 (d, *J* = 5.6 Hz, 1H, H1′′), 6.36 (d, *J* = 5.1 Hz, 1H, H1′), 5.95 – 5.81 (m, 1H, H3′), 5.73 (t, *J* = 5.6 Hz, 1H, H2′), 5.50 (d, *J* = 8.1 Hz, 1H, H5), 4.82 (dd, *J* = 11.7, 2.4 Hz, 1H, H5′), 4.75 – 4.71 (m, 1H, H4′′), 4.70 – 4.64 (m, 2H, H4′ & H5′), 4.60 (d, *J* = 12.0 Hz, 1H, Bn-CH_2), 4.54 (d, *J* = 11.9 Hz, 1H, Bn-CH_2), 4.48 (d, *J* = 12.0 Hz, 1H, Bn-CH_2), 4.33 (d, *J* = 11.9 Hz, 1H, Bn-CH_2), 4.17 (t, *J* = 5.9 Hz, 1H, H2′′), 4.02 (dd, *J* = 9.1, 6.0 Hz, 1H, H3′′), 3.80 (dd, *J* = 12.5, 2.5 Hz, 1H, H5′′), 3.53 (dd, *J* = 12.5, 3.1 Hz, 1H, H5′′). **^{13}C NMR** (151 MHz, Chloroform-*d*) δ 166.0, 165.2, 165.2, 162.4, 149.4, 137.9, 137.8, 137.6, 133.7, 133.6, 133.6, 129.9, 129.8, 129.6, 129.2, 128.7, 128.6, 128.6, 128.5, 128.4, 128.4, 128.3, 127.8, 127.8, 127.8, 127.6 (arom.), 102.3 (C5), 88.4 (C1′), 86.0 (C1′′), 81.6 (C4′′), 80.3 (C4′), 77.0 (C3′′), 77.0 (C2′′), 74.4 (Bn-CH_2), 73.9 (C2′), 72.7 (Bn-CH_2), 71.1 (C3′), 63.7 (C5′), 61.1 (C5′′). **HR-MS (ESI) m/z:** $[M+H]^+$ Calcd for $C_{49}H_{44}N_2O_{13}$ 869.2916,

Found 869.2918.

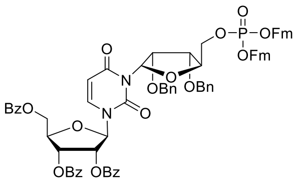

1-(2′,3′,5′-tri-*O*-benzoyl-β-ᴅ-ribofuranosyl)-3-[2″,3″-di-*O*-benzyl-5″-*O*-(di-flourenyl)-α-ᴅ-ribofuranosyl]-uracil (6α)

Compound **4α** (1.16 g, 1.34 mmol), DCI activator (4,5-dicyanoimidozle, 317 mg) and freshly activated 4Å molecular sieves were added into flash. **5**(3) (0.2 M in ACN, 10 mL, 2.01 mmol) were added into the mixture and the reaction was stirred for 30 min at room temperature after which *t*-BuOOH (5.5 M in decane, 2.4 mL, 13.4 mmol) was added at 0 ºC. The reaction was stirred at same temperature for 30 minutes and quenched by aq. $NaHCO_3$ (sat.). The mixture was filtered and extracted with EtOAc (3×), washed with brine. The combined organic layer was dried over Na_2SO_4, concentrated under reduced pressure and purified by flash silica gel chromatography (PE/EA = 3/1- 2/1 -1/1 - 1/2) and LH-20 (DCM/MeOH = 1/1) to obtain **6α** (1.336 g, 1.02 mmol, 76%) as white foam. **^{31}P NMR** (202 MHz, Chloroform-*d*) δ -1.7. **^{1}H NMR** (500 MHz, Chloroform-*d*) δ 8.09 (d, *J* = 7.8 Hz, 2H, arom.), 7.95 (d, *J* = 7.9 Hz, 2H, arom.), 7.87 (d, *J* = 7.8 Hz, 2H, arom.), 7.69 (t, *J* = 7.5 Hz, 4H, arom.), 7.62 – 7.55 (m, 2H, arom.), 7.46 (m, 7H, arom.), 7.40 – 7.26 (m, 10H, H6), 7.23 – 7.10 (m, 13H, arom.), 6.49 (d, *J* = 5.7 Hz, 1H, H1″), 6.34 (d, *J* = 5.3 Hz, 1H, H1′), 5.85 (t, *J* = 5.3 Hz, 1H, H3′), 5.67 (t, *J* = 5.6 Hz, 1H, H2′), 5.49 (d, *J* = 8.2 Hz, 1H, H5), 4.83 (dd, *J* = 12.0, 2.5 Hz, 1H, H5′), 4.76 (d, *J* = 9.5 Hz, 1H, H4′), 4.70 – 4.67 (m, 1H, H4′), 4.64 (dd, *J* = 12.0, 4.0 Hz, 1H, H5′), 4.49 (d, *J* = 12.0 Hz, 2H, Bn-CH_2), 4.32 (dd, *J* = 15.6, 12.3 Hz, 2H, Bn-CH_2), 4.24 – 4.12 (m, 5H, H5″, Fm-CH & Fm-CH_2), 4.10 – 4.03 (m, 3H, Fm-CH , Fm-CH_2 & H2″), 3.90 (m, 1H, H5″), 3.82 (dd, *J* = 9.1, 6.3 Hz, 1H, H3″). **^{13}C NMR** (151 MHz, Chloroform-*d*) δ 166.2, 165.4, 165.3, 161.9, 149.7, 143.3, 143.2, 141.5, 141.4, 137.7, 137.5, 133.9, 133.8, 133.8, 130.0, 130.0, 129.8, 129.4, 128.9, 128.8, 128.7, 128.6, 128.5, 128.5, 128.0, 128.0, 127.9, 127.9, 127.9, 127.9, 127.2, 127.2, 127.2, 127.2, 125.4, 125.3, 125.3, 120.1, 120.1, 120.0, 102.3 (C5), 88.2 (C1′), 86.1 (C1″), 80.4 (C4′), 79.8 (d, *J* = 7.3 Hz, C4″), 76.7 (C3″), 76.5 (C2″), 74.5 (Bn-CH_2), 73.9 (C2′), 72.8 (Bn-CH_2), 71.2 (C3′), 69.4 (dd, *J* = 17.7, 5.9 Hz, Fm-CH_2), 65.7 (d, *J* = 5.3 Hz, C5″), 63.8 (C5′), 48.0 (dd, *J* = 7.9, 1.7 Hz , Fm-CH). **HR-MS (ESI) m/z:** $[M+H]^+$ Calcd for $C_{77}H_{65}N_2O_{16}P$ 1305.4145, Found 1305.4147.

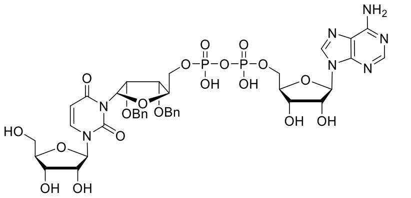

3-(2″,3″-di-*O*-benzyl-α-D-ribofuranosyl)phosphoryl-5″-*O*-(9-β-D-ribofuranosyl adenine)pyrophosphoryl-1-(β-D-ribofuranosyl)uracil (8α)

A solution of **6α** (261 mg, 0.2 mmol) and Et_3N (2.5 ml) in anhydrous MeCN (2.5 ml) was stirred for overnight at room temperature. The solution was concentrated under reduced pressure and the crude residue was co-evaporated with a mixture of anhydrous MeCN (3x). DCI (59 mg, 0.5 mmol) was added and the mixture was co-evaporated with anhydrous MeCN (3x) before adding anhydrous MeCN (2.5 ml). **7**(4) (284 mg, 0.4 mmol) was co-evaporated with anhydrous toluene (2x) before adding dropwise to the white suspension using anhydrous MeCN (2.5 ml). The reaction mixture was vigorously stirred under argon atmosphere for 15 min before adding *t*-BuOOH (5.5 M, 360 μl, 2 mmol) at 0 °C. After 30 min, Et_3N (2.5 ml) was added and the mixture was stirred for 4h. The solution was concentrated under reduced pressure. Purification by size exclusion chromatography LH-20 (DCM/MeOH = 1/1) and subsequent concentrated provided the intermediate. The intermediate was dissolved in NH_4OH (20 ml). The reaction mixture was stirred overnight at room temperature. The reaction was monitored by LC-MS. Upon completion, the solution was concentrated under reduced pressure. Purification by flash C18 and subsequent concentrated provided the NH_4 salt of title compound **8α** (159 mg, 0.16 mmol, 80% over five steps) as a white foam. **Rf** = 0.4 ($EtOH/NH_4OH/H_2O$= 10/2/1). **^{1}H NMR** (500 MHz, Chloroform-*d*) δ 8.45 (s, 1H, HA8), 8.06 (s, 1H, HA2), 7.90 (d, *J* = 8.0 Hz, 1H, H6), 7.24 (s, 5H, Bn), 7.18 – 7.11 (m, 3H, Bn), 7.03 – 6.93 (m, 2H, Bn), 6.45 (d, *J* = 4.9 Hz, 1H, H1″), 5.96 (s, 1H), 5.82 (s, 1H), 5.45 (d, *J* = 7.9 Hz, 1H, H5), 4.37 – 4.10 (m, 14H), 4.02 (s, 1H), 3.91 (s, 1H), 3.77 (m, 3H). **^{31}P NMR** (202 MHz, Chloroform-*d*) δ -6.6. **HR-MS (ESI) m/z:** $[M-H]^-$ Calcd for $C_{38}H_{45}N_7O_{19}P_2$ 941.2173, Found 941.2172.

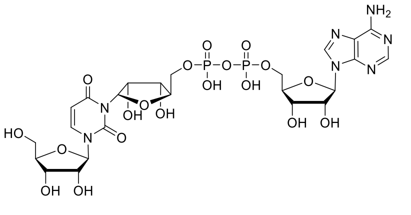

3-(α-ADPr)-uracil (U-α-ADPr)

A mixture of **8α** (90 mg, 0.093 mmol) and 90 mg of 10% Pd/C in a 3:2 solution of $MeOH/H_2O$ (10 ml) was hydrogenated at room temperature and atmospheric pressure during 11 h. The mixture was filtered

through a millipore filter and the filtrate was concentrated under reduced pressure. The crude material was purified twice by flash C18 (MeOH/H_2O = 0%-100%), and subsequent purified by P2 (H_2O), lyophilization to give compound **U-α-ADPr** as white foam (8 mg, 0.010 mmol, 11%). **^{1}H NMR** (500 MHz, Deuterium Oxide) δ 8.56 (s, 1H, HA8), 8.27 (s, 1H, HA2), 7.80 (d, *J* = 8.1 Hz, 1H, H6), 6.45 (d, *J* = 6.6 Hz, 1H, H1″), 6.12 (d, *J* = 5.6 Hz, 1H, H1‴), 5.79 (d, *J* = 3.5 Hz, 1H, H1′), 5.75 (d, *J* = 8.1 Hz, 1H, H5), 4.70 (t, *J* = 5.3 Hz, 1H, H2‴), 4.56 (d, *J* = 6.6 Hz, 1H, H3‴), 4.53 (d, *J* = 4.4 Hz, 1H, H2″), 4.44 (s, 1H, H4″), 4.39 (s, 1H, H4‴), 4.29 (dd, *J* = 21.6, 4.8 Hz, 4H, H3″, H2′&H5‴), 4.19 – 4.10 (m, 3H, H3′, H5″&H4′), 4.09 – 4.02 (m, 1H, H5″), 3.95 (d, *J* = 14.8 Hz, 1H, H5′), 3.81 (dd, *J* = 12.9, 4.3 Hz, 1H, H5′). **^{31}P NMR** (202 MHz, Deuterium Oxide) δ -11.2. **^{13}C NMR** (151 MHz, Deuterium Oxide) δ 164.8 (C4), 153.6 (CA6), 151.0 (C2), 150.1 (CA2), 148.8 (CA4), 140.9 (CA8), 139.8 (C6), 101.6 (C5), 90.3 (C1′), 87.3 (C1‴), 86.0 (C1″), 83.9 (d, *J* = 8.5 Hz, C4‴), 83.8 (C4′), 83.6 (d, *J* = 8.0 Hz, C4″), 74.7 (C2‴), 73.9 (C2′), 70.7 (C3‴), 70.7 (C2″), 70.3 (C3″), 69.0 (C3′), 65.8 (d, *J* = 3.3 Hz, C5″), 65.1 (d, *J* = 3.9 Hz, C5‴), 60.4 (C5′). **HR-MS (ESI) m/z:** $[M-H]^-$ Calcd for $C_{24}H_{33}N_7O_{19}P_2$ 784.1234, Found 784.1238. **HR-MS (ESI) m/z:** $[M+H]^+$ Calcd for $C_{24}H_{33}N_7O_{19}P_2$ 786.1379, Found 786.1376.

U-β-ADPr using route B

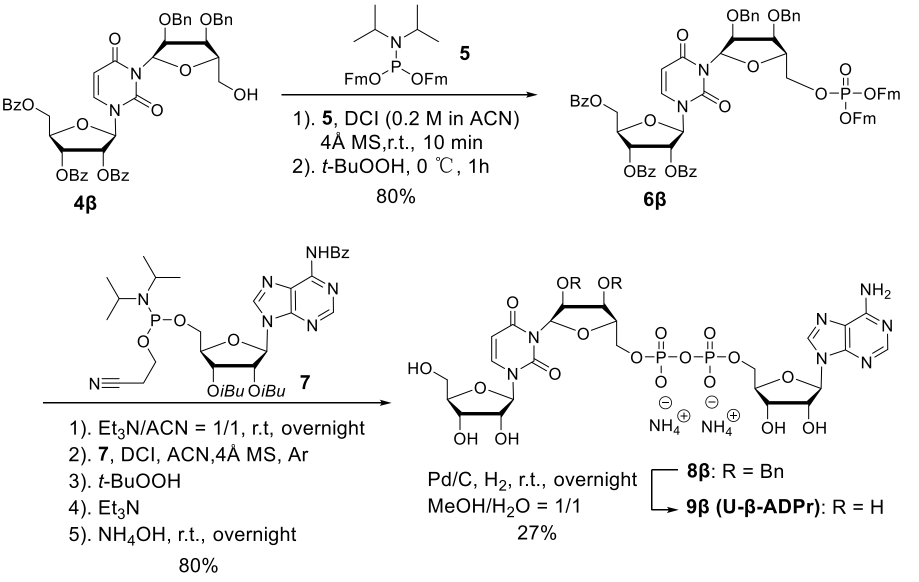

Scheme S4. Synthesis of U-β-ADPr by route B.

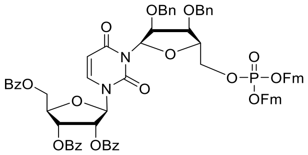

1-(2′,3′,5′-tri-*O*-benzoyl-β-D-ribofuranosyl)-3-[2″,3″-di-*O*-benzyl-5″-*O*-(di-flourenyl)-β-D-

ribofuranosyl]-uracil (6β)

Compound **4β** (730 mg, 0.84 mmol), DCI activator (4,5-dicyanoimidozle, 198 mg) and freshly activated 4Å molecular sieves were added into flash. **5** (3) (0.2 M in ACN, 6.3 mL, 1.26 mmol) were added into the mixture and the reaction was stirred for 30 min at room temperature after which *t*-BuOOH (5.5 M in decane, 1.53 mL, 8.4 mmol) was added at 0 ºC. The reaction was stirred at same temperature for 30 minutes and quenched by aq. $NaHCO_3$ (sat.). The mixture was filtered and extracted with EtOAc (3×), washed with brine. The combined organic layer was dried over Na_2SO_4, concentrated under reduced pressure and purified by flash silica gel chromatography (PE/EA = 3/1- 2/1 -1/1 - 1/2) and LH-20 (DCM/MeOH = 1/1) to obtain **6β** (872 g, 0.668 mmol, 80%) as white foam. **^{31}P NMR** (202 MHz, Chloroform-*d*) δ -1.6. **^{1}H NMR** (500 MHz, Chloroform-*d*) δ 8.10 – 8.06 (m, 2H, arom.), 7.98 – 7.95 (m, 2H, arom.), 7.85 (d, *J* = 7.7 Hz, 2H, arom.), 7.66 (t, *J* = 7.8 Hz, 4H, arom.), 7.62 – 7.57 (m, 2H, arom.), 7.53 – 7.45 (m, 8H, arom.), 7.40 (t, *J* = 7.7 Hz, 2H, arom.), 7.33 – 7.27 (m, 10H, arom.), 7.24 – 7.18 (m, 10H, H6, arom.), 6.47 (s, 1H, H1″), 6.10 (d, *J* = 5.3 Hz, 1H, H1′), 5.78 (s, 1H, H3′), 5.62 (s, 1H, H2′), 5.47 – 5.39 (m, 1H, H5), 4.74 (d, *J* = 10.6 Hz, 1H, H5′), 4.64 – 4.56 (m, 3H, H4′, H5′ & Bn-CH_2), 4.50 – 4.42 (m, 2H, Bn-CH_2), 4.30 – 4.16 (m, 10H, Bn-CH_2, H4″, H2″, H3″, H5″, Fm-CH_2 & Fm-CH), 4.10 (t, *J* = 7.2 Hz, 2H, Fm-CH & H5″). **^{13}C NMR** (151 MHz, Chloroform-*d*) δ 166.1, 165.4, 165.3, 161.4, 149.9, 143.3, 143.3, 143.2, 141.4, 141.4, 141.4, 141.4, 137.7, 137.5, 134.0, 133.9, 133.9, 130.0, 130.0, 129.8, 129.3, 128.9, 128.7, 128.7, 128.7, 128.6, 128.6, 128.5, 128.4, 128.1, 128.1, 127.9, 127.9, 127.9, 127.8, 127.2, 127.2, 127.2, 125.4, 125.4, 125.4, 125.3, 120.0, 120.0, 103.0 (C5), 88.0 (C1″), 80.6 (d, *J* = 8.1 Hz, C4″), 80.5 (C4′), 78.1, 74.1 (C2′), 72.7 (Bn-CH_2), 72.5 (Bn-CH_2), 71.1 (C3′), 69.5 (dd, *J* = 14.1, 6.1 Hz, Fm-CH_2), 67.6 (C5″), 63.6 (C5′), 48.0 (dd, *J* = 7.6, 4.2 Hz, Fm-CH). **HR-MS (ESI) m/z:** $[M+H]^+$ Calcd for $C_{77}H_{65}N_2O_{16}P$ 1305.4145, Found 1305.4146.

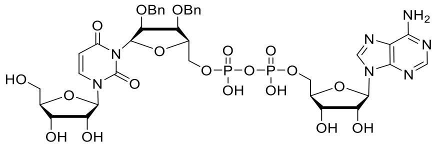

3-(2″,3″-di-*O*-benzyl-β-D-ribofuranosyl)phosphoryl-5″-*O*-(9-β-D-ribofuranosyl adenine)pyrophosphoryl-1-(β-D-ribofuranosyl)uracil (8β)

A solution of **6β** (131 mg, 0.1 mmol) and Et_3N (2 ml) in anhydrous MeCN (2 ml) was stirred for overnight at room temperature. The solution was concentrated under reduced pressure and the crude residue was co-evaporated with a mixture of anhydrous MeCN (3x). DCI (30 mg, 0.25 mmol) was added and the

mixture was co-evaporated with anhydrous MeCN (3x) before adding anhydrous MeCN (1.5 ml). **7** (4) (284 mg, 0.4 mmol) was co-evaporated with anhydrous toluene (2x) before adding dropwise to the white suspension using anhydrous MeCN (1.5 ml). The reaction mixture was vigorously stirred under argon atmosphere for 15 min before adding *t*-BuOOH (5.5 M, 182 µl, 1 mmol) at 0 °C. After 30 min, Et_3N (3 ml) was added and the mixture was stirred for 4h. The solution was concentrated under reduced pressure. Purification by size exclusion chromatography LH-20 (DCM/MeOH = 1/1) and subsequent concentrated provided the intermediate as a white foam. Then the intermediate was dissolved in NH_4OH (20 ml). The reaction mixture was stirred overnight at room temperature. The reaction was monitored by LC-MS. Upon completion, the solution was concentrated under reduced pressure. Purification by size exclusion chromatography LH-20 (DCM/MeOH = 1/1) and subsequent concentrated provided the NH_4 salt of title compound **8β** (77 mg, 0.08 mmol, 80% over five steps) as a white foam. **Rf** = 0.4 ($EtOH/NH_4OH/H_2O$= 10/2/1).

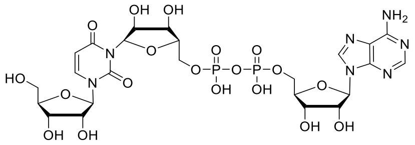

3-(β-ADPr)-uracil (U-β-ADPr)

A mixture of **8β** (136 mg, 0.141 mmol) and 100 mg of 10% Pd/C in a 1:1:2 solution of $MeOH/H_2O$/dioxane (20 ml) was hydrogenated at room temperature and atmospheric pressure during 17 h. The mixture was filtered through a millipore filter and the filtrate was concentrated under reduced pressure. The crude material was purified by HW-40 (0.1 M NH_4HCO_3 in water) and purified again by flash C18 (ACN/H_2O = 0%-100%), treated with Na^+- resin, lyophilization to give compound **U-β-ADPr** as white foam (30 mg, 0.038 mmol, 27%). **^{1}H NMR** (500 MHz, Deuterium Oxide) δ 8.35 (s, 1H, HA8), 8.13 (s, 1H, HA3), 7.63 (d, *J* = 8.3 Hz, 1H, H6), 6.07 (d, *J* = 3.0 Hz, 1H, H1″), 6.01 (d, *J* = 5.4 Hz, 1H, H1‴), 5.70 (d, *J* = 3.8 Hz, 1H, H1′), 5.58 (d, *J* = 8.2 Hz, 1H, H5), 4.61 – 4.53 (m, 2H, H2‴), 4.43 (t, *J* = 4.3 Hz, 1H), 4.35 (t, *J* = 6.7 Hz, 1H), 4.28 (s, 1H), 4.15 (s, 4H), 4.08 (t, *J* = 5.7 Hz, 1H), 4.04 – 3.94 (m, 3H), 3.82 (dd, *J* = 12.8, 2.8 Hz, 1H), 3.70 (dd, *J* = 12.8, 4.3 Hz, 1H). **^{31}P NMR** (202 MHz, Deuterium Oxide) δ -11.15. **^{13}C NMR** (126 MHz, Deuterium Oxide) δ 163.9 (C4), 160.6 (CA6), 155.5 (CA1), 152.7 (CA3), 150.6 (C2), 149.3 (CA5), 140.0 (C6), 139.8 (CA8), 101.4 (C5), 90.1 (C1′), 88.3 (C1″), 87.2 (C1‴), 83.9 (C4′), 83.7 (d, *J* = 8.9 Hz, C4‴), 81.8 (d, *J* = 8.3 Hz, C4″), 74.6 (C2‴), 73.8 (C2′), 71.4 (C2″), 70.2 (C3‴), 69.6 (C3″), 69.1 (C3′), 65.9 (d, *J* = 4.7 Hz, C5″), 65.1 (d, *J* = 4.2 Hz, C5‴), 60.5 (C5′). **HR-MS**

(ESI) m/z: [M-H]$^-$ Calcd for $C_{24}H_{33}N_7O_{19}P_2$ 784.1234, Found 784.1240. **HR-MS (ESI) m/z:** [M+H]$^+$ Calcd for $C_{24}H_{33}N_7O_{19}P_2$ 786.1379, Found 786.1376.

Copies of spectrum

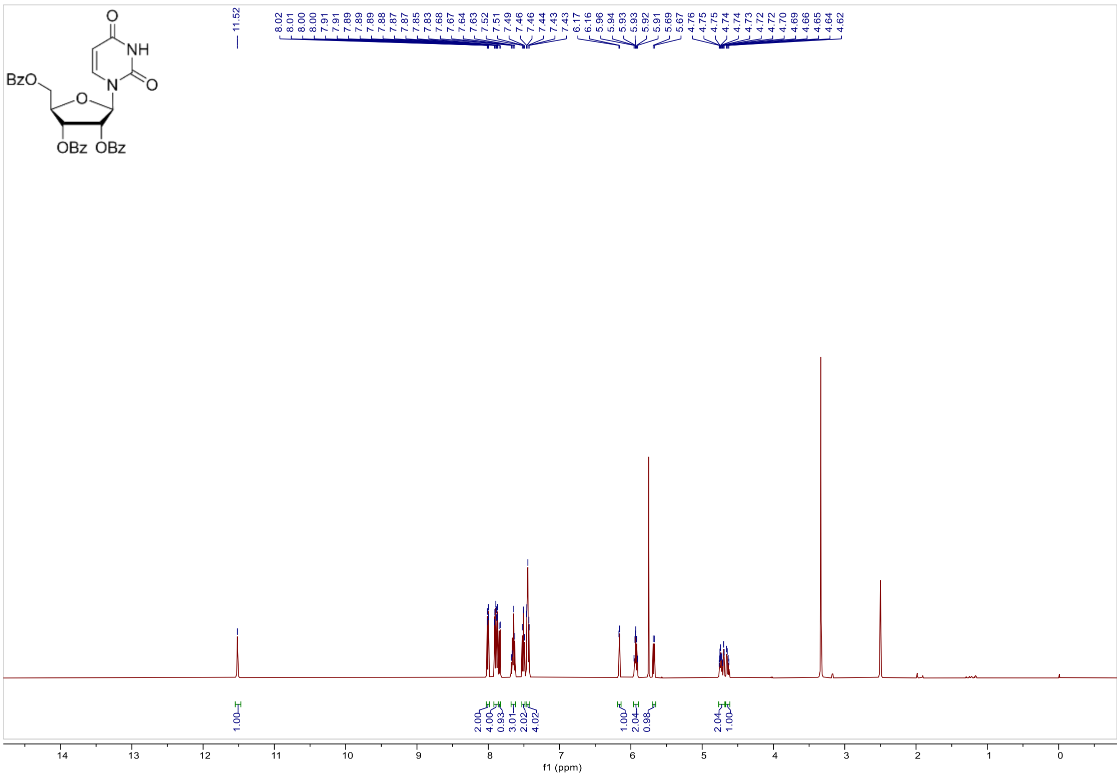

Figure S1. ^1H NMR spectrum of compound **1** in $CDCl_3$

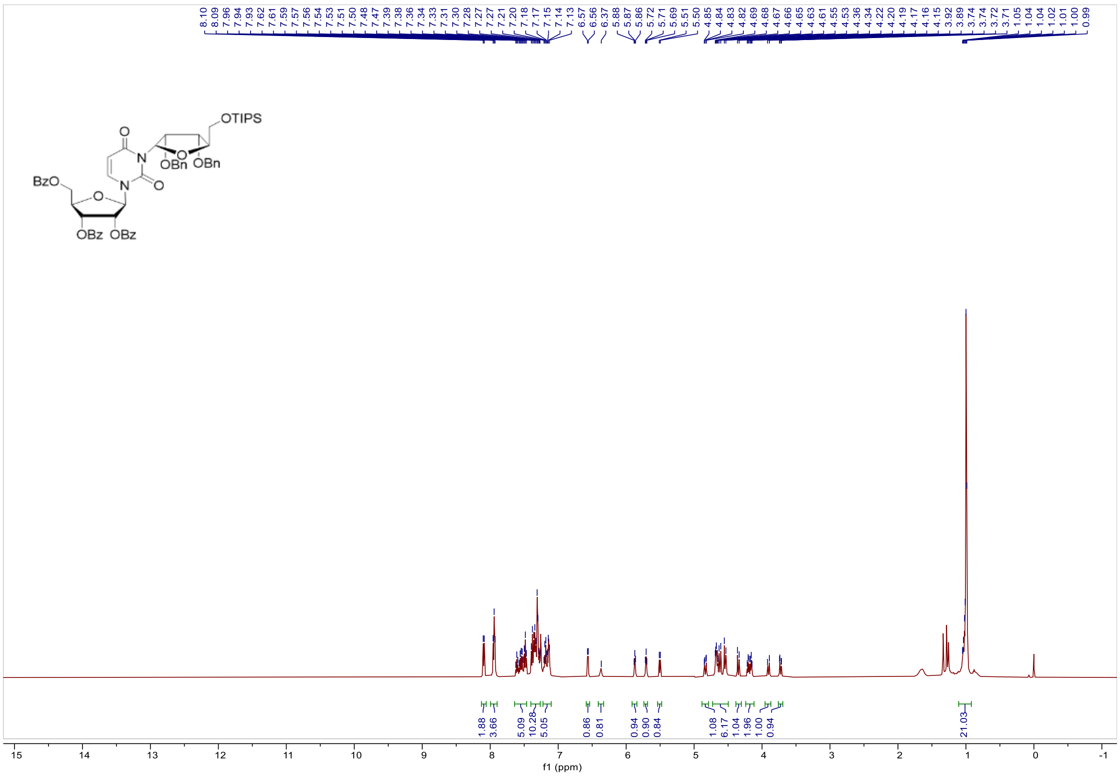

Figure S2. ^{1}H NMR spectrum of compound **3α** in $CDCl_3$

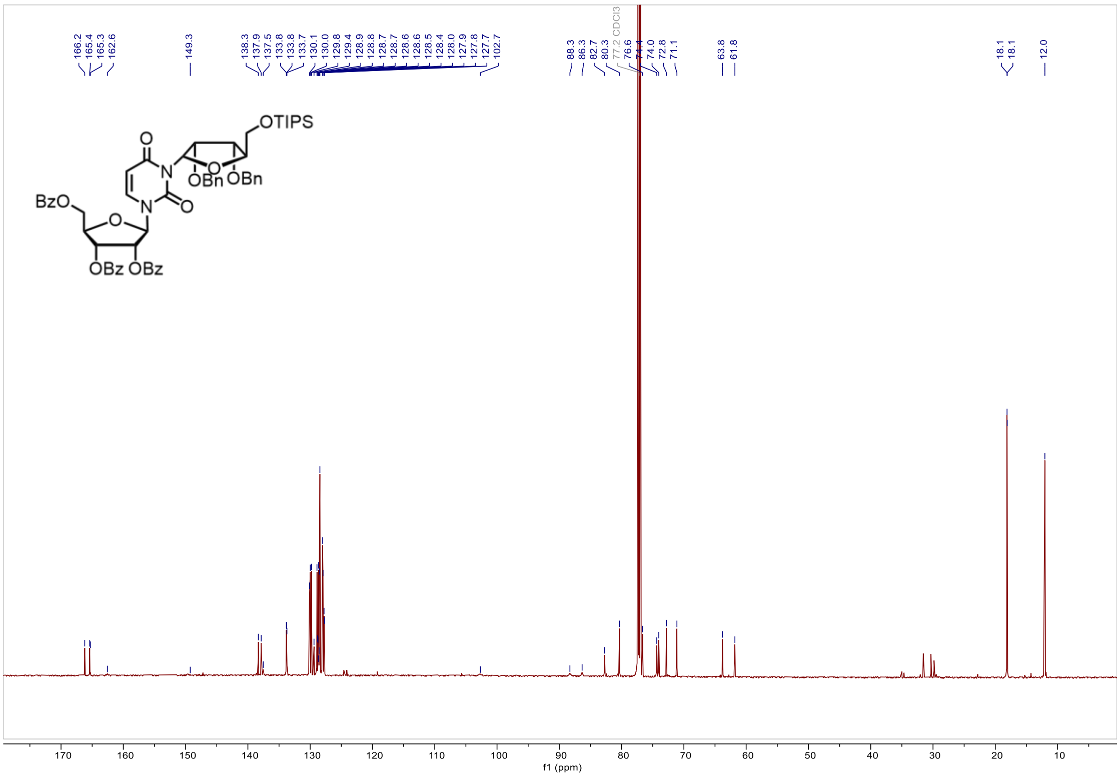

Figure S3. ^{13}C NMR spectrum of compound **3α** in $CDCl_3$

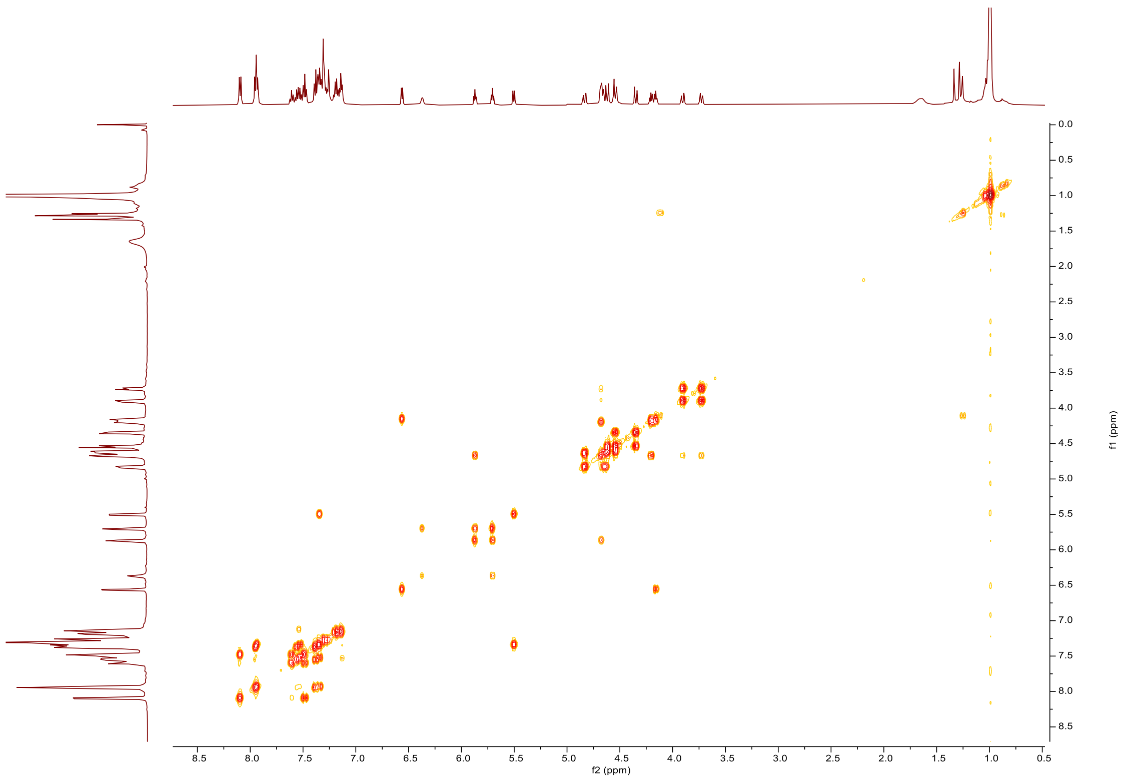

Figure S4. ^{1}H-^{1}H COSY spectrum of compound **3α** in $CDCl_3$

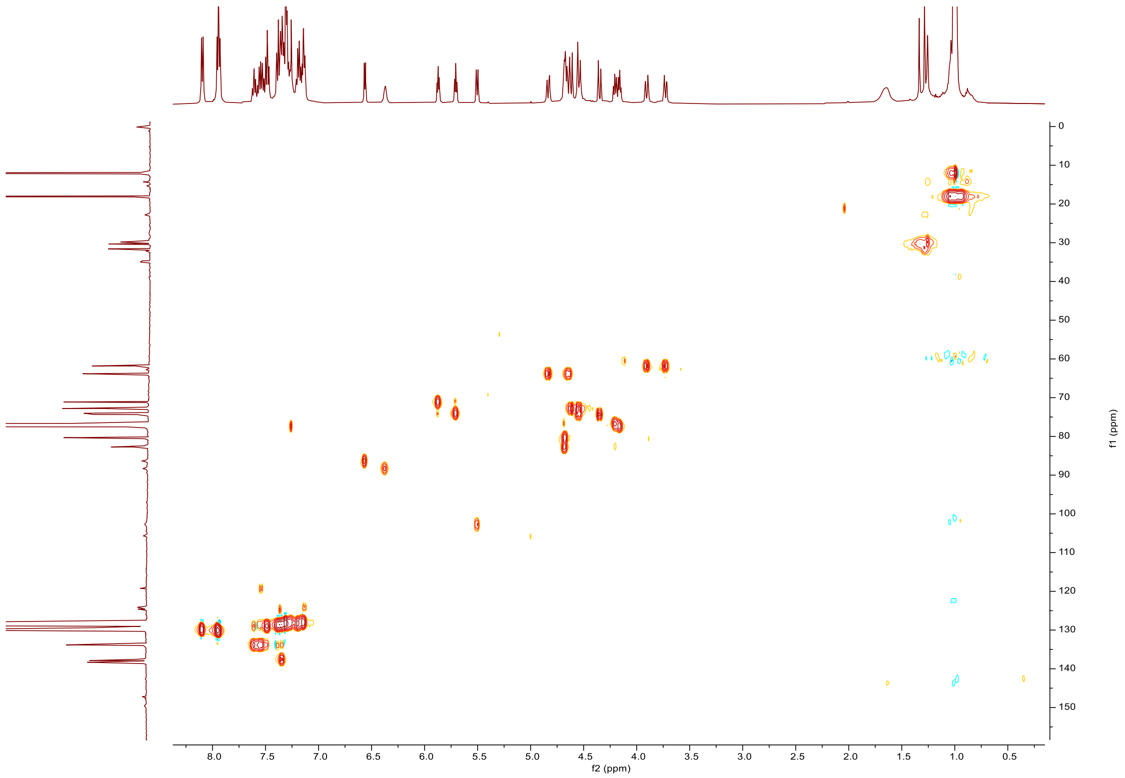

Figure S5. HSQC spectrum of compound **3α** in $CDCl_3$

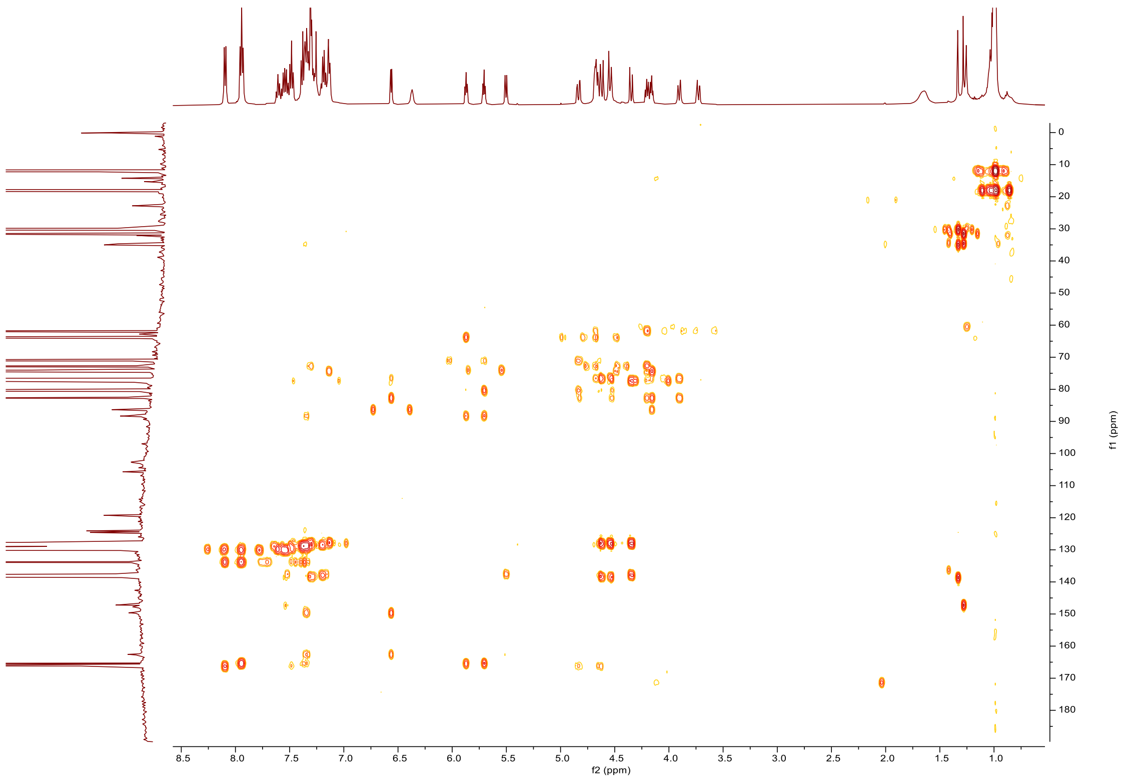

Figure S6. HMBC spectrum of compound **3α** in $CDCl_3$

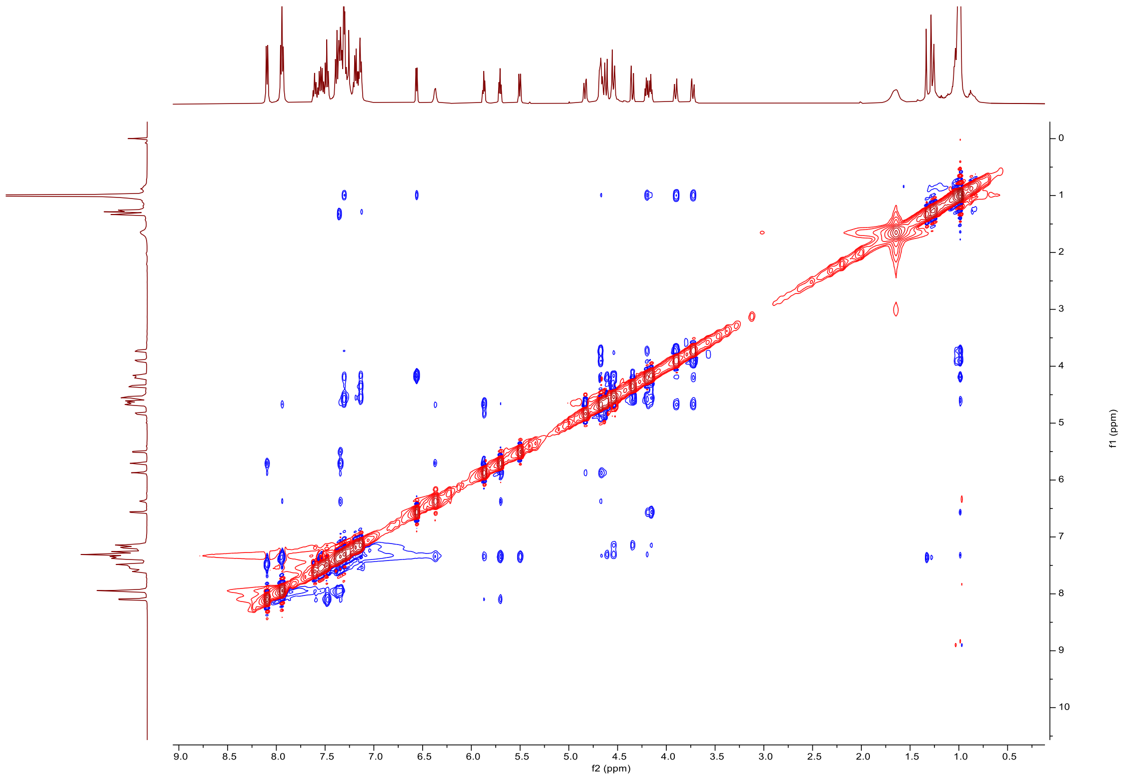

Figure S7. NOESY spectrum of compound **3α** in $CDCl_3$

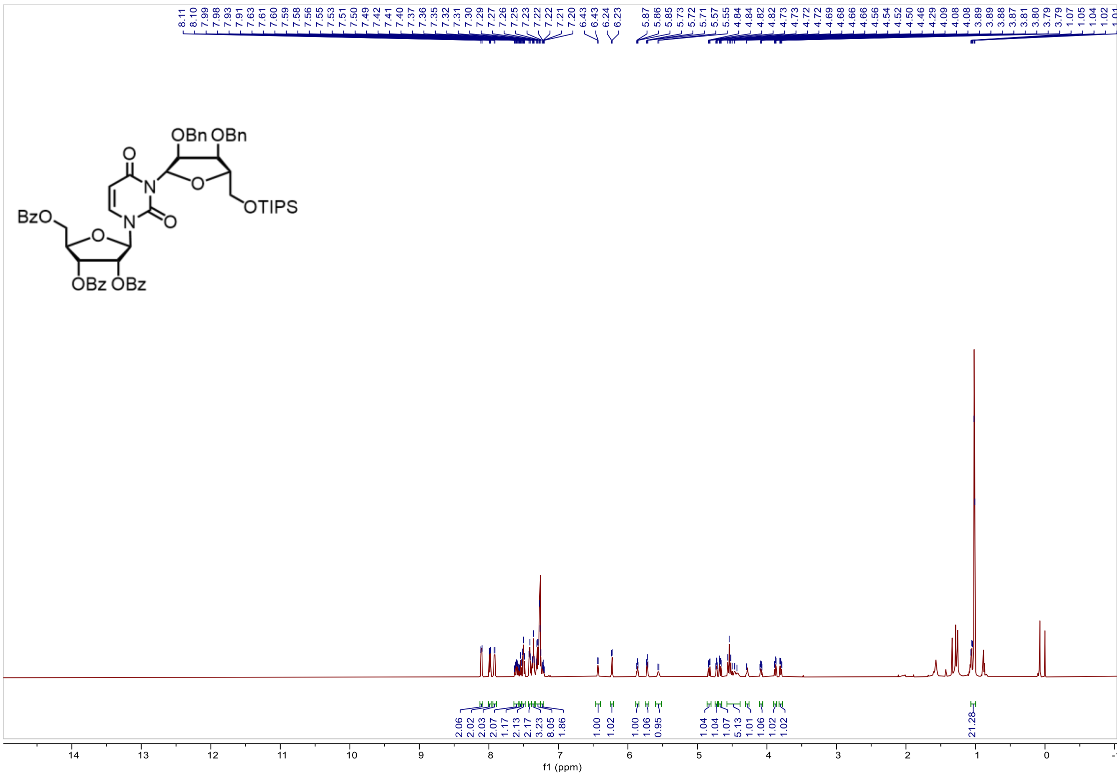

Figure S8. ^{1}H NMR spectrum of compound **3β** in $CDCl_3$

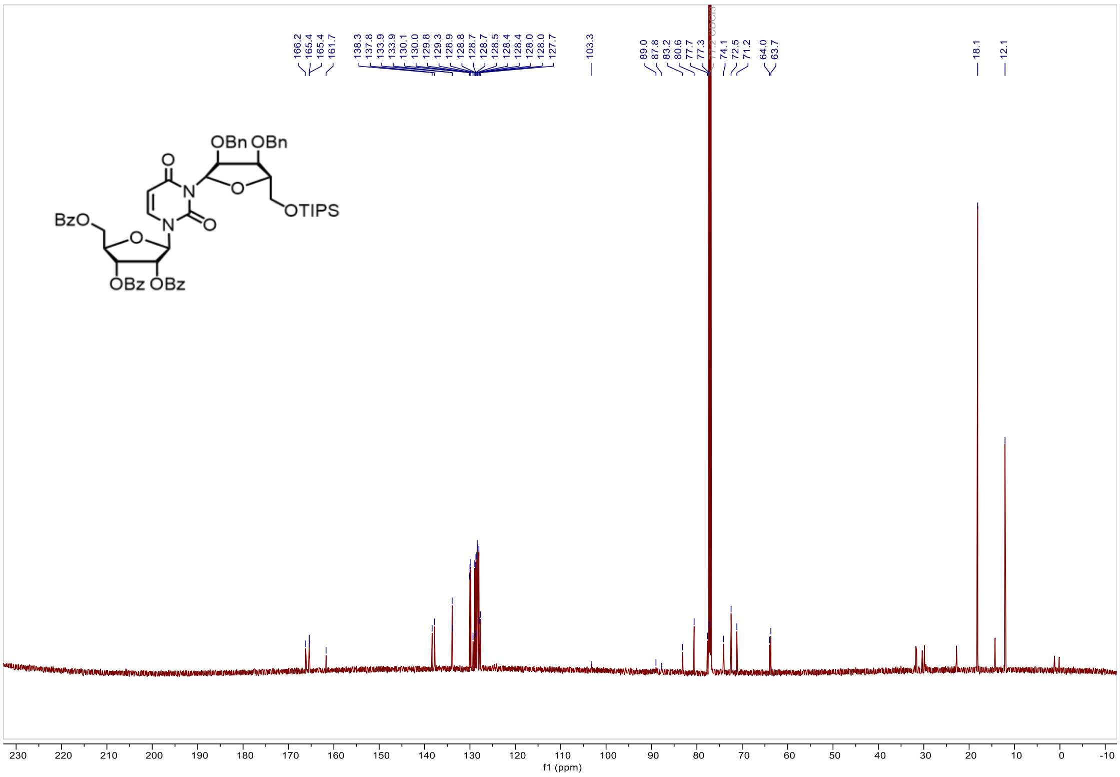

Figure S9. ^{13}C NMR spectrum of compound **3β** in $CDCl_3$

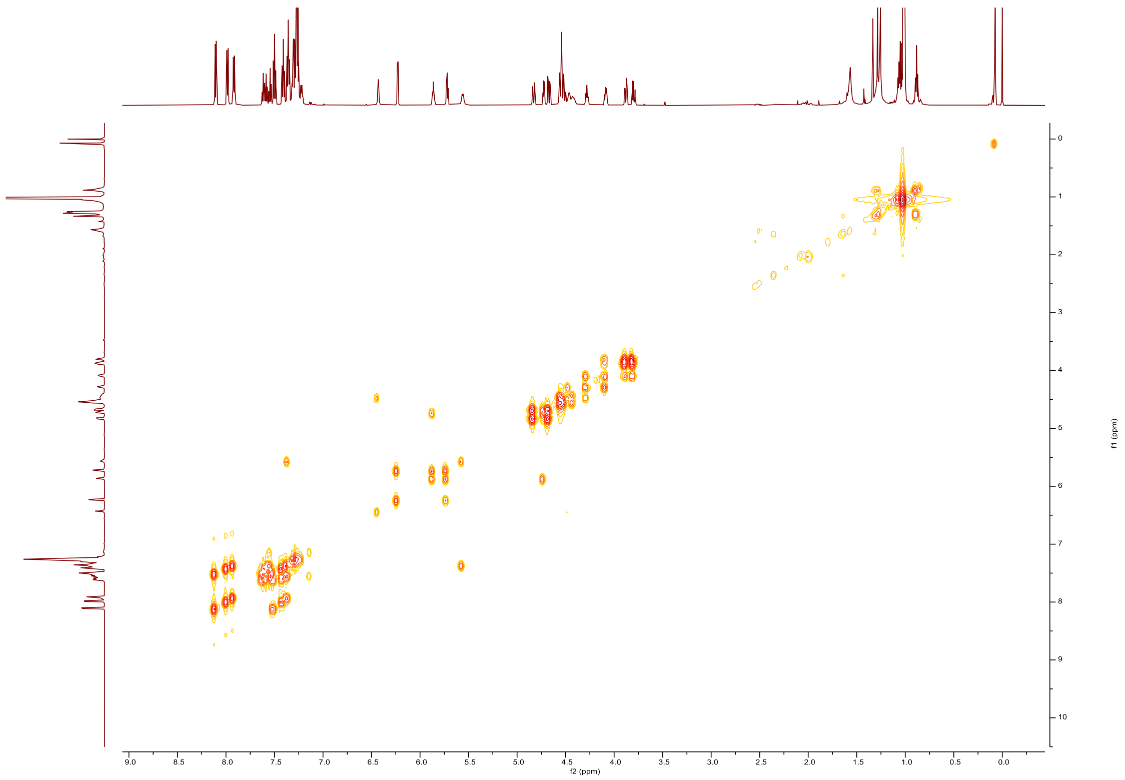

Figure S10. ^{1}H-^{1}H COSY spectrum of compound **3β** in $CDCl_3$

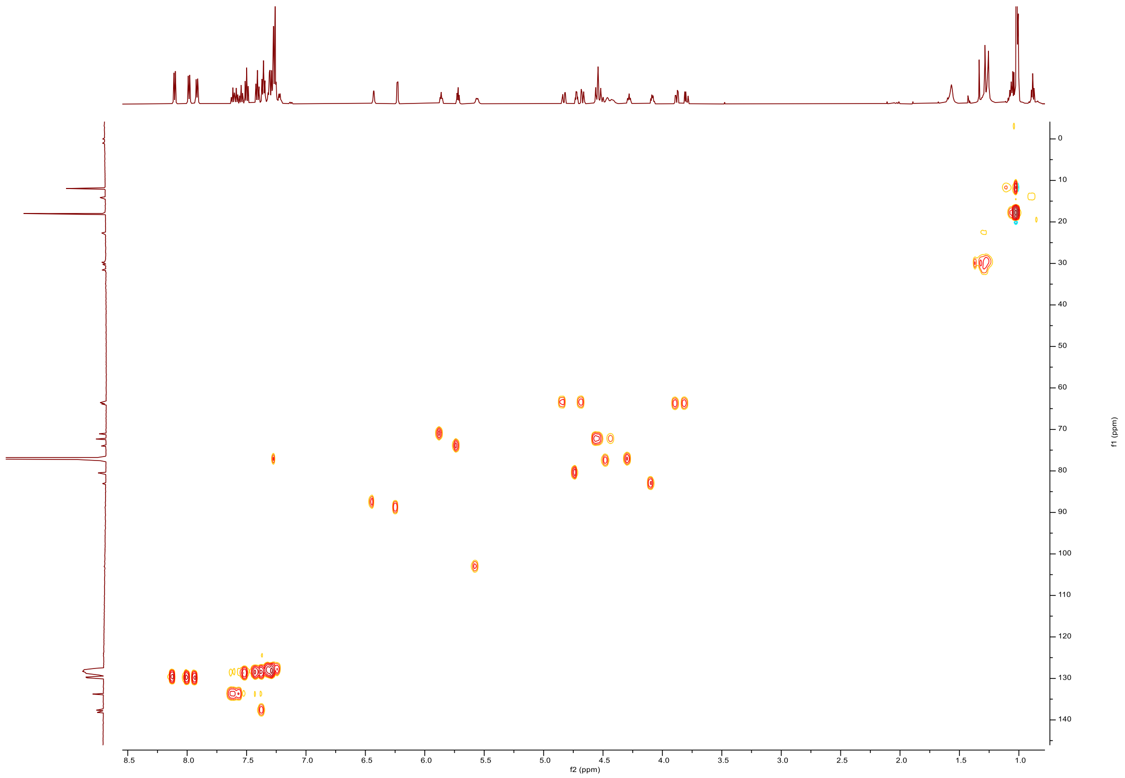

Figure S11. HSQC spectrum of compound **3β** in $CDCl_3$

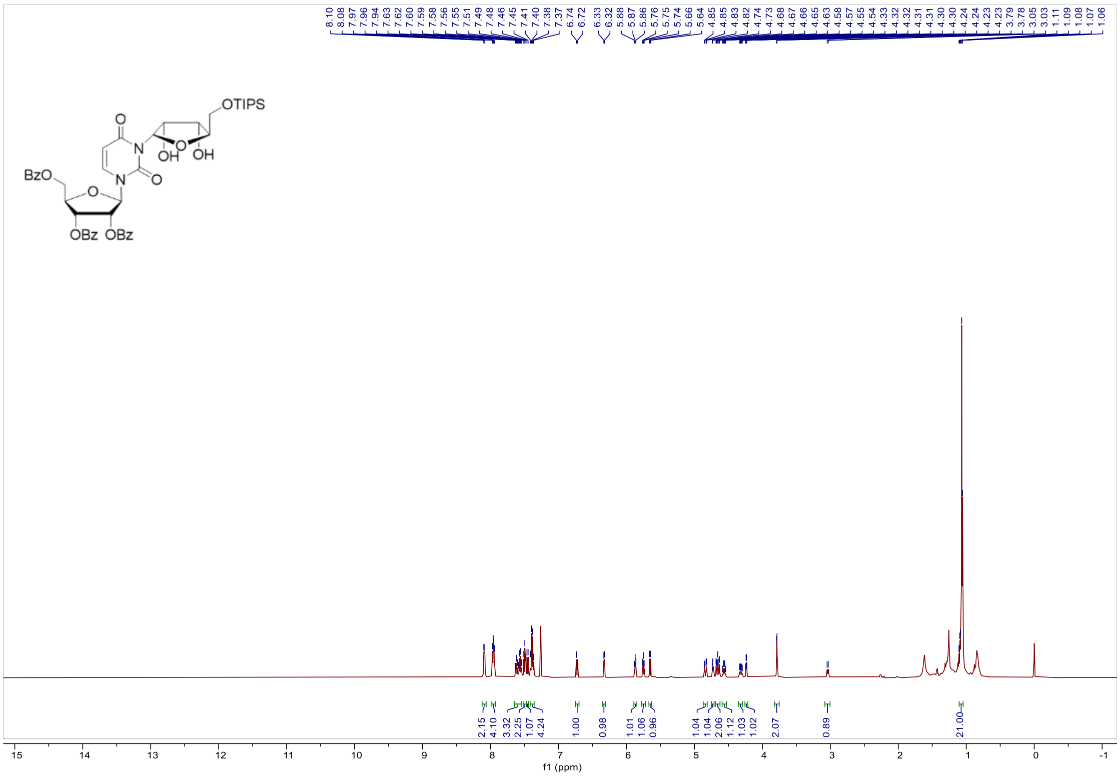

Figure S12. ^{1}H NMR spectrum of compound **10α** in $CDCl_3$

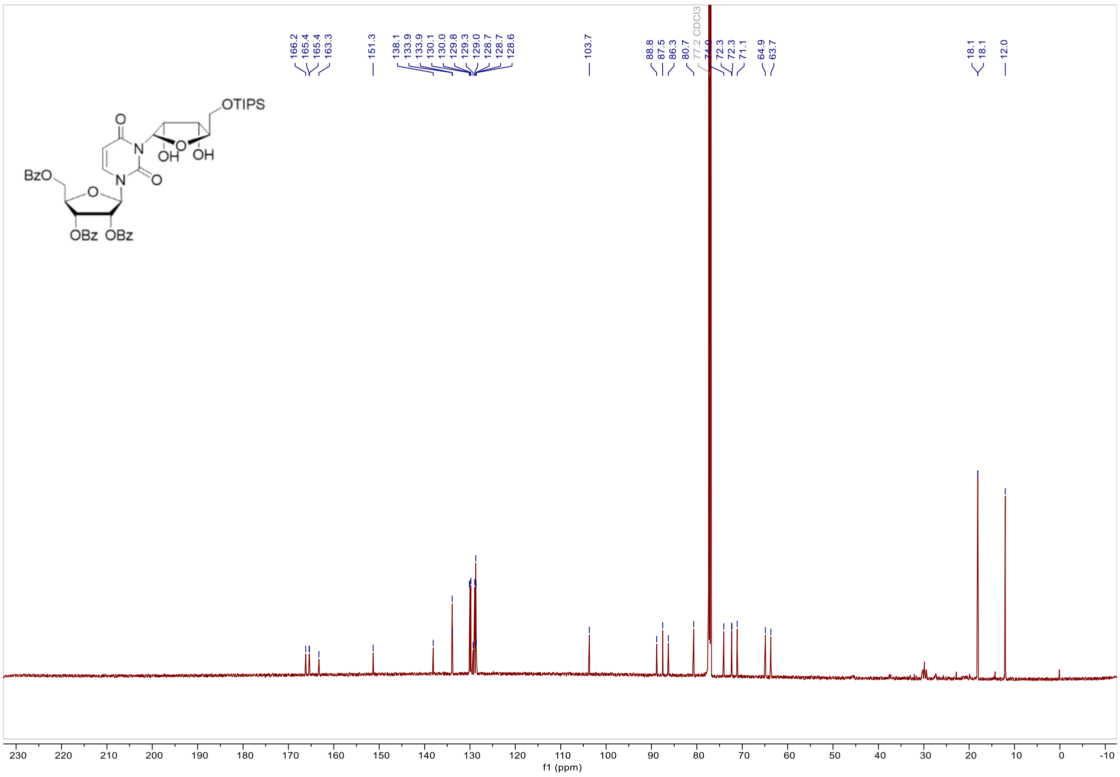

Figure S13. ^{13}C NMR spectrum of compound **10α** in $CDCl_3$

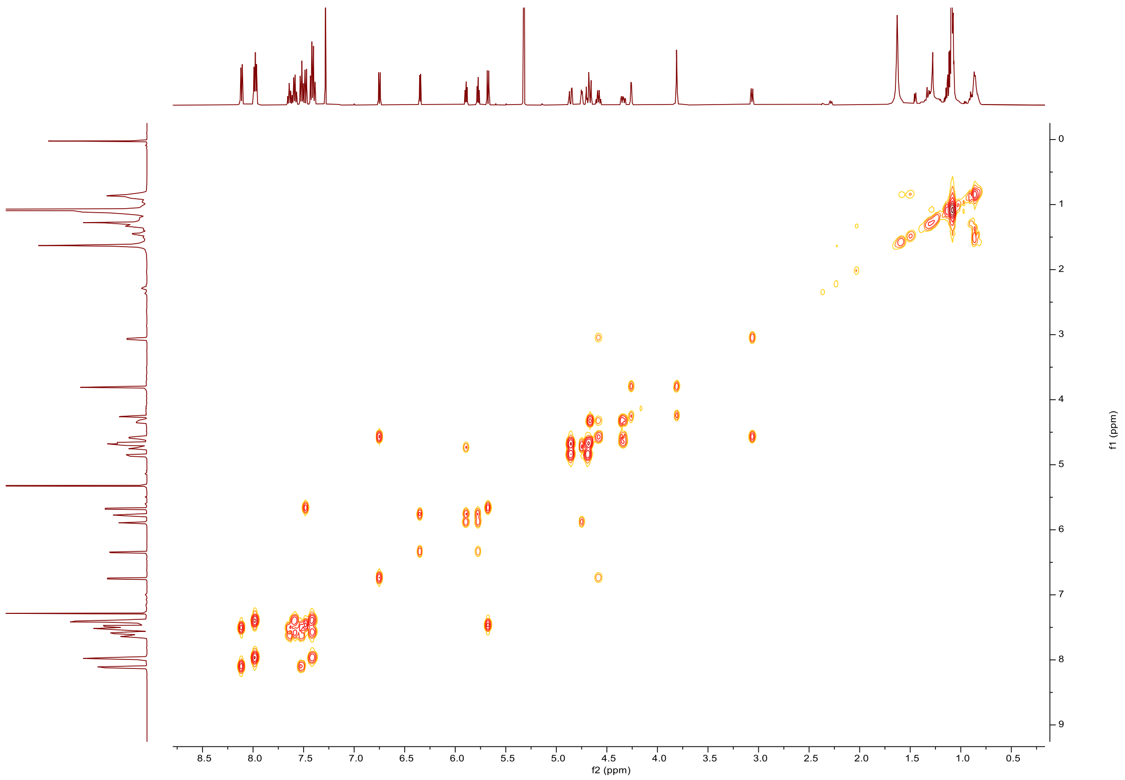

Figure S14. ^{1}H-^{1}H COSY spectrum of compound **10α** in $CDCl_3$

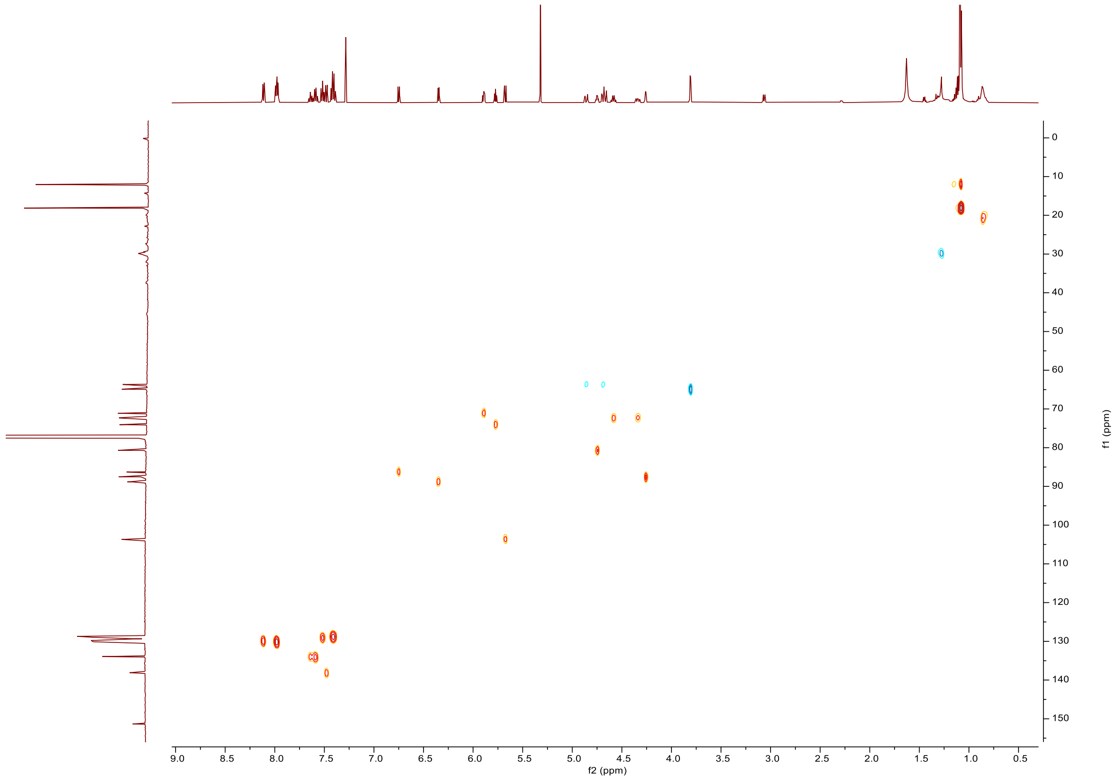

Figure S15. HSQC spectrum of compound **10α** in $CDCl_3$

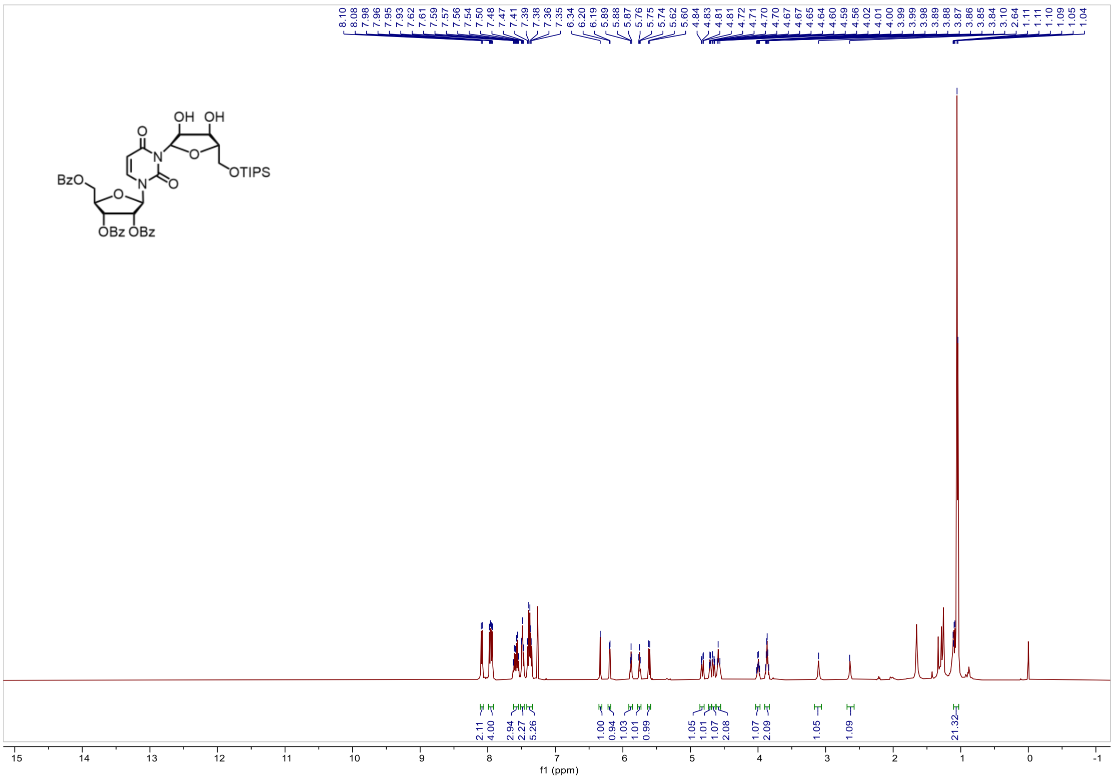

Figure S16. ^{1}H NMR spectrum of compound **10β** in $CDCl_3$

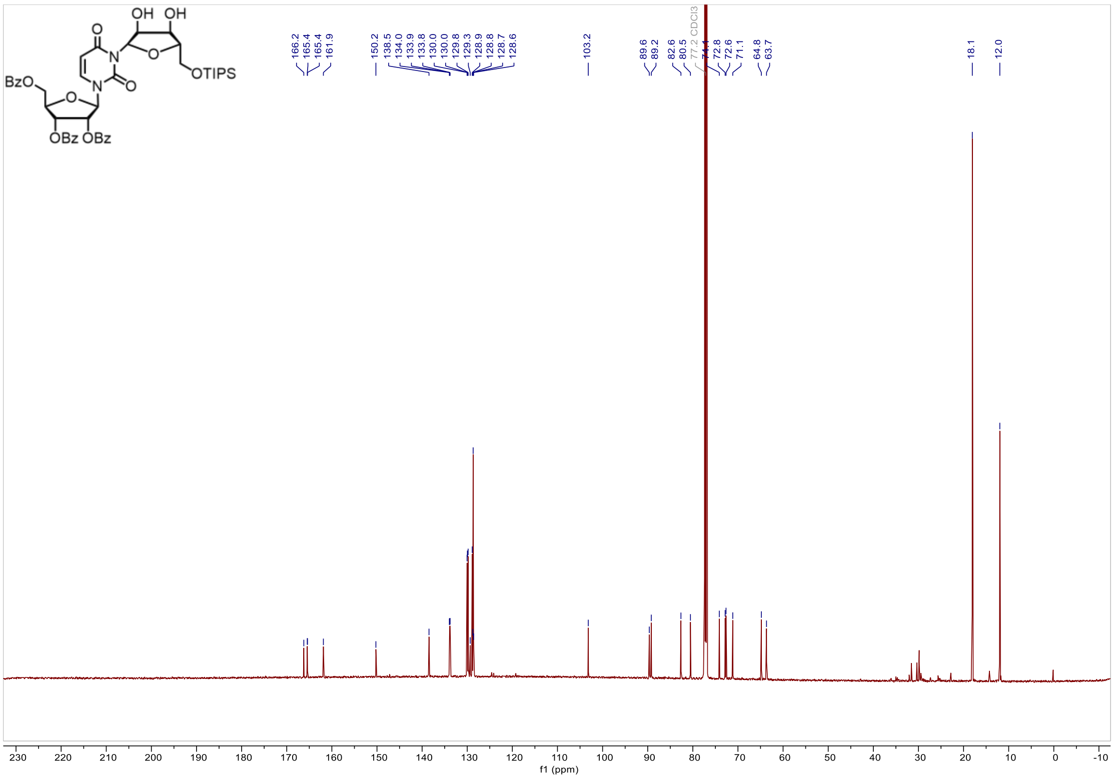

Figure S17. ^{13}C NMR spectrum of compound **10β** in $CDCl_3$

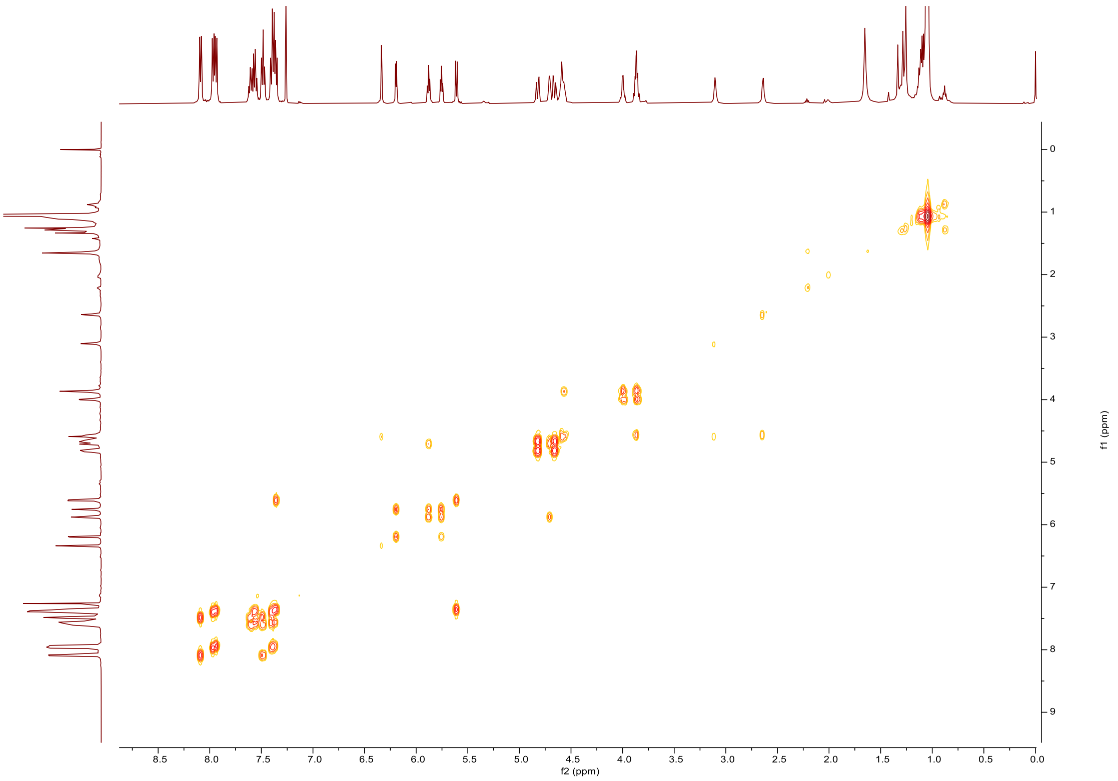

Figure S18. ^{1}H-^{1}H COSY of compound **10β** in $CDCl_3$

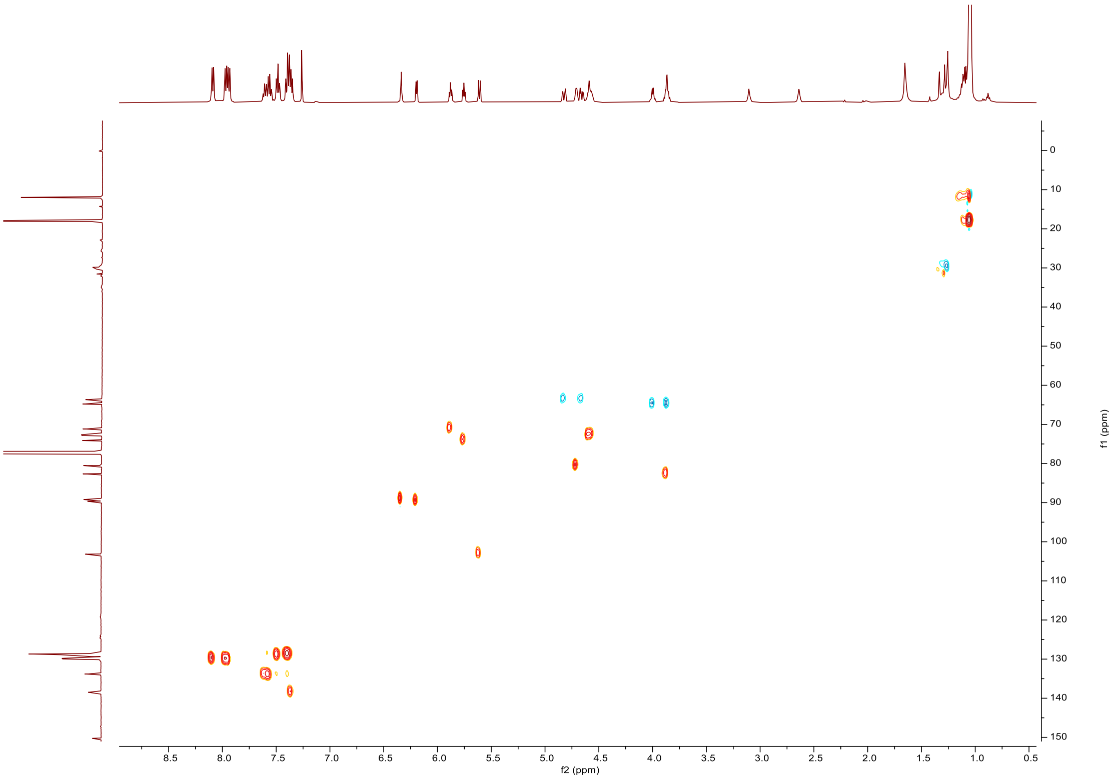

Figure S19. HSQC spectrum of compound **10β** in $CDCl_3$

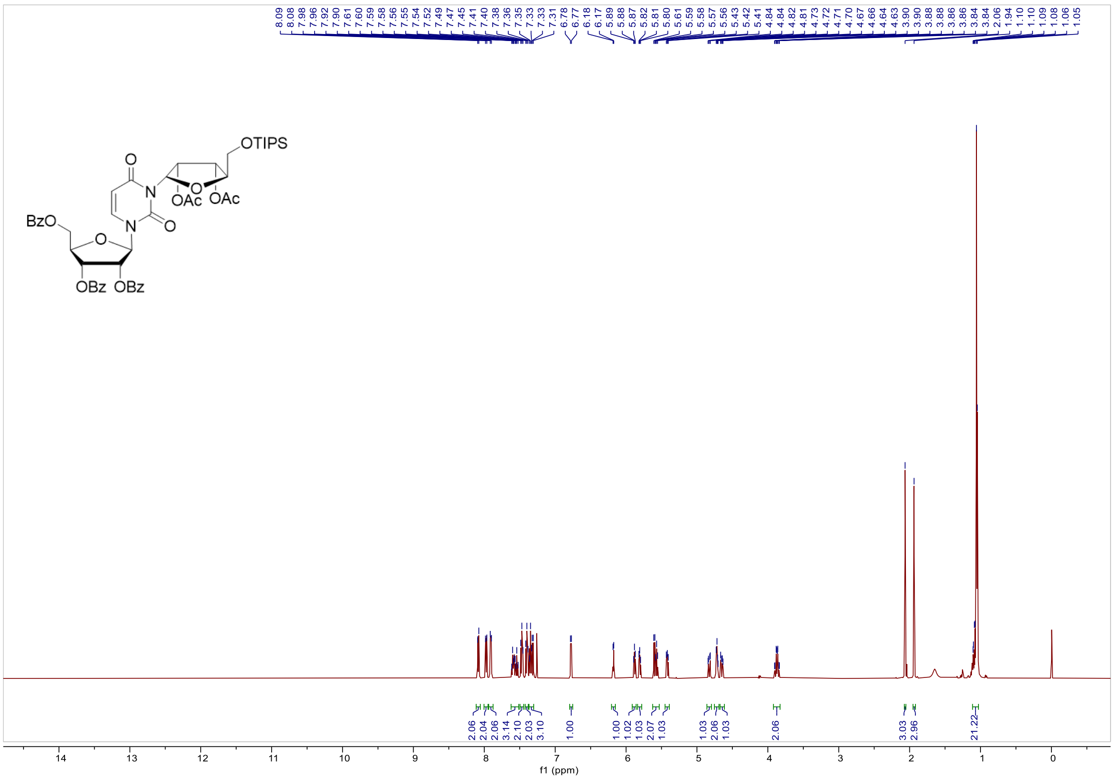

Figure S20. ^{1}H NMR spectrum of compound **11α** in $CDCl_3$

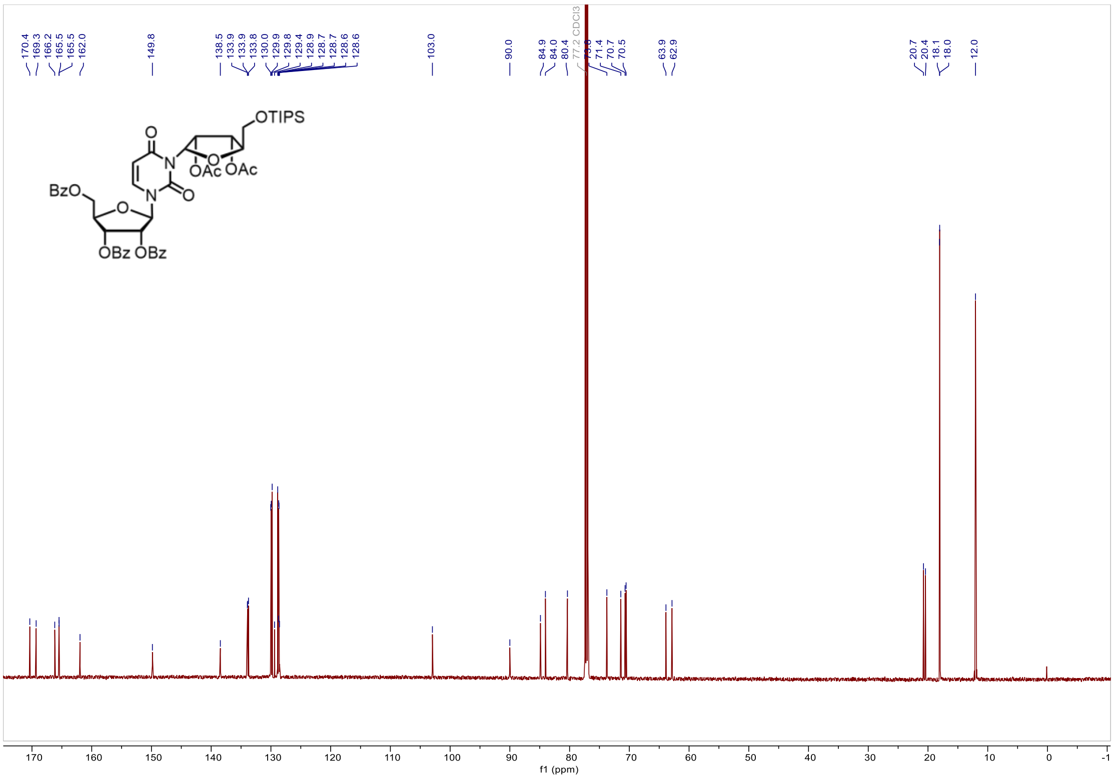

Figure S21. ^{13}C NMR spectrum of compound **11α** in $CDCl_3$

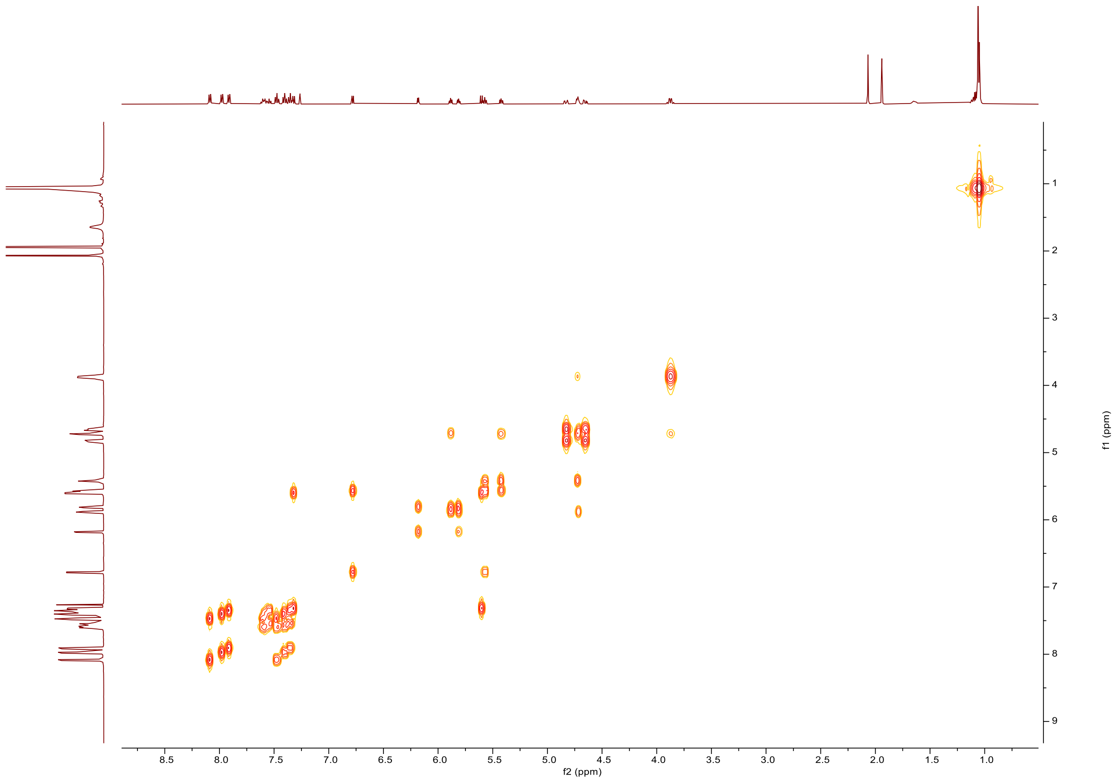

Figure S22. ^{1}H-^{1}H COSY spectrum of compound **11α** in $CDCl_3$

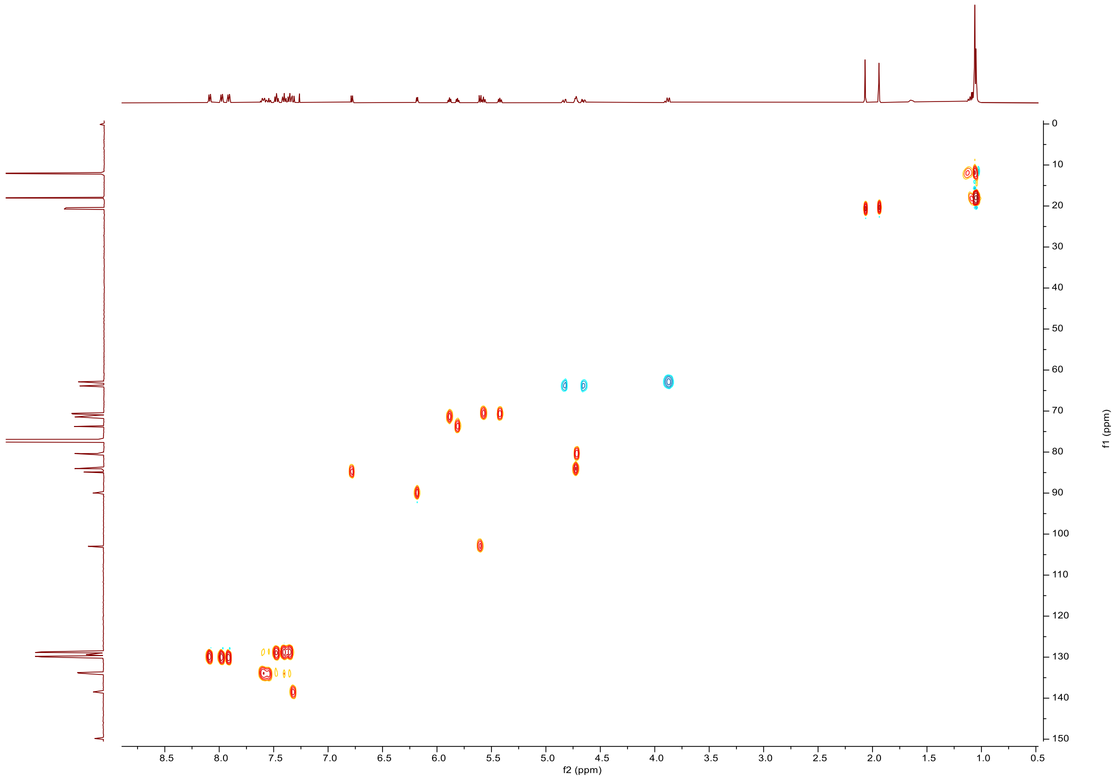

Figure S23. HSQC spectrum of compound **11α** in $CDCl_3$

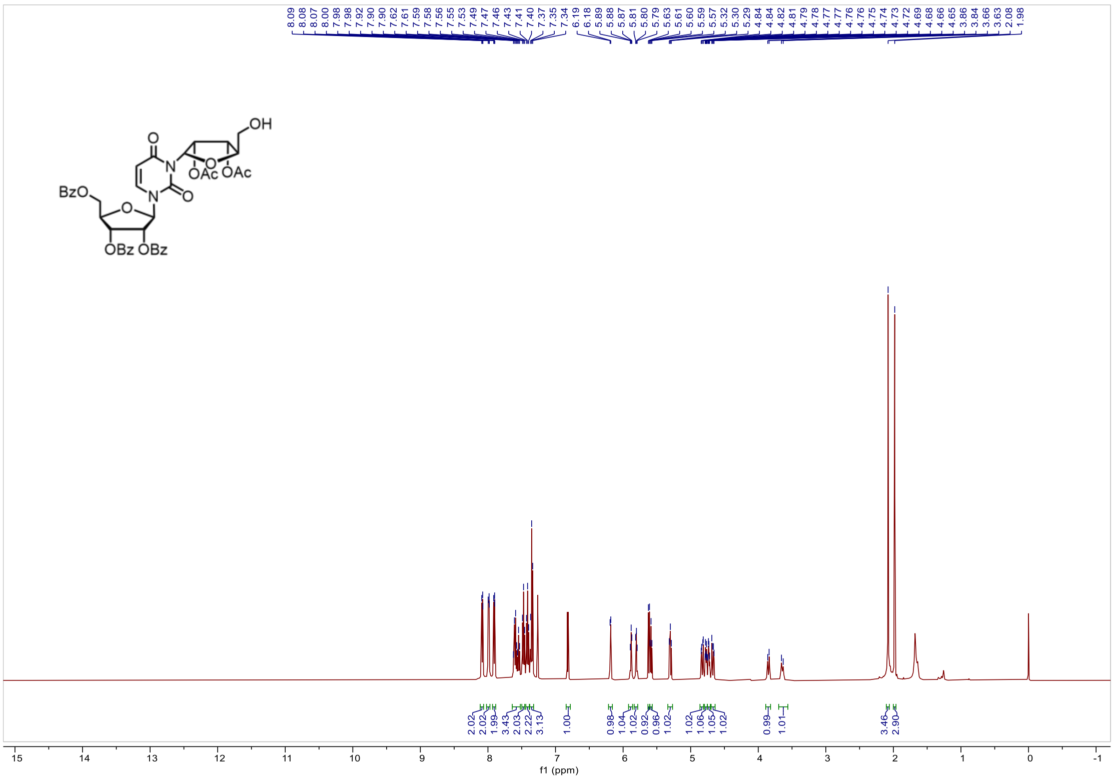

Figure S24. ^{1}H NMR spectrum of compound **12α** in $CDCl_3$

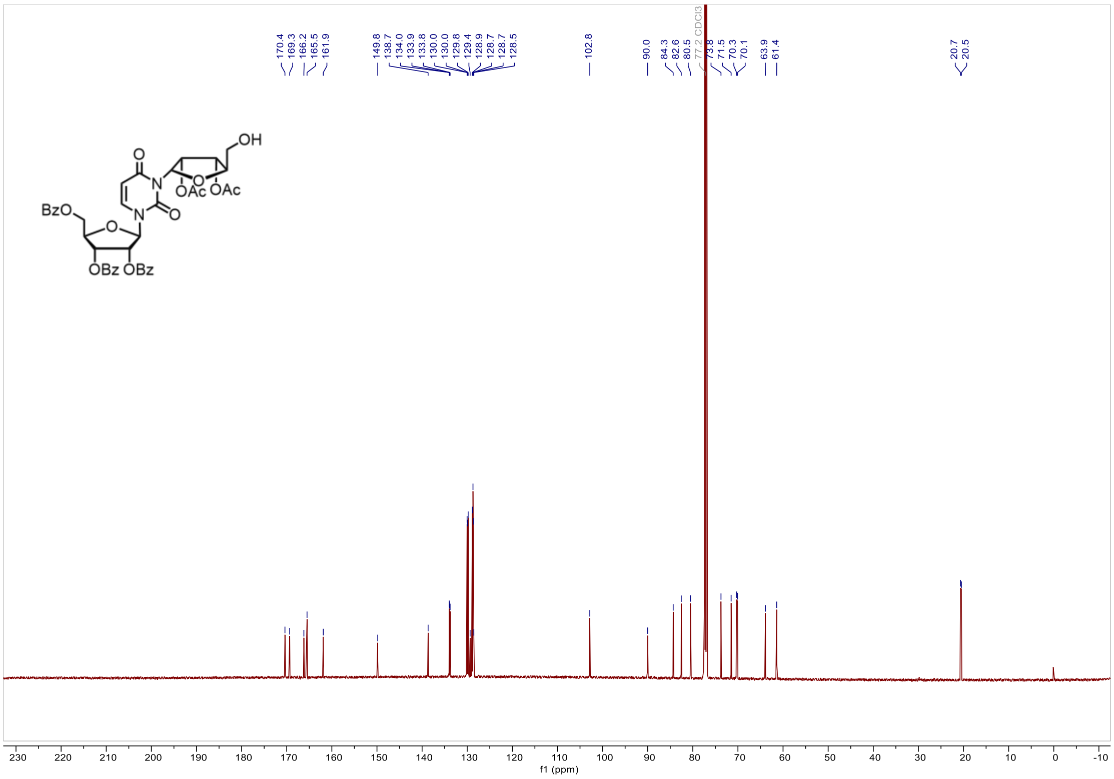

Figure S25. ^{13}C NMR spectrum of compound **12α** in $CDCl_3$

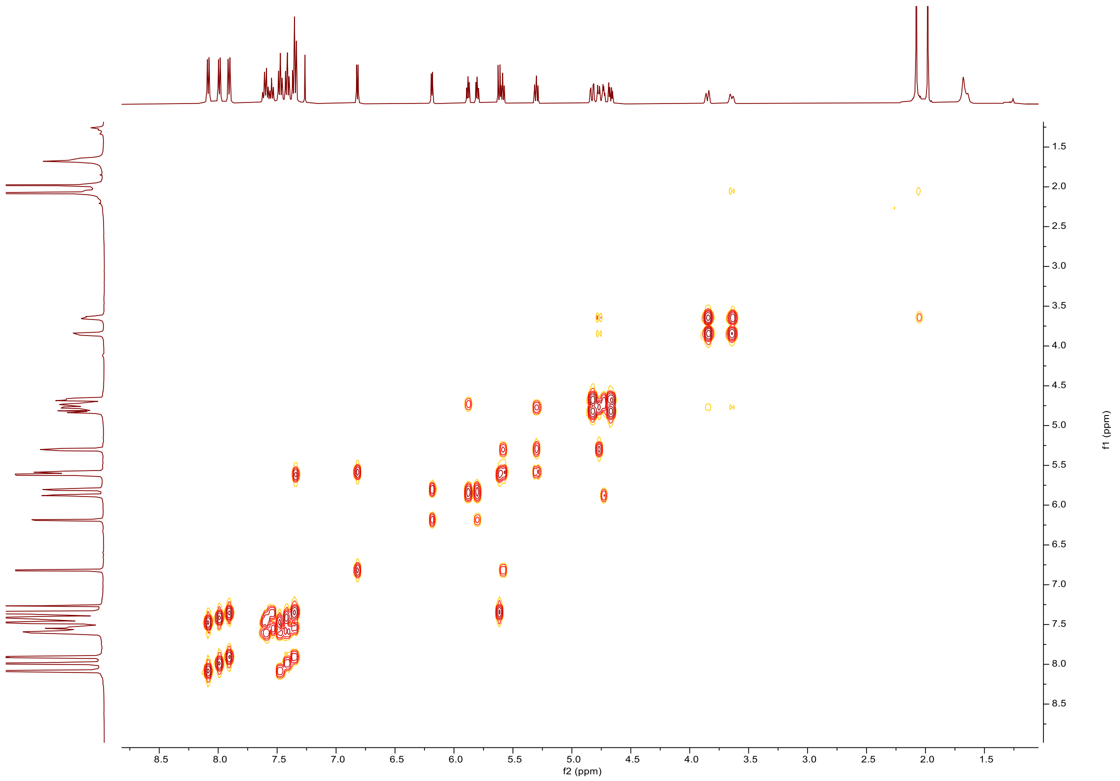

Figure S26. ^{1}H-^{1}H COSY spectrum of compound **12α** in $CDCl_3$

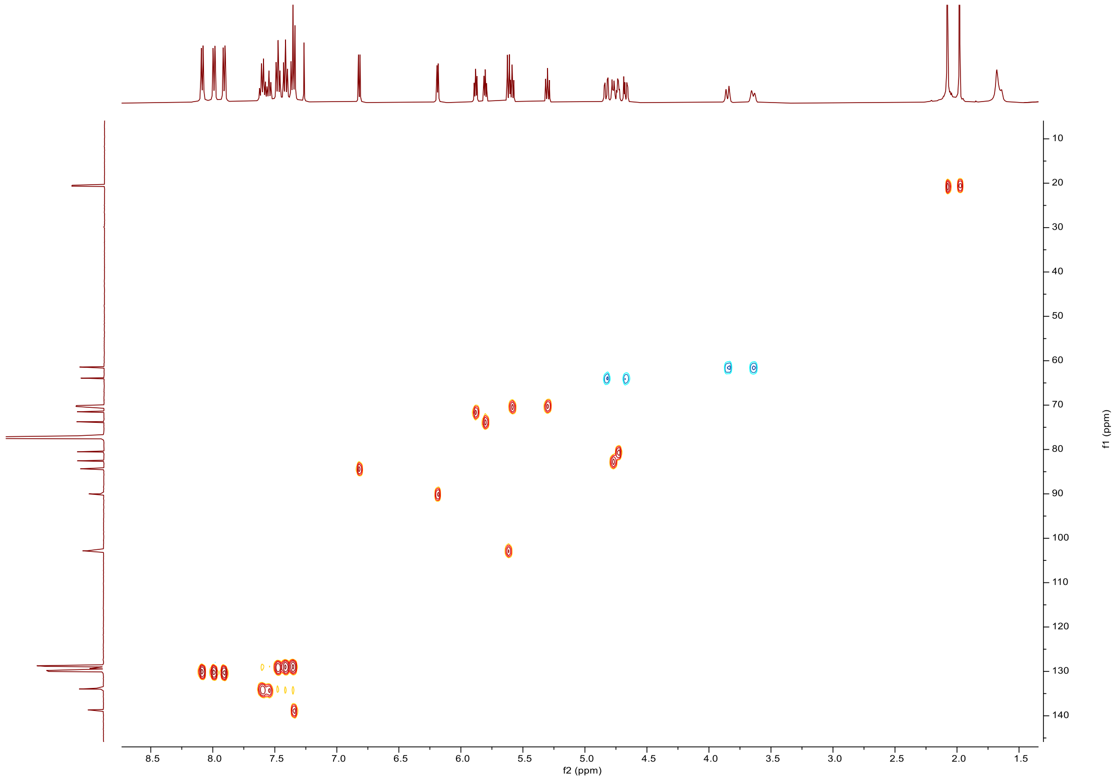

Figure S27. HSQC spectrum of compound **12α** in $CDCl_3$

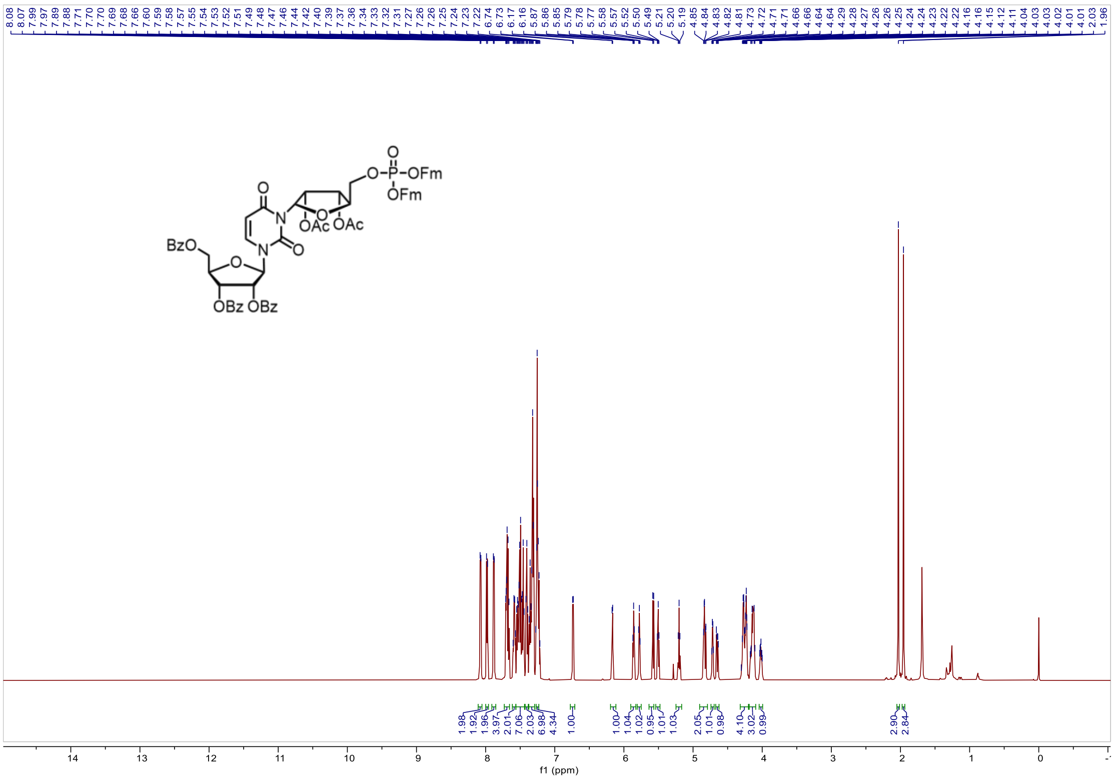

Figure S28. ^{1}H NMR spectrum of compound **13α** in $CDCl_3$

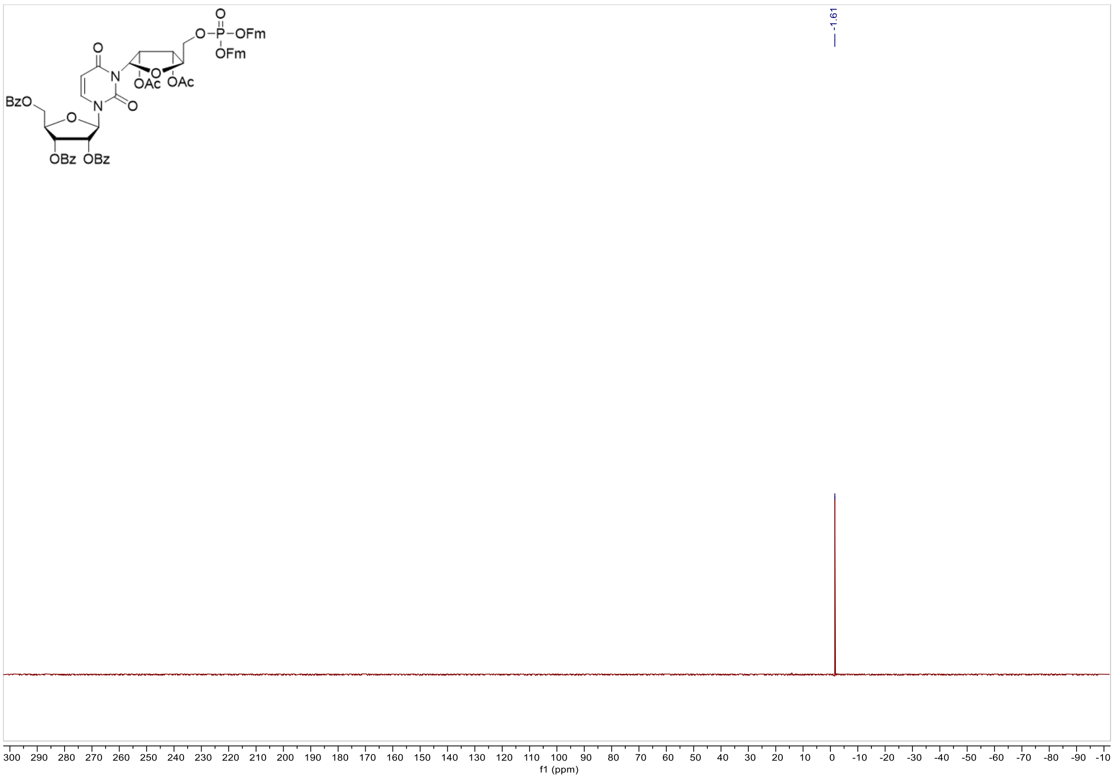

Figure S29. ^{31}P NMR spectrum of compound **13α** in $CDCl_3$

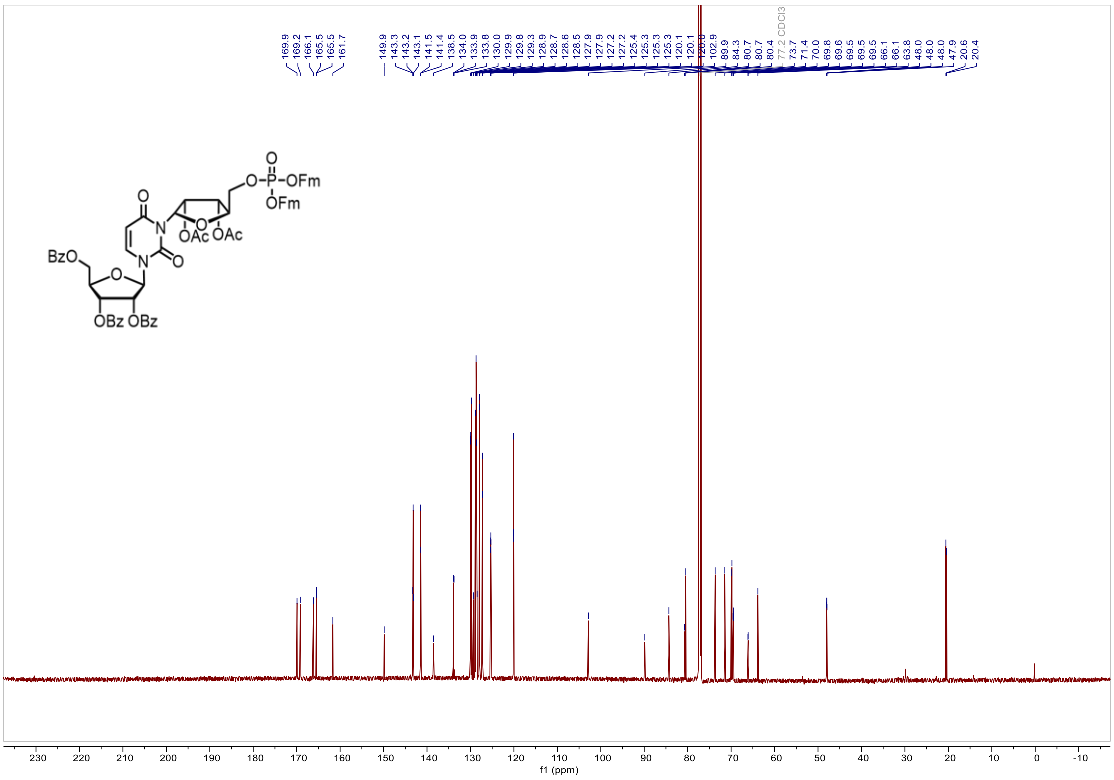

Figure 30. ^{13}C NMR spectrum of compound **13α** in $CDCl_3$

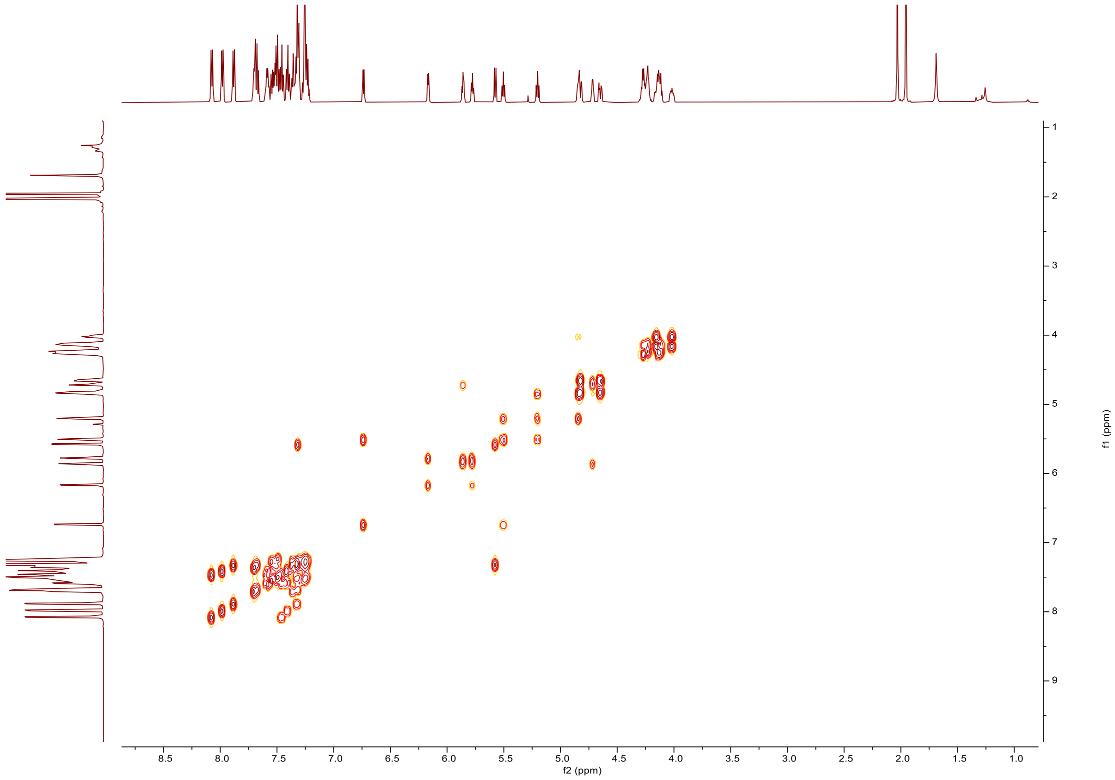

Figure S31. ^{1}H-^{1}H COSY spectrum of compound **13α** in $CDCl_3$

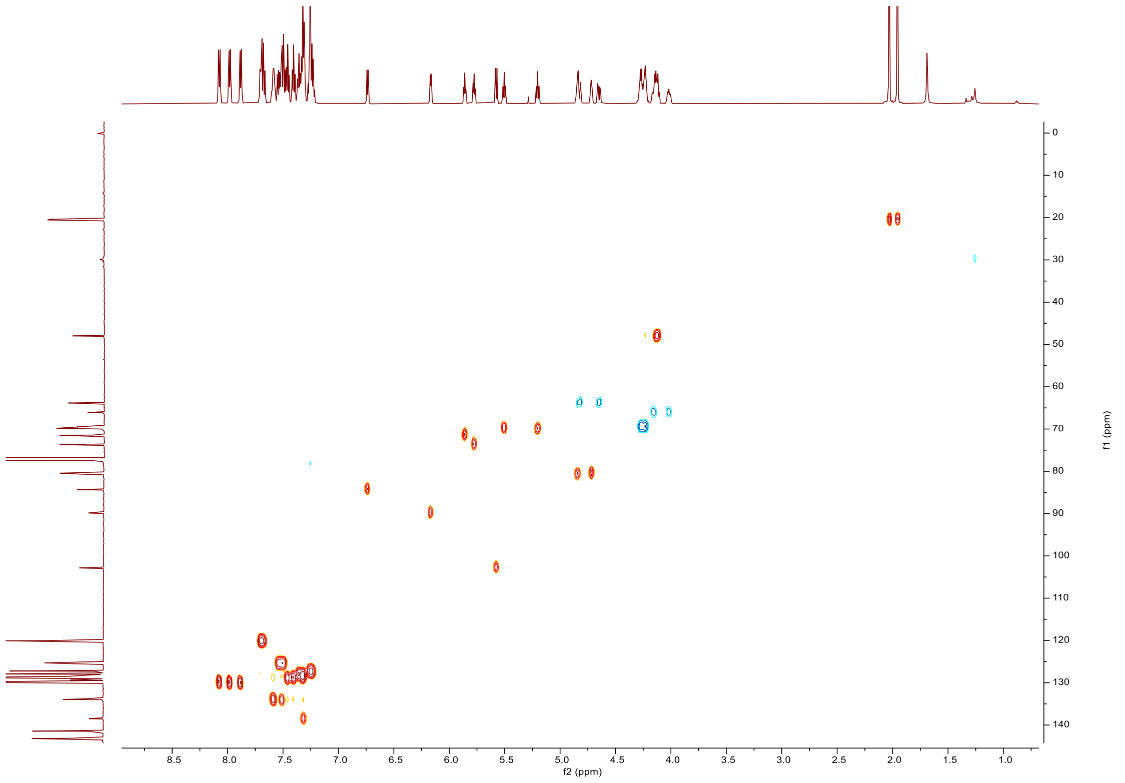

Figure S32. HSQC spectrum of compound **13α** in $CDCl_3$

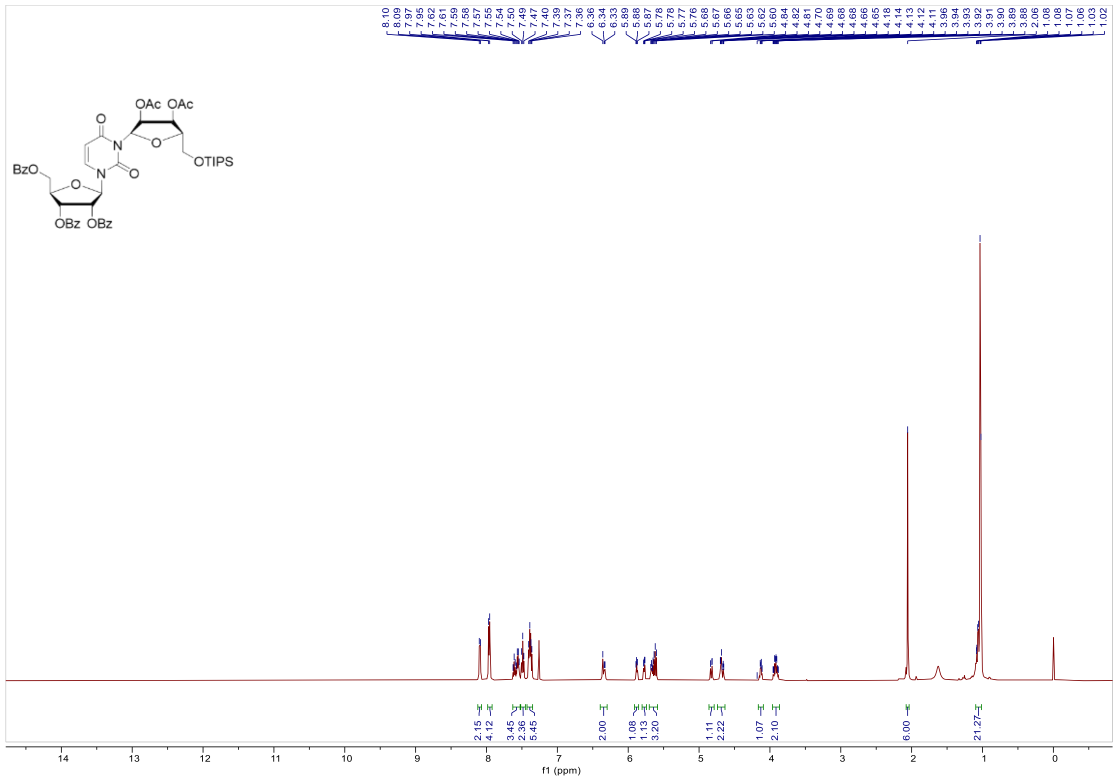

Figure S33. ^{1}H NMR spectrum of compound **11β** in $CDCl_3$

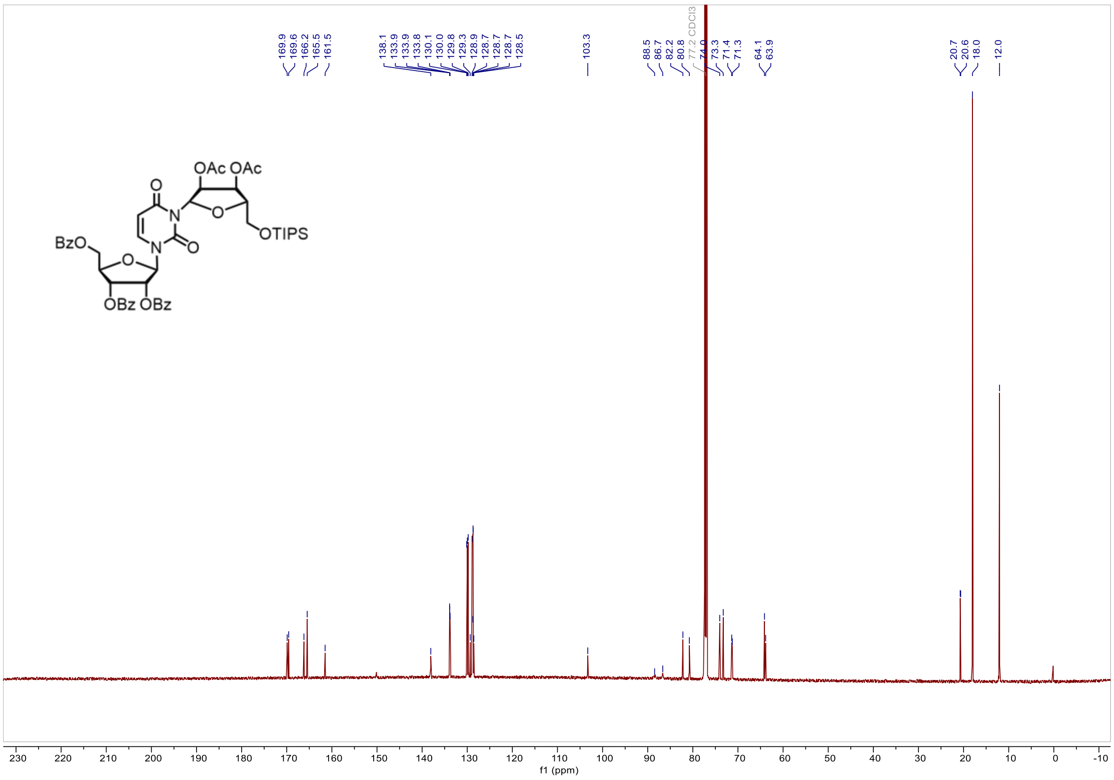

Figure S34. ^{13}C NMR spectrum of compound **11β** in $CDCl_3$

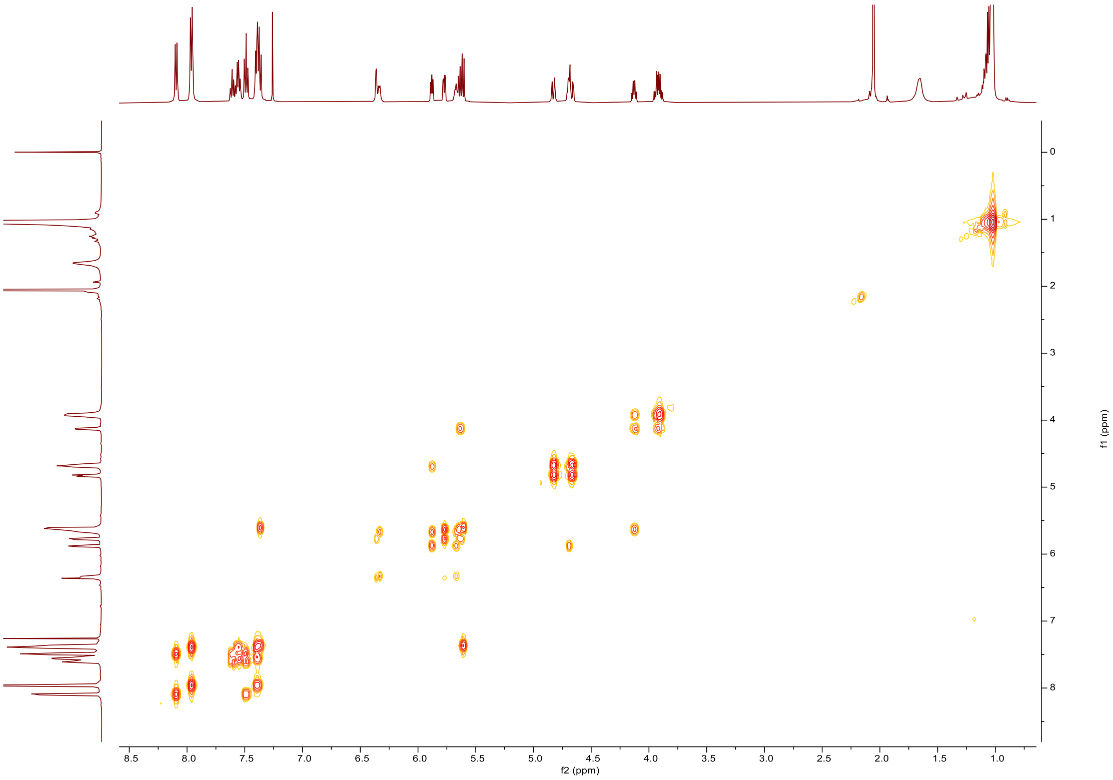

Figure S35. ^{1}H-^{1}H COSY spectrum of compound **11β** in $CDCl_3$

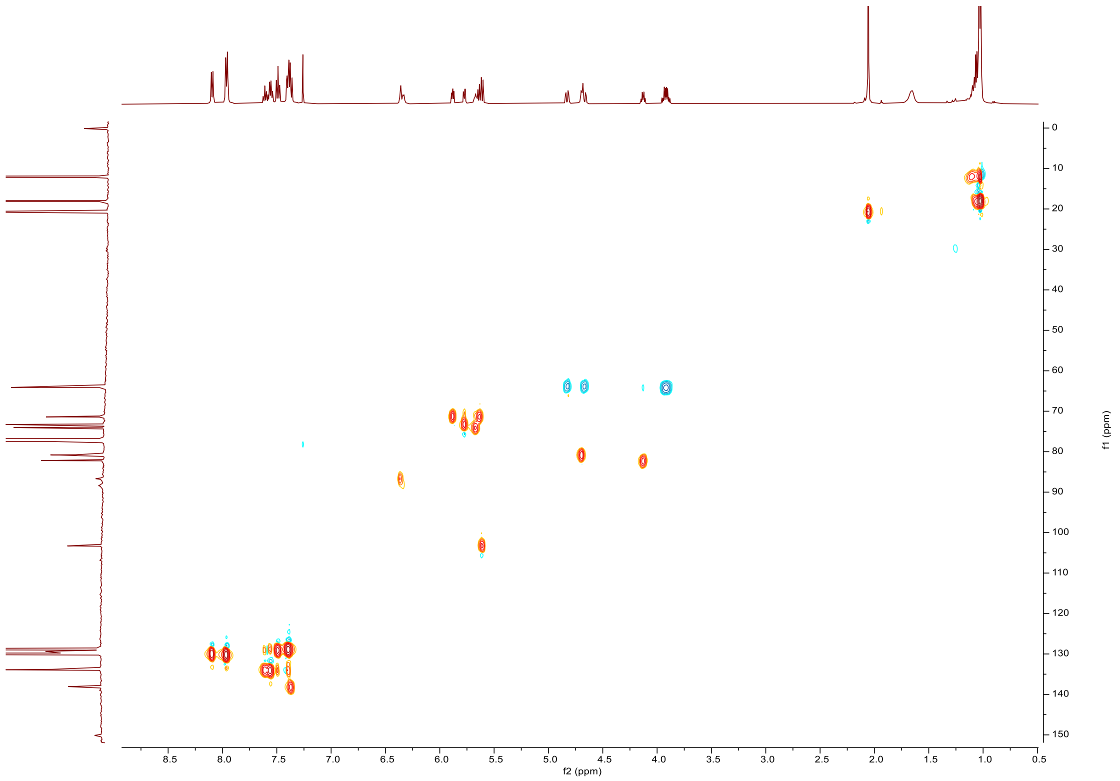

Figure S36. HSQC spectrum of compound **11β** in $CDCl_3$

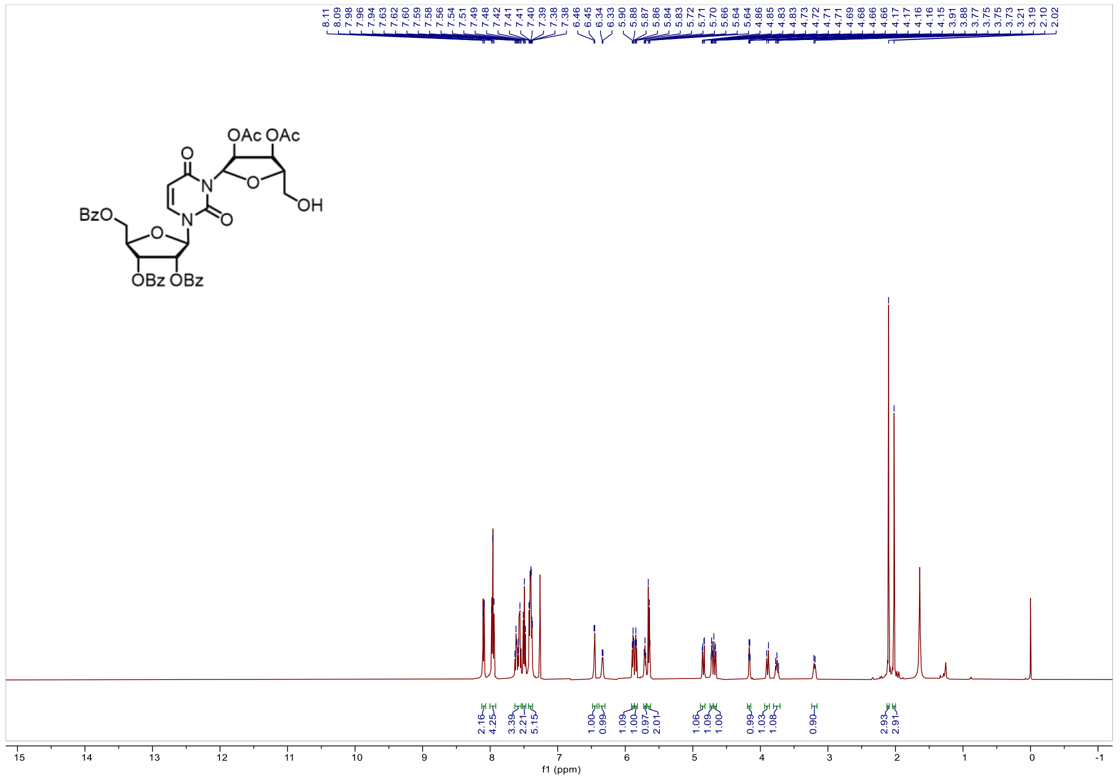

Figure S37. ^1H NMR spectrum of compound **12β** in $CDCl_3$

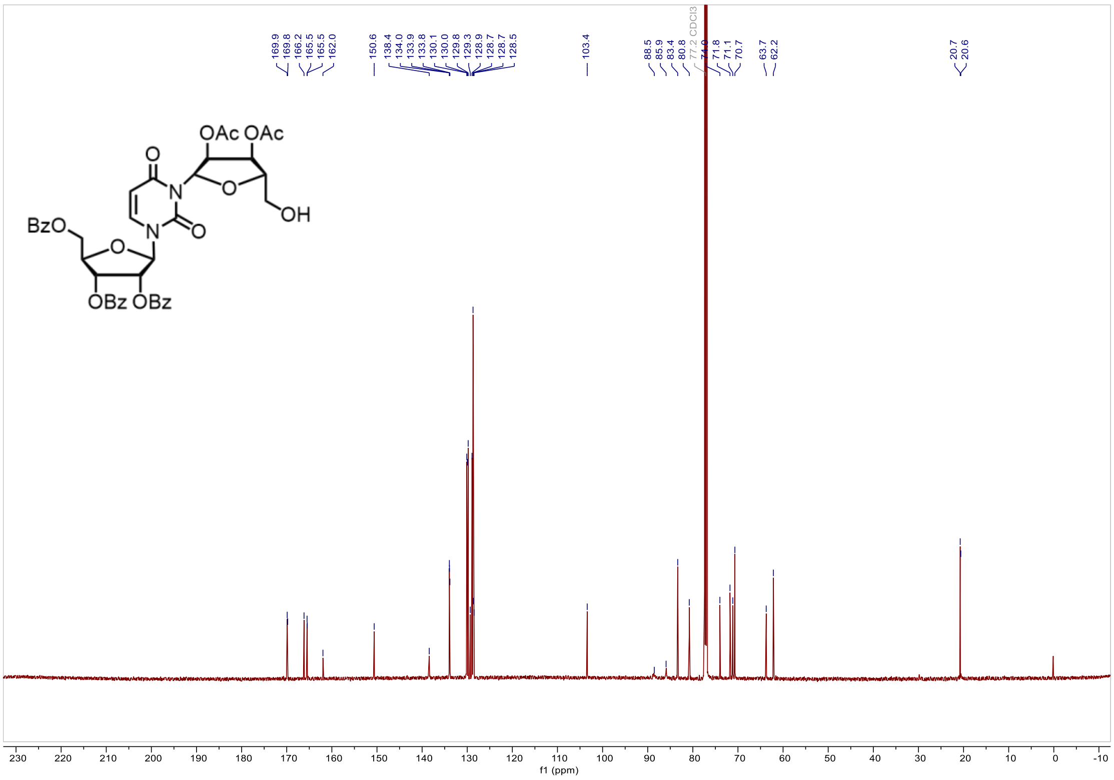

Figure S38. ^{13}C NMR spectrum of compound **12β** in $CDCl_3$

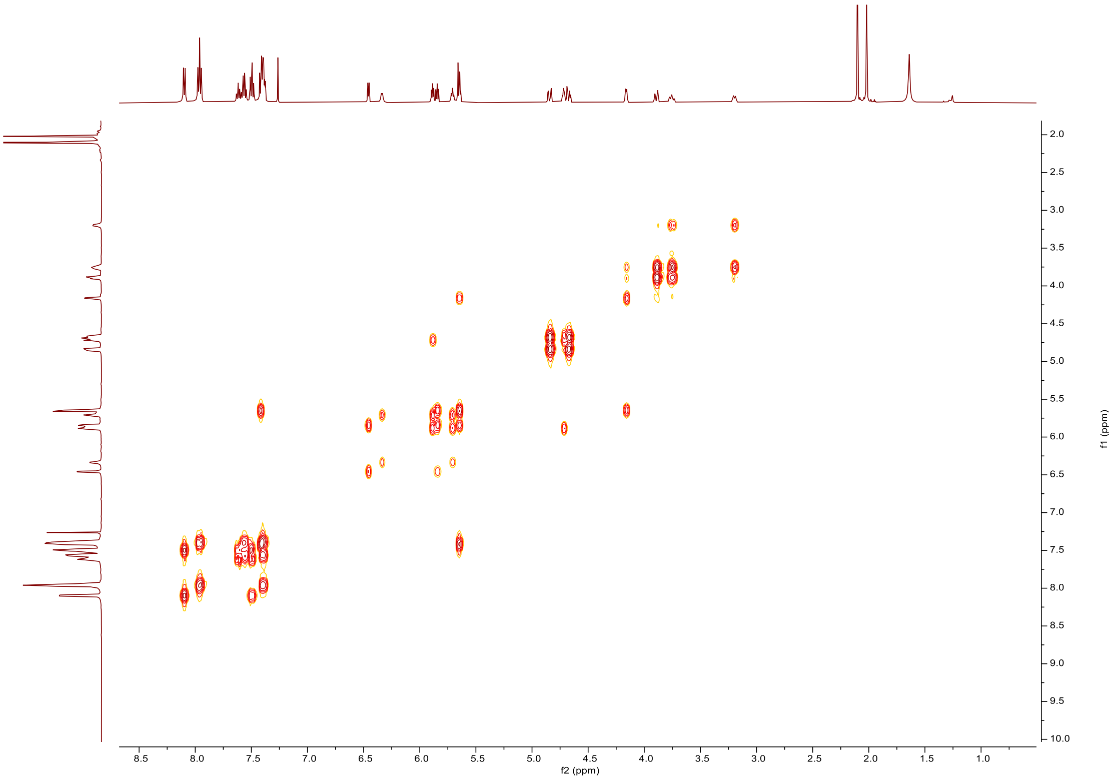

Figure S39. ^{1}H-^{1}H COSY spectrum of compound **12β** in $CDCl_3$

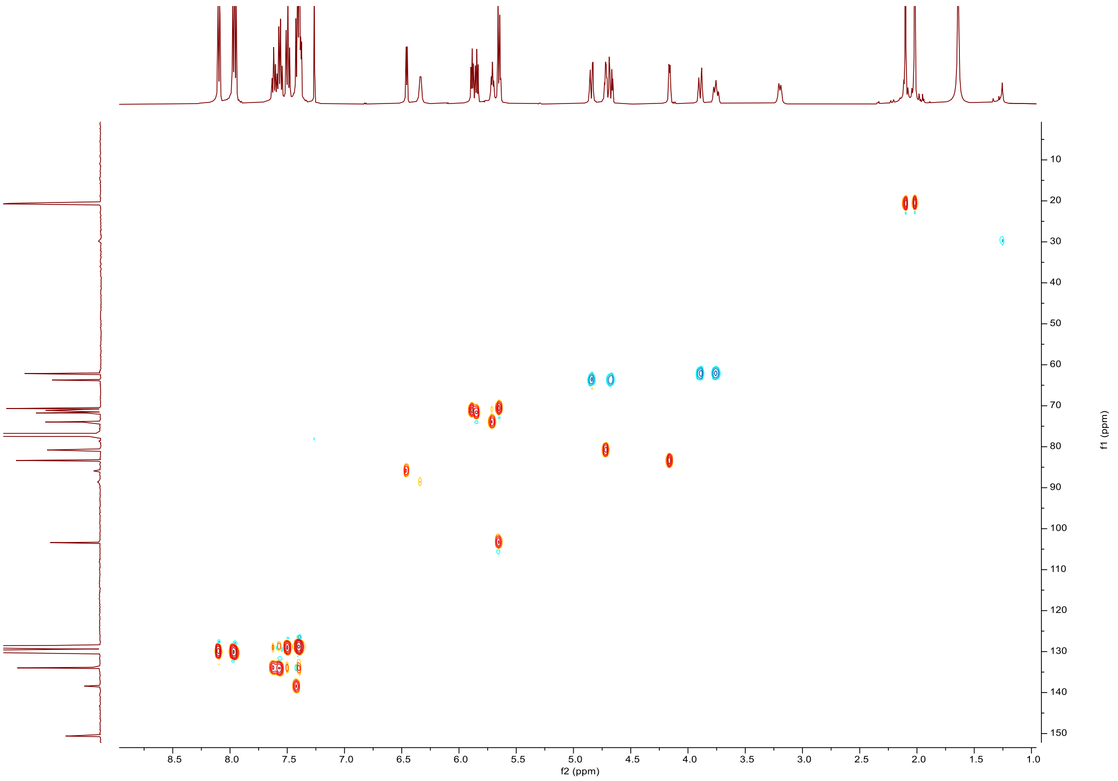

Figure S40. HSQC spectrum of compound **12β** in $CDCl_3$

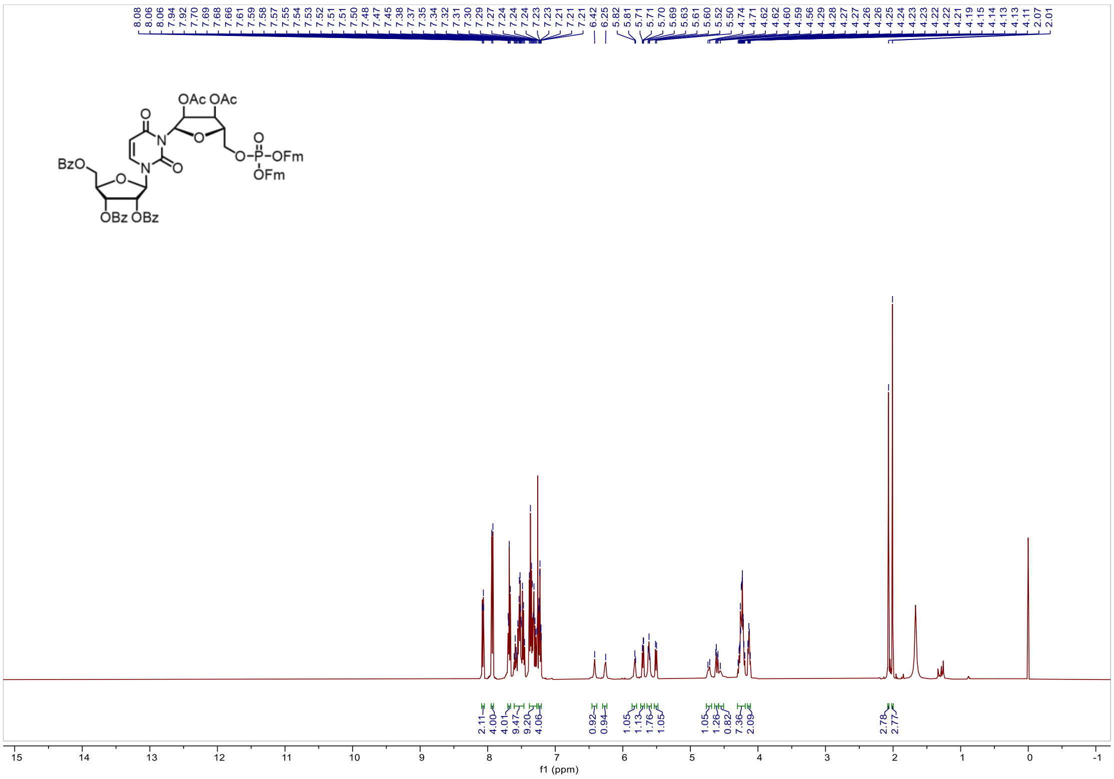

Figure S41. ^{1}H NMR spectrum of compound **13β** in $CDCl_3$

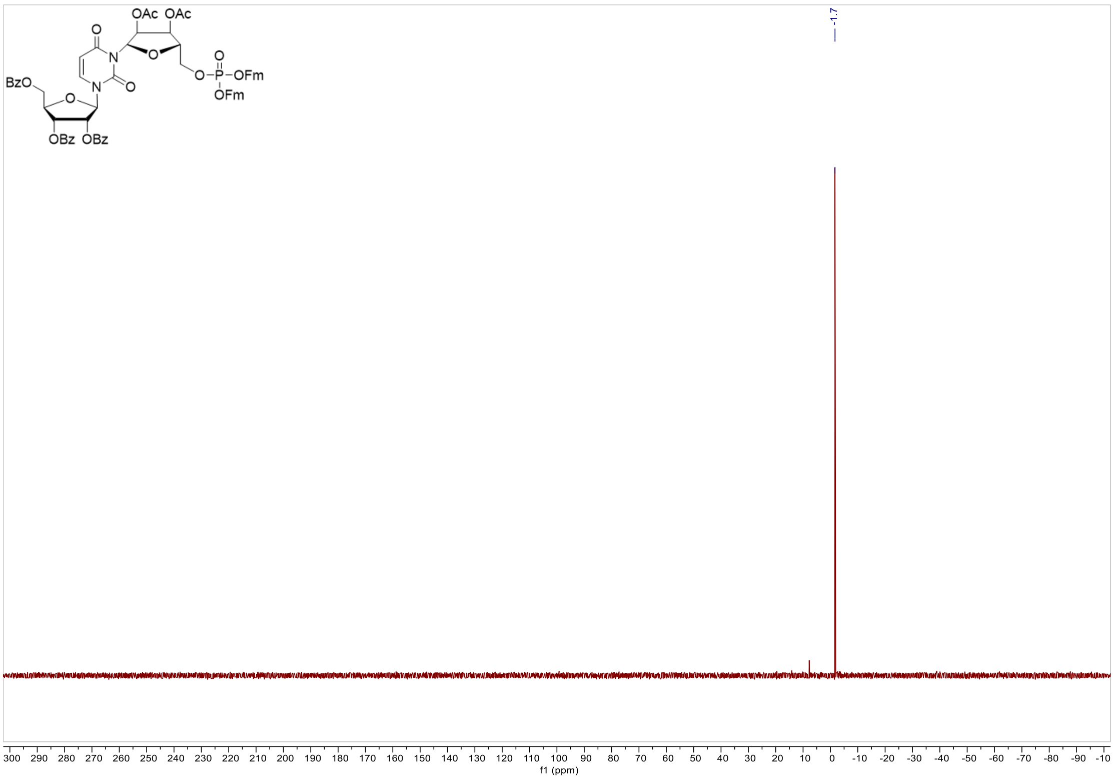

Figure S42. ^{31}P NMR spectrum of compound **13β** in $CDCl_3$

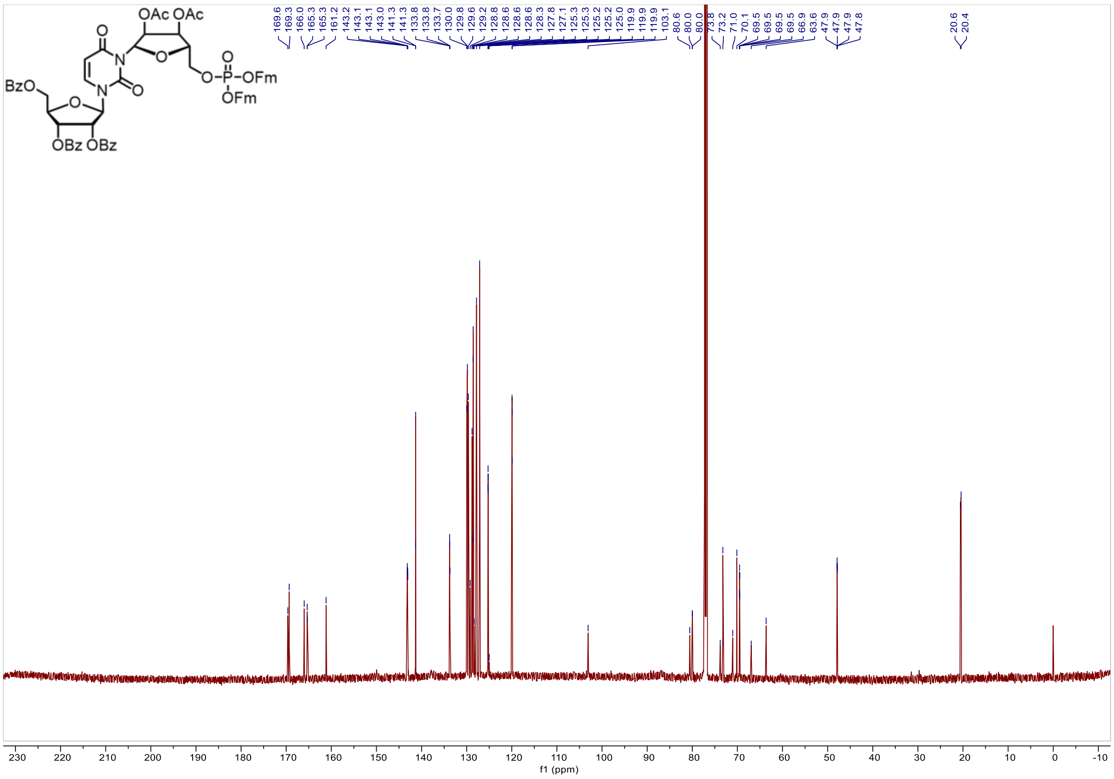

Figure S43. ^{13}C NMR spectrum of compound **13β** in $CDCl_3$

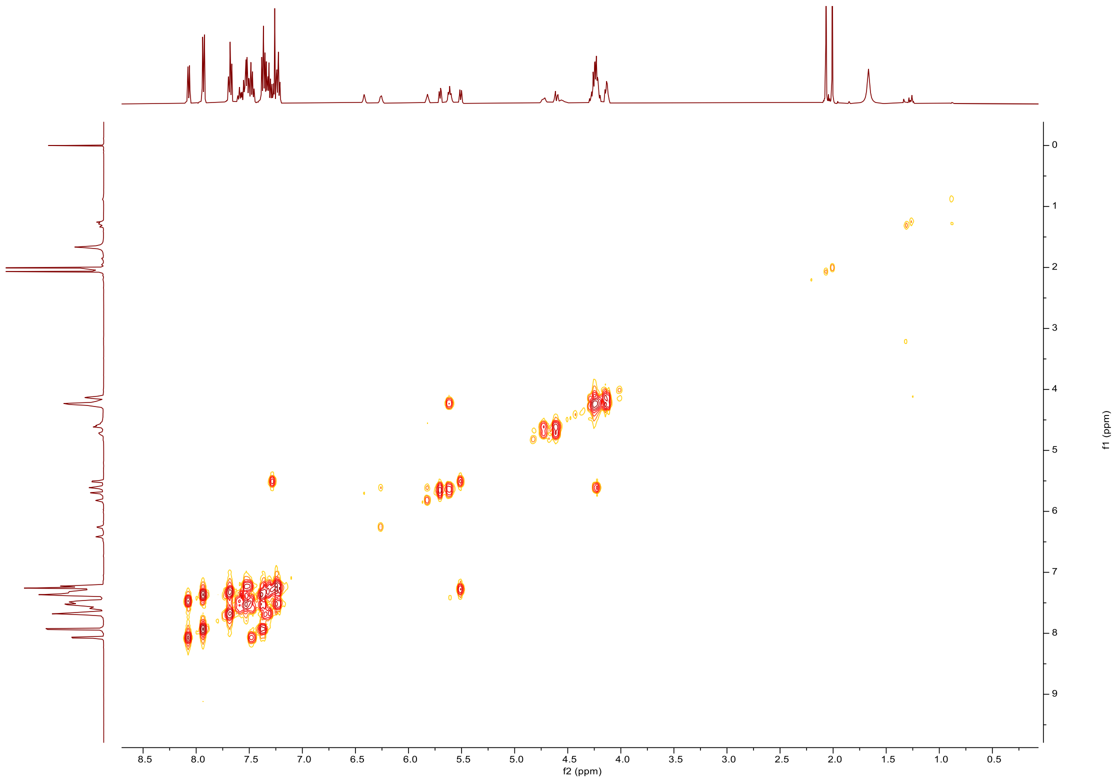

Figure S44. ^{1}H-^{1}H COSY spectrum of compound **13β** in $CDCl_3$

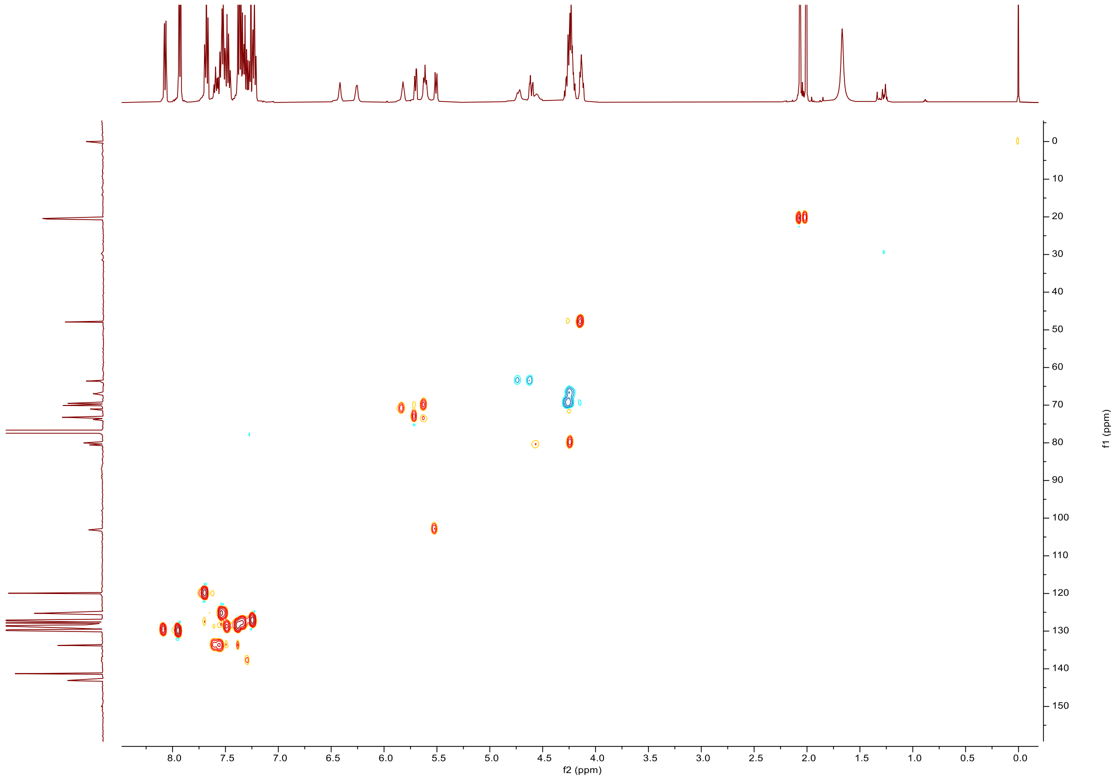

Figure S45. HSQC spectrum of compound **13β** in $CDCl_3$

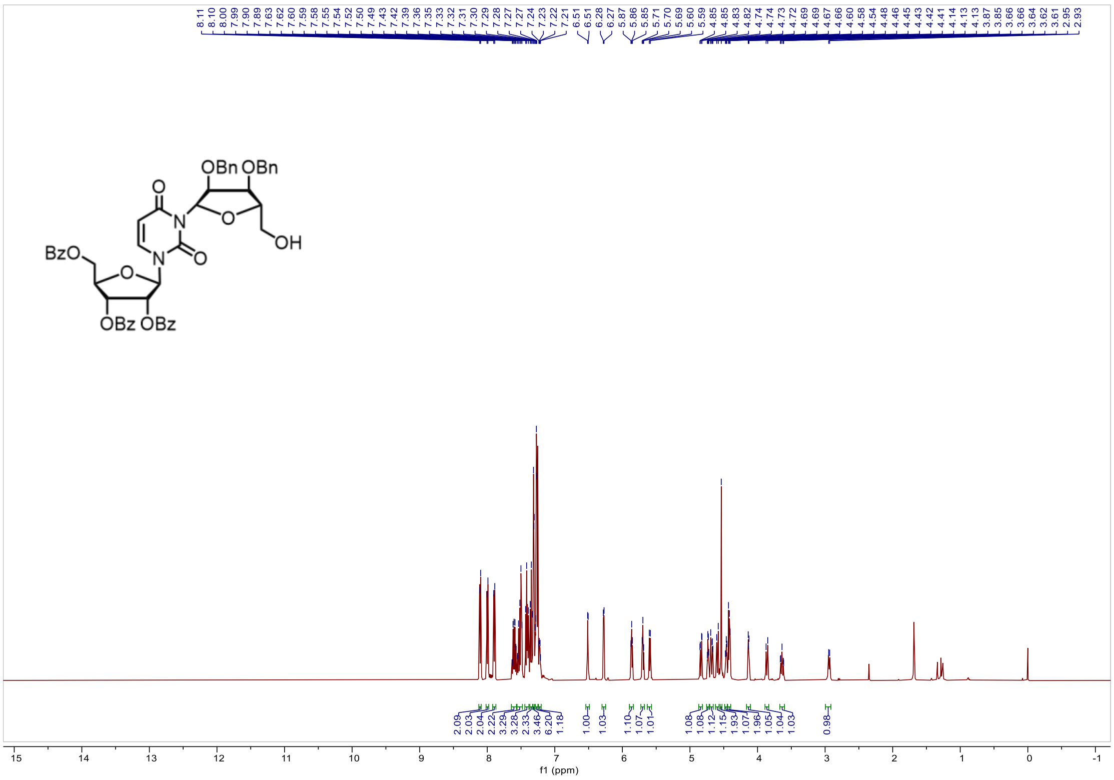

Figure S46. ^{1}H NMR spectrum of compound **4β** in $CDCl_3$

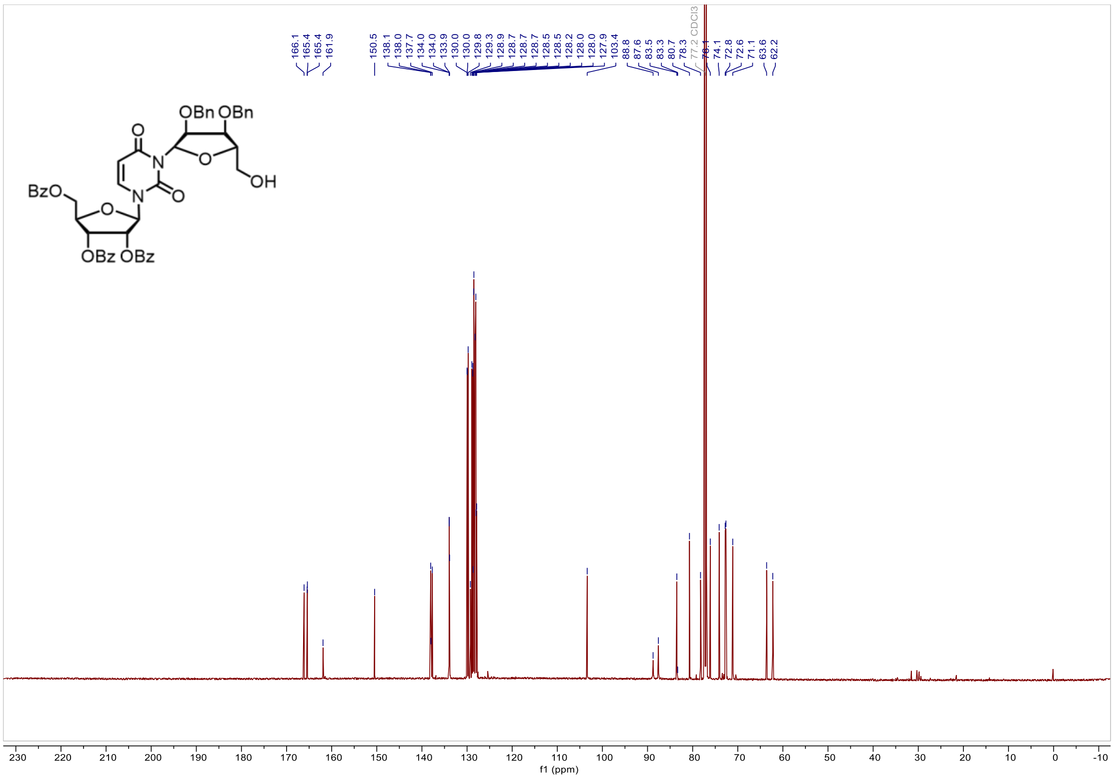

Figure S47. ^{13}C NMR spectrum of compound **4β** in $CDCl_3$

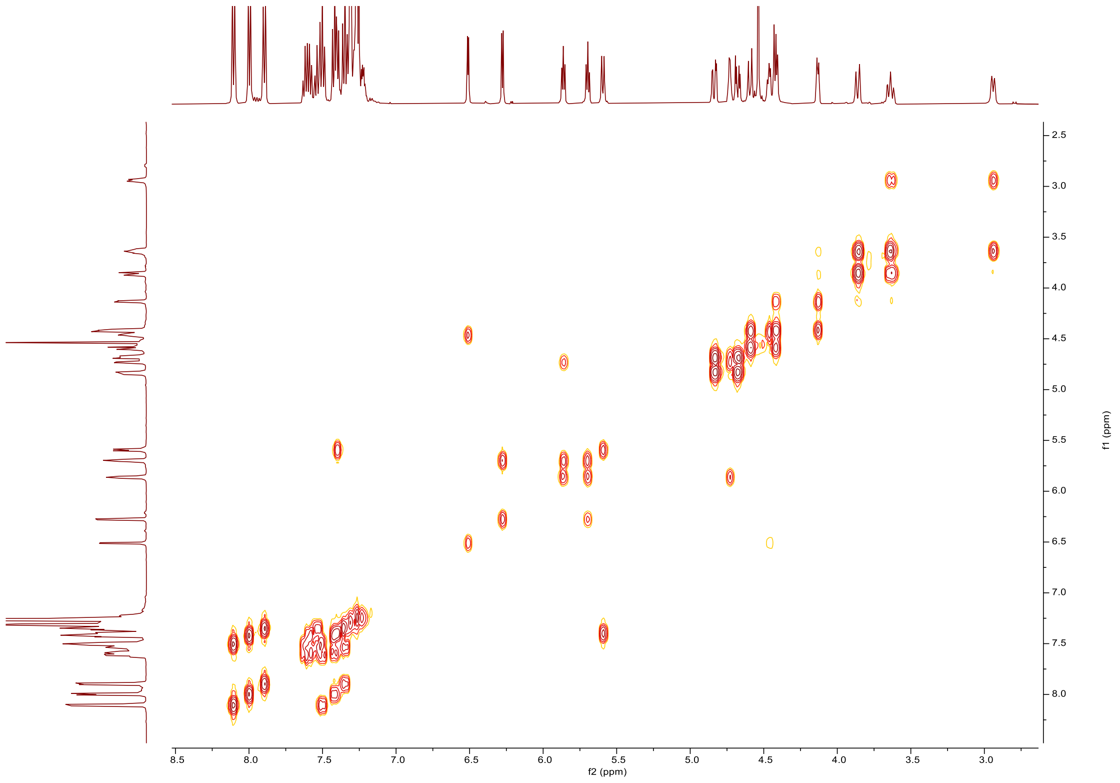

Figure S48. ^{1}H-^{1}H COSY of compound **4β** in $CDCl_3$

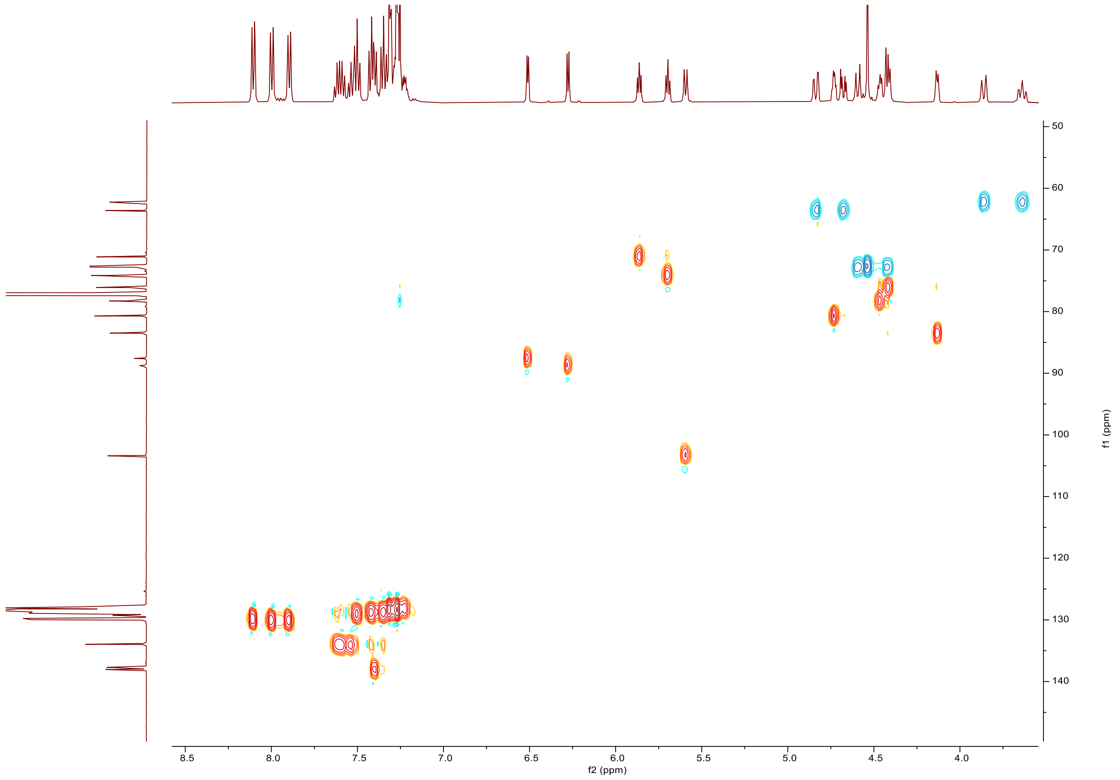

Figure S49. HSQC spectrum of compound **4β** in $CDCl_3$

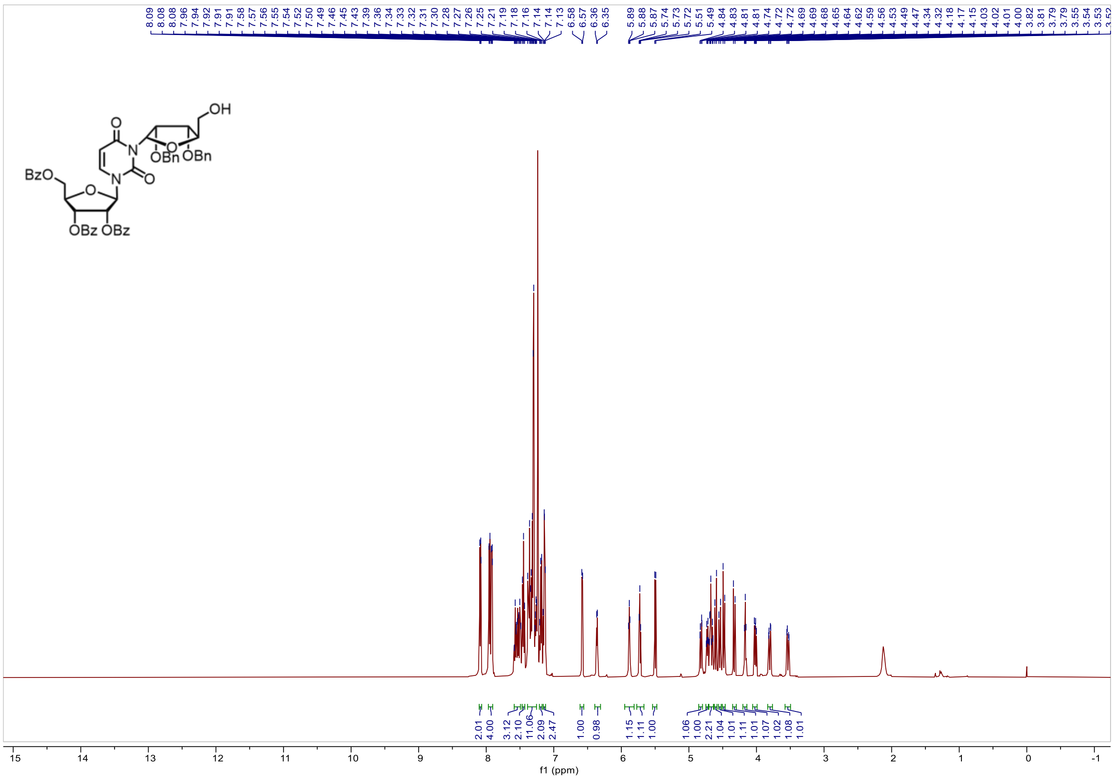

Figure S50. ^{1}H NMR spectrum of compound **4α** in $CDCl_3$

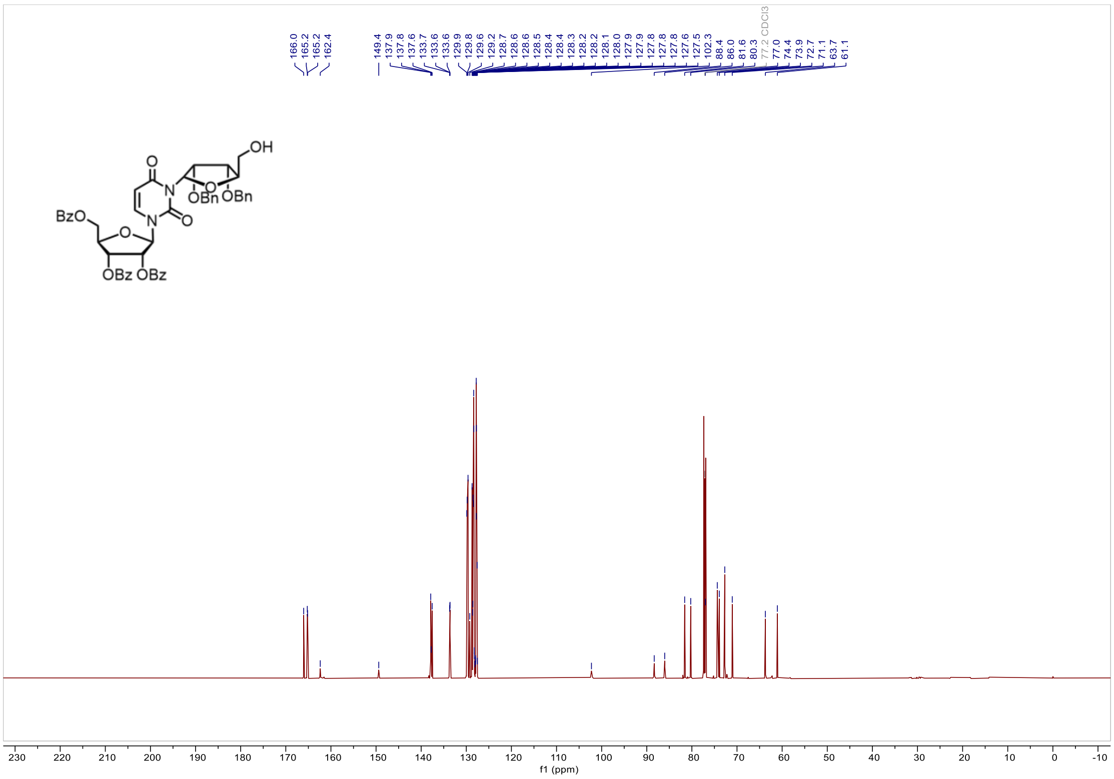

Figure S51. ^{13}C NMR spectrum of compound **4α** in $CDCl_3$

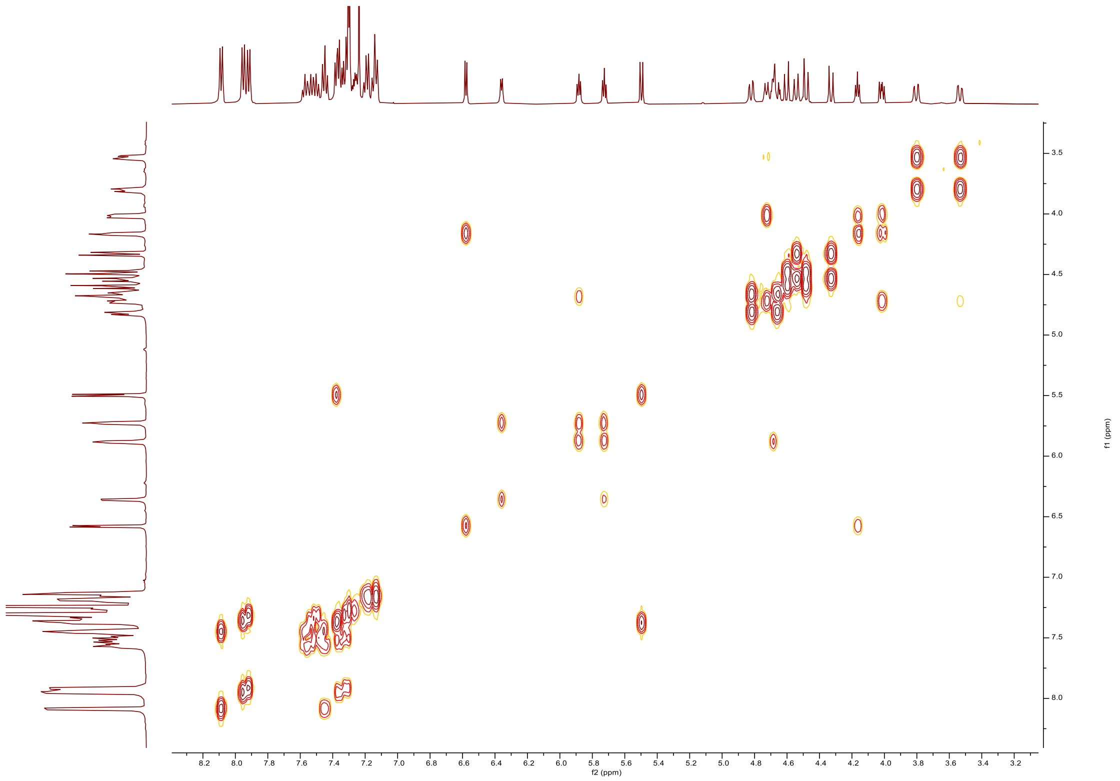

Figure S52. ^{1}H-^{1}H COSY spectrum of compound **4α** in $CDCl_3$

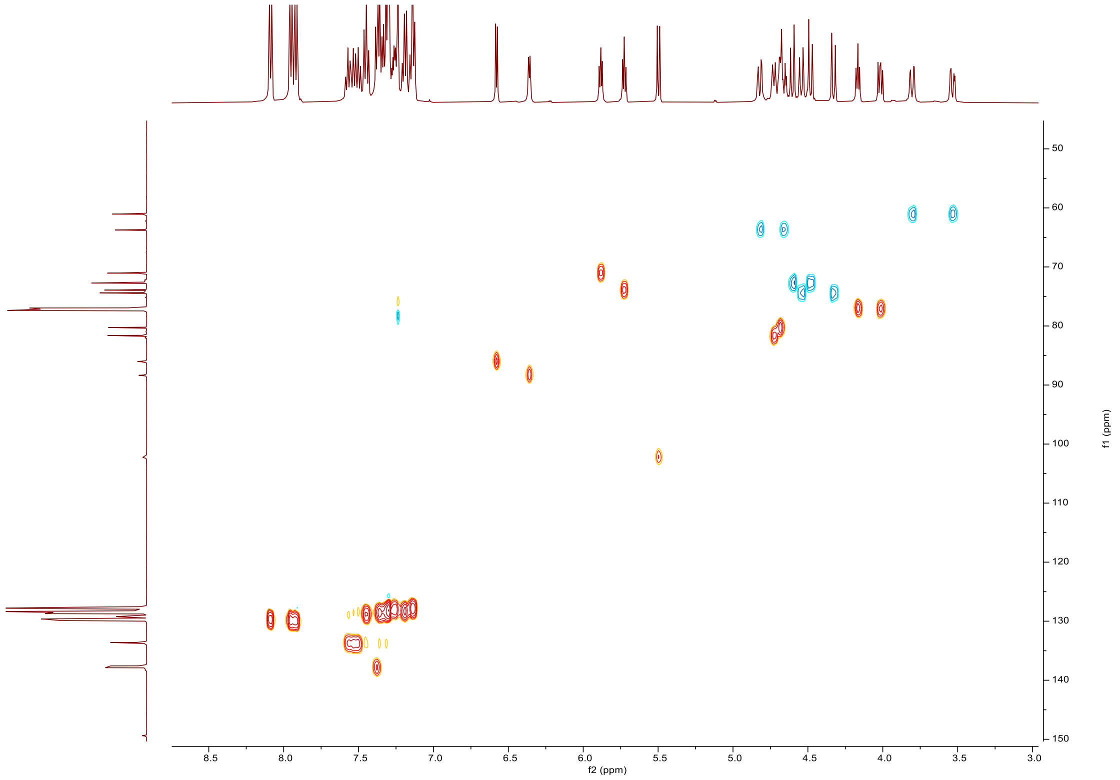

Figure S53. HSQC spectrum of compound **4α** in $CDCl_3$

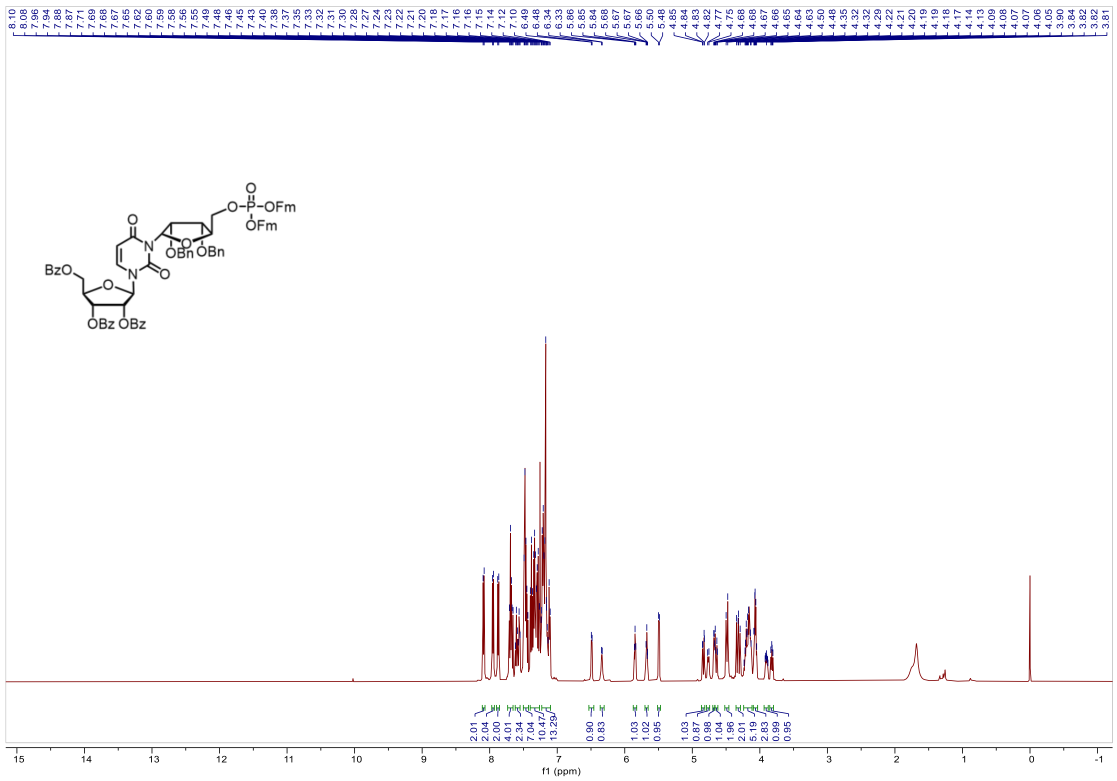

Figure S54. ^{1}H NMR spectrum of compound **6α** in $CDCl_3$

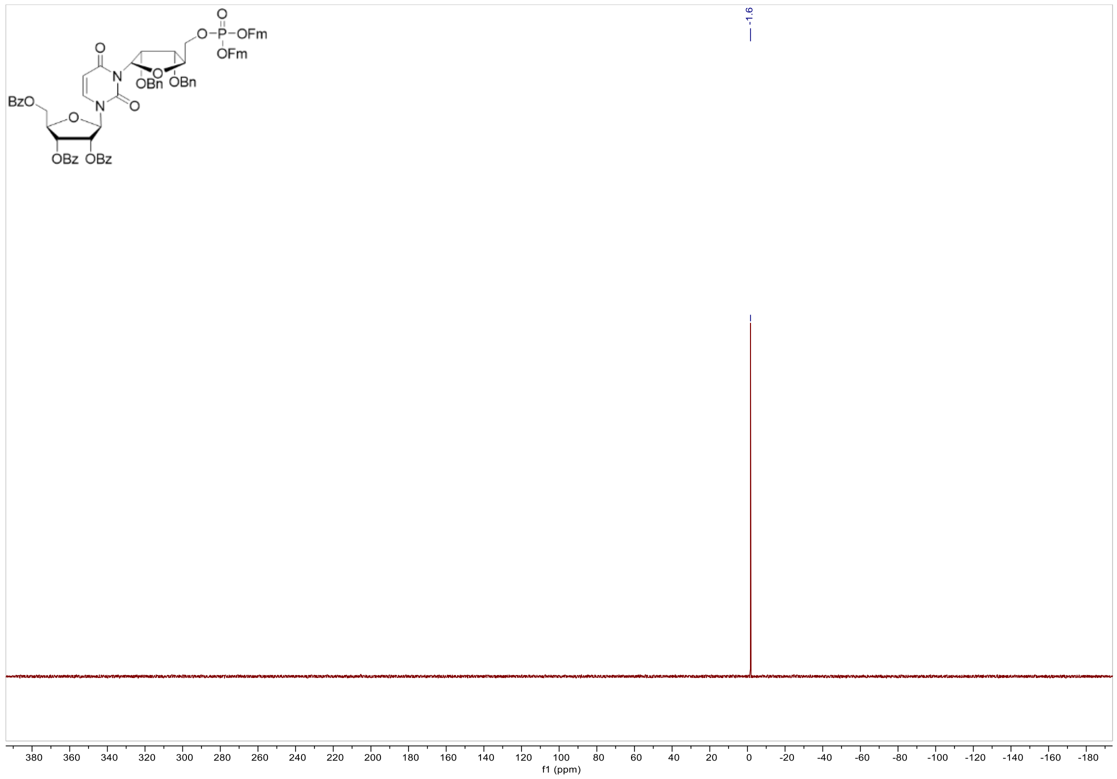

Figure S55. ^{31}P NMR spectrum of compound **6α** in $CDCl_3$

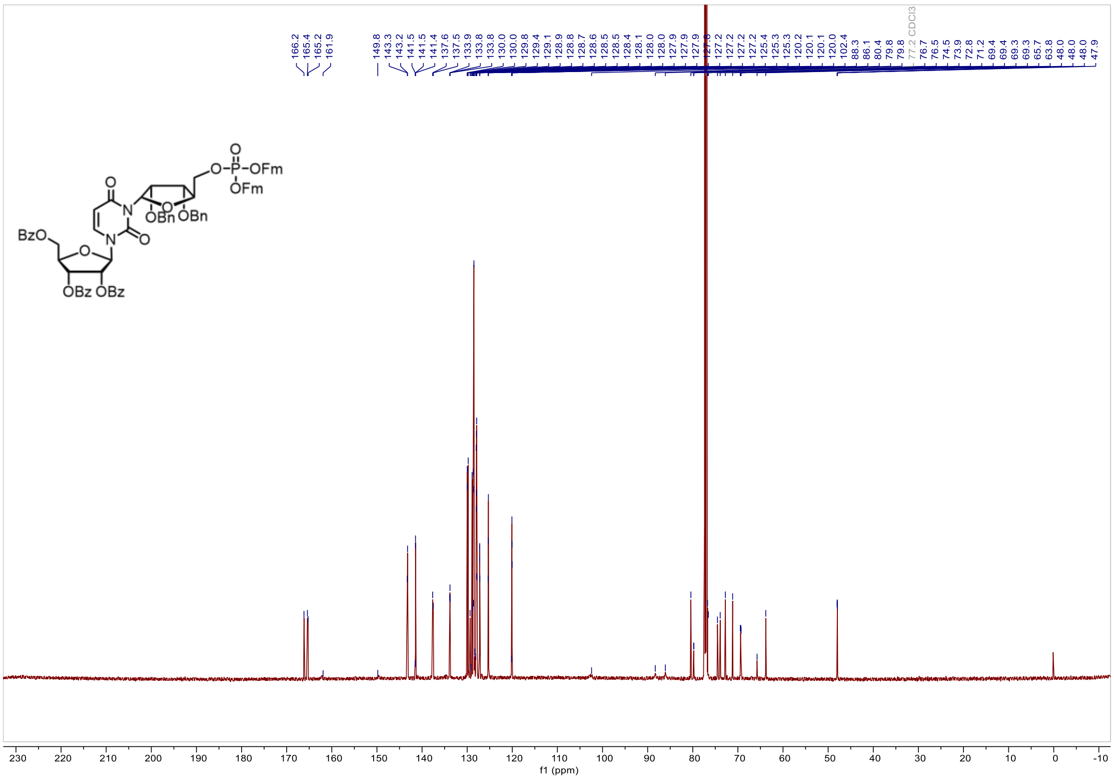

Figure S56. ^{13}C NMR spectrum of compound **6α** in $CDCl_3$

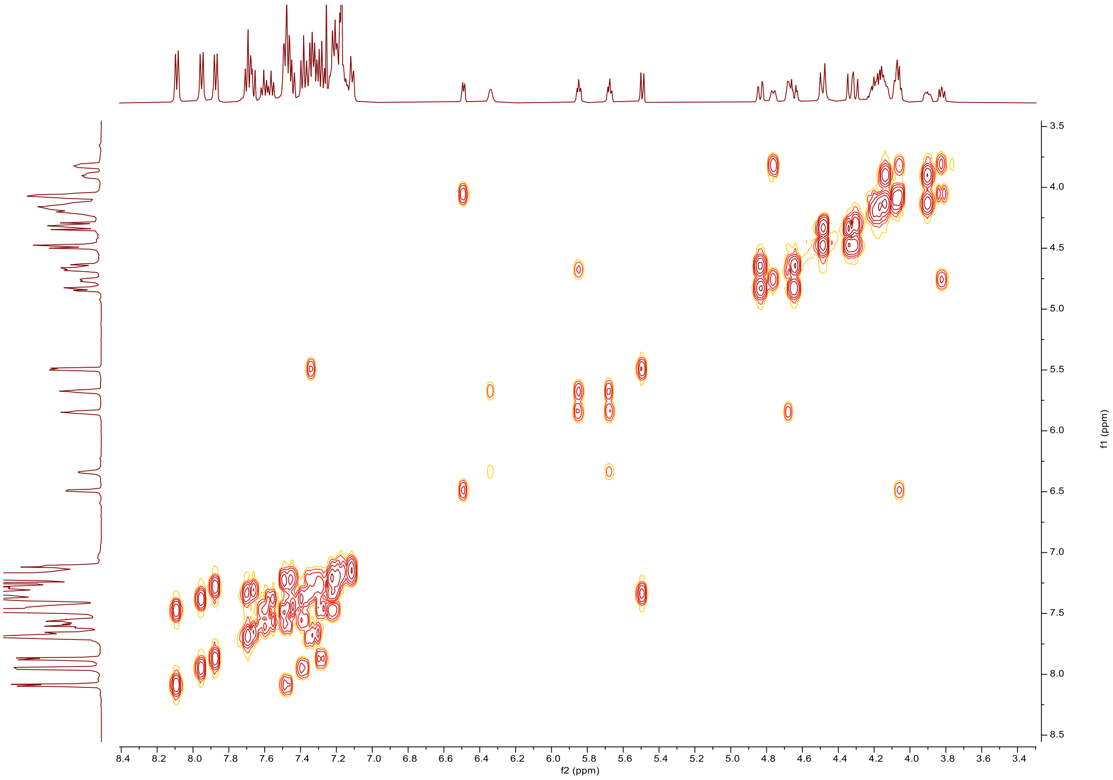

Figure S57. ^{1}H-^{1}H COSY spectrum of compound **6α** in $CDCl_3$

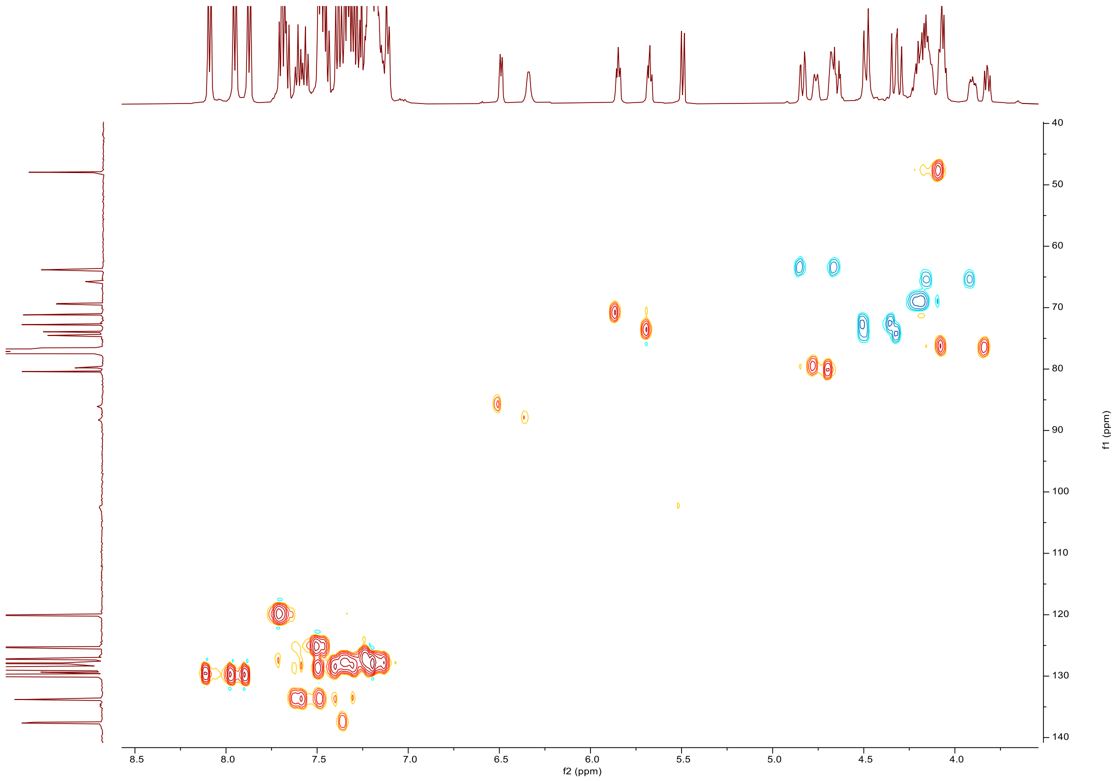

Figure S58. HSQC spectrum of compound **6α** in $CDCl_3$

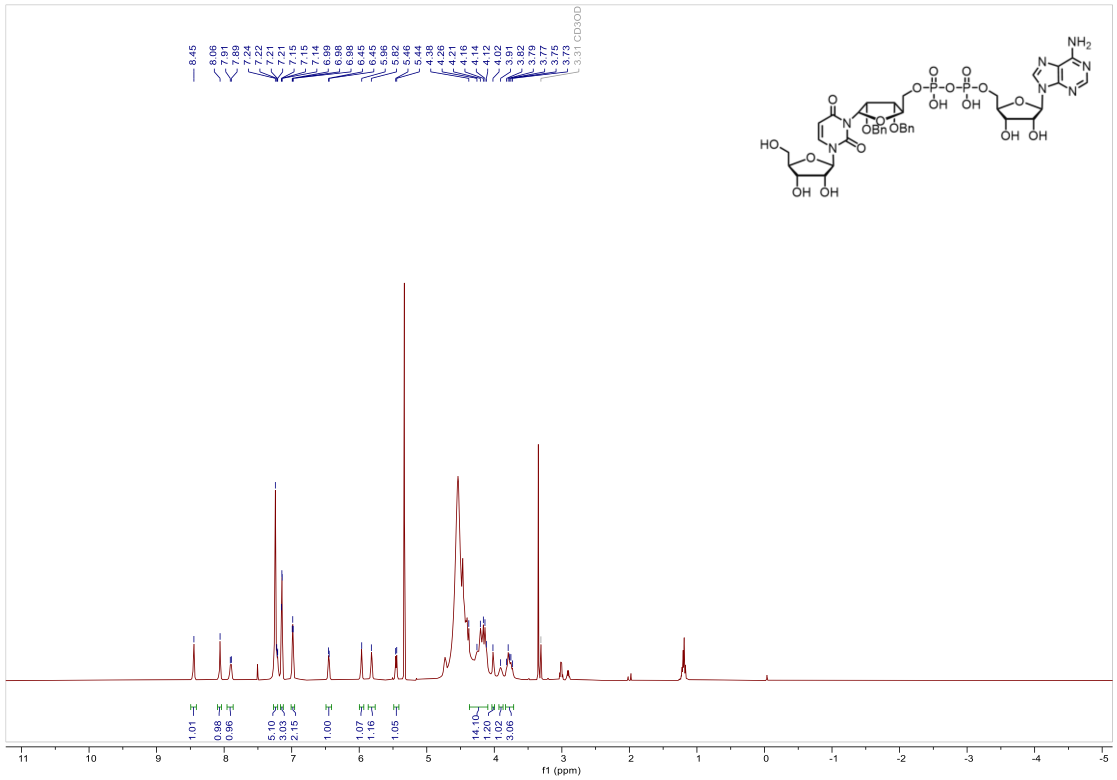

Figure S59. ^{1}H NMR spectrum of compound **8α** in $CDCl_3$

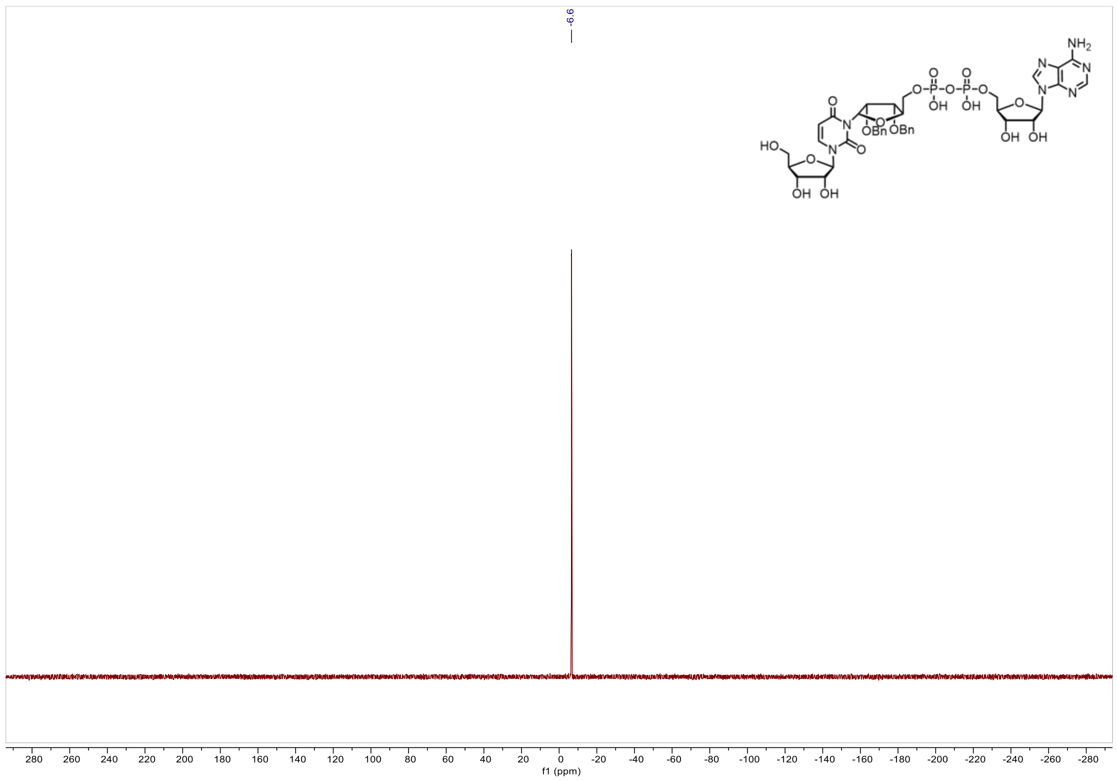

Figure S60. ^{31}P NMR spectrum of compound **8α** in $CDCl_3$

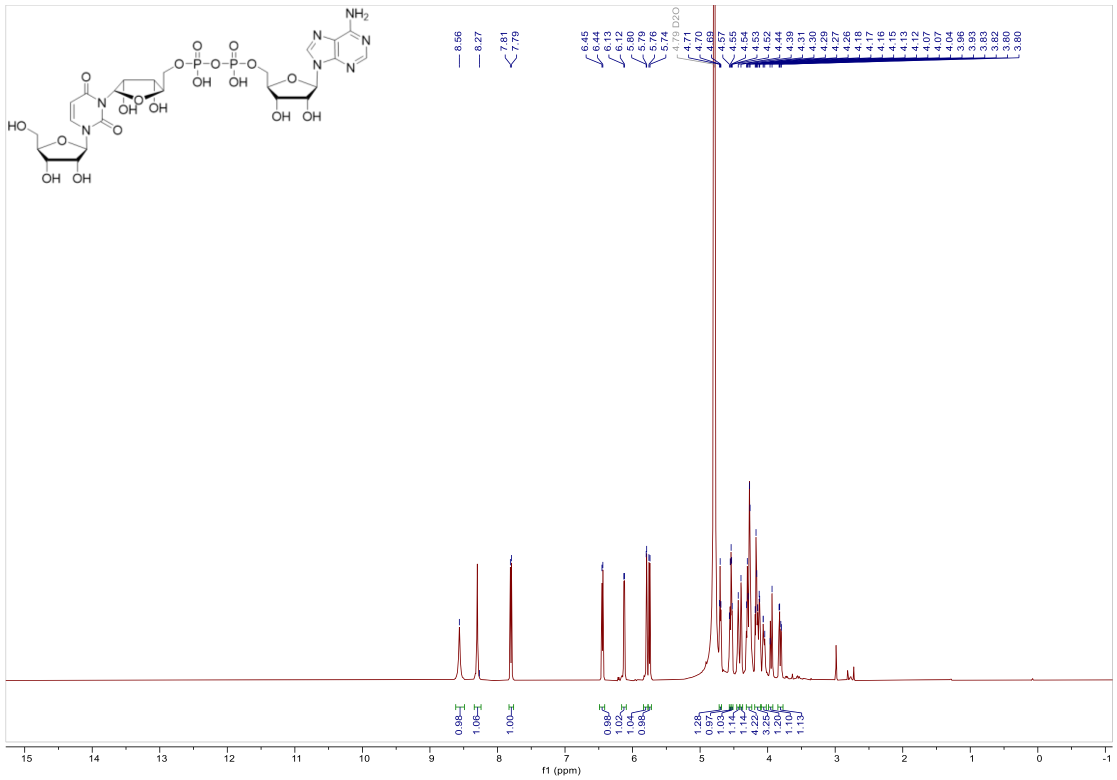

Figure S61. ^{1}H NMR spectrum of compound **U-α-ADPr** in $CDCl_3$

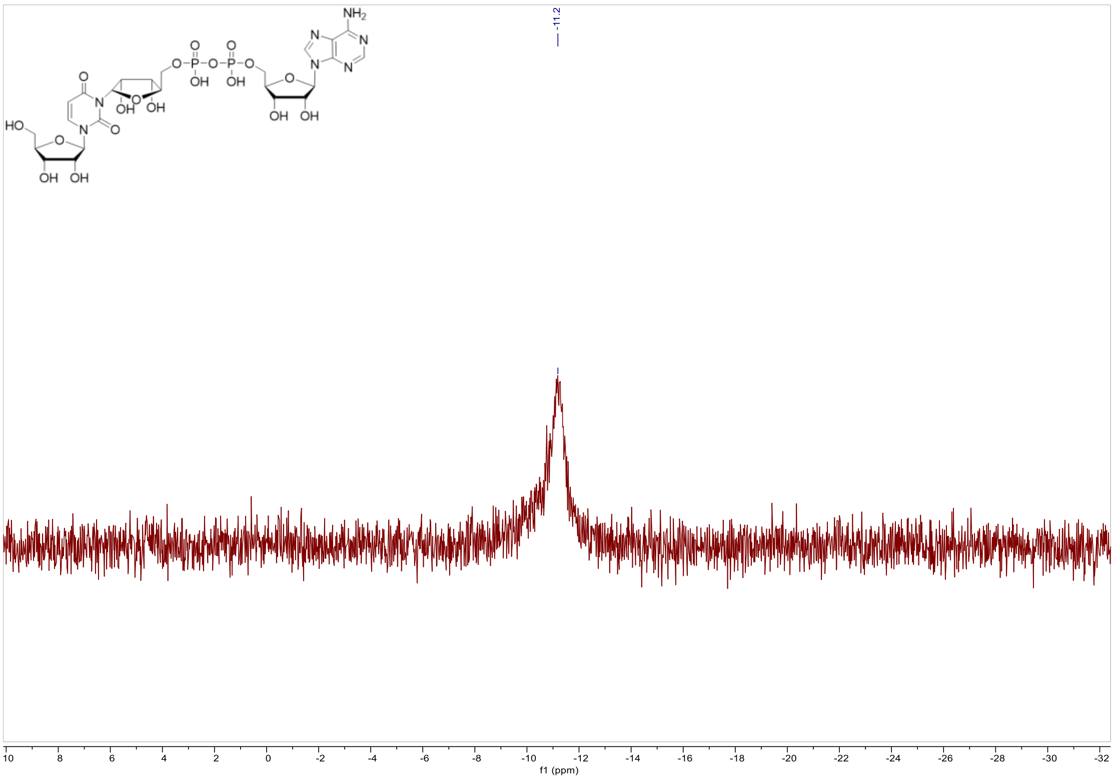

Figure S62. ^{31}P NMR spectrum of compound **U-α-ADPr** in $CDCl_3$

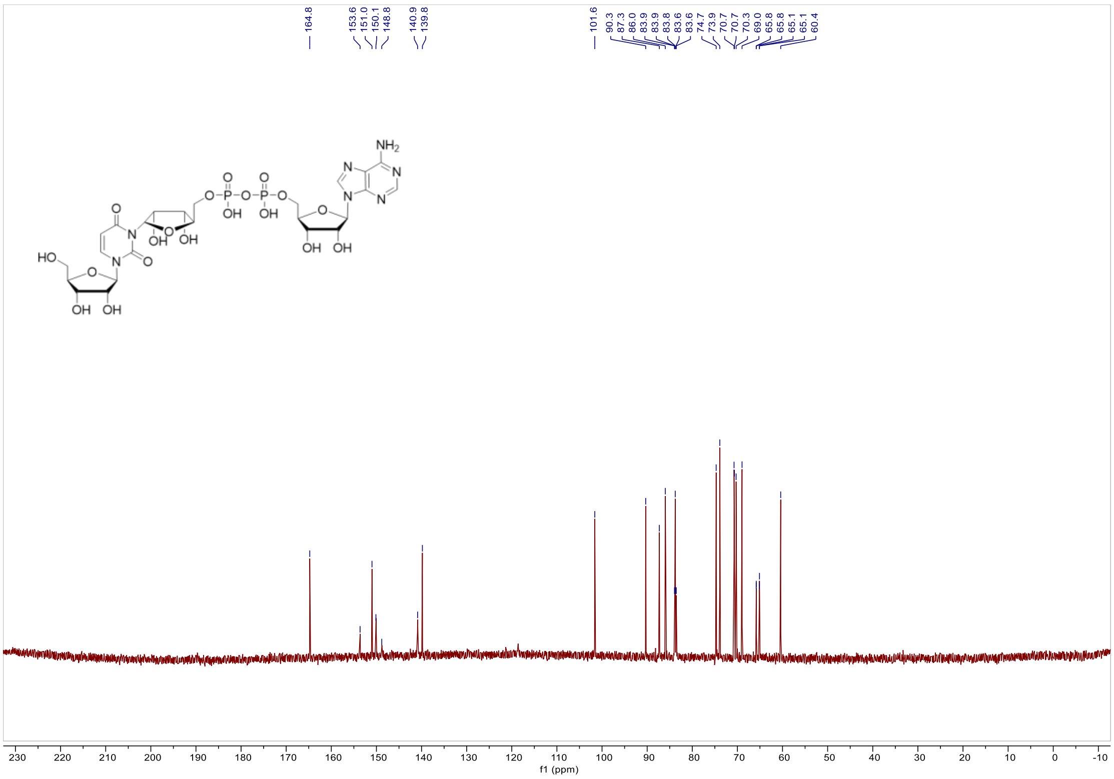

Figure S63. ^{13}C NMR spectrum of compound **U-α-ADPr** in $CDCl_3$

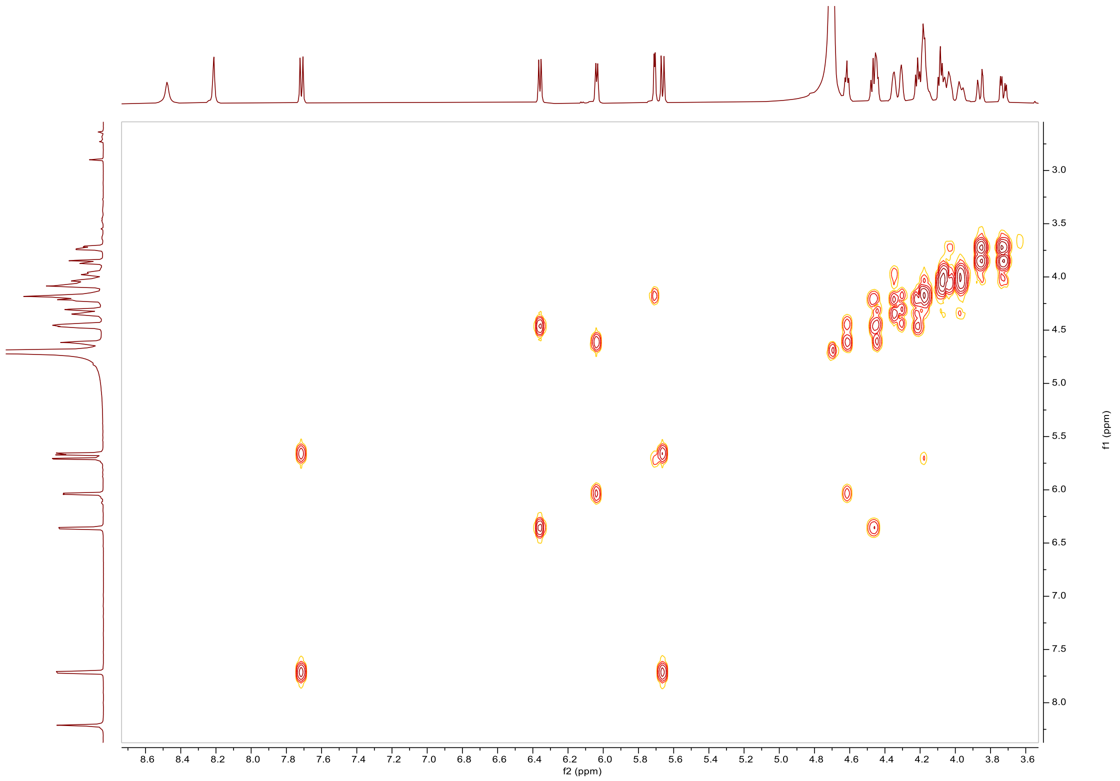

Figure S64. ^{1}H-^{1}H COSY spectrum of compound **U-α-ADPr** in $CDCl_3$

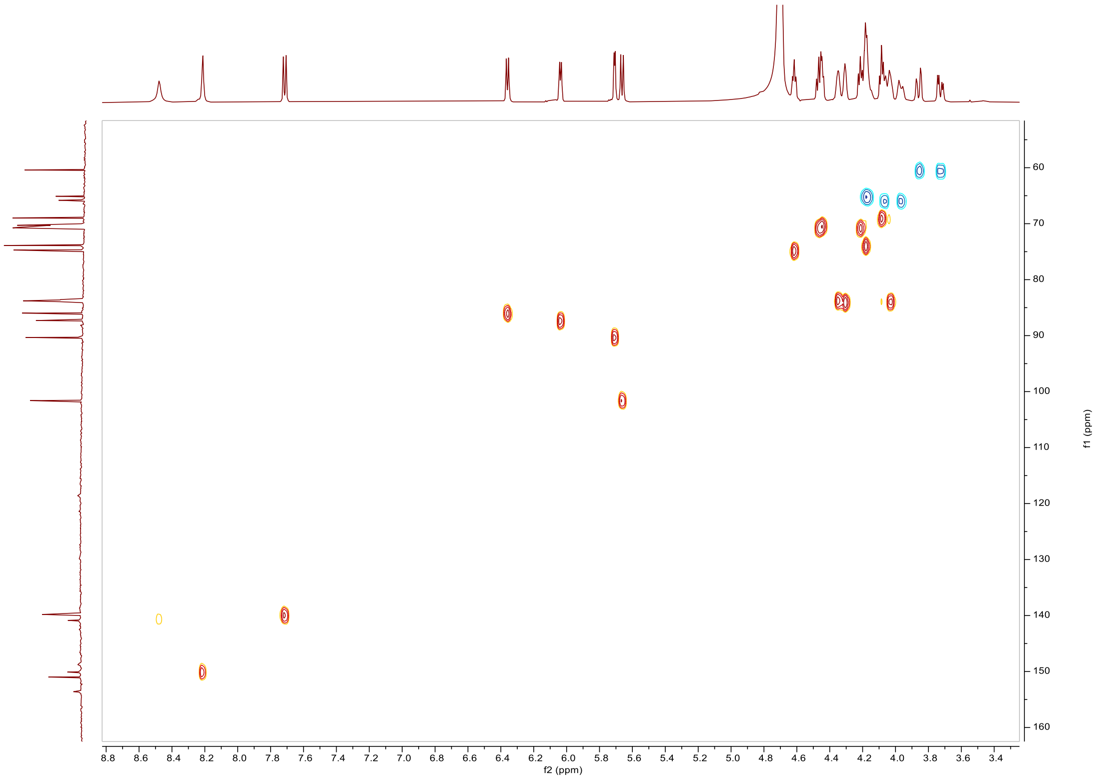

Figure S65. HSQC spectrum of compound **U-α-ADPr** in $CDCl_3$

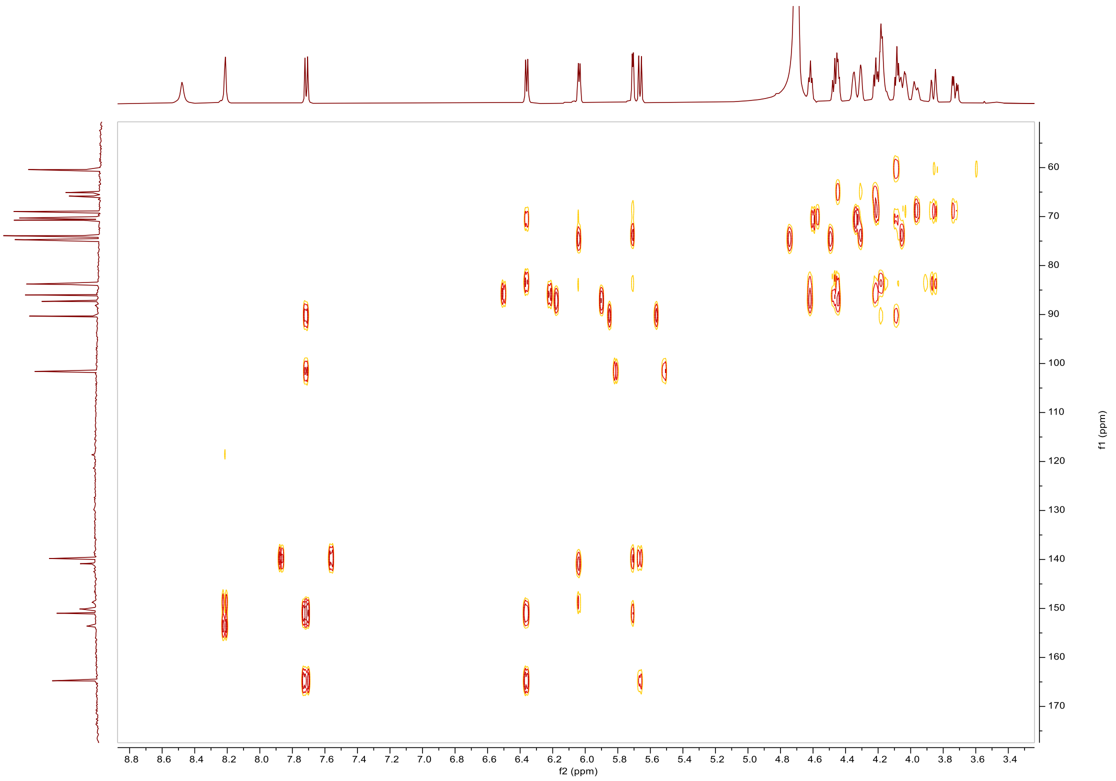

Figure S66. HMBC spectrum of compound **U-α-ADPr** in $CDCl_3$

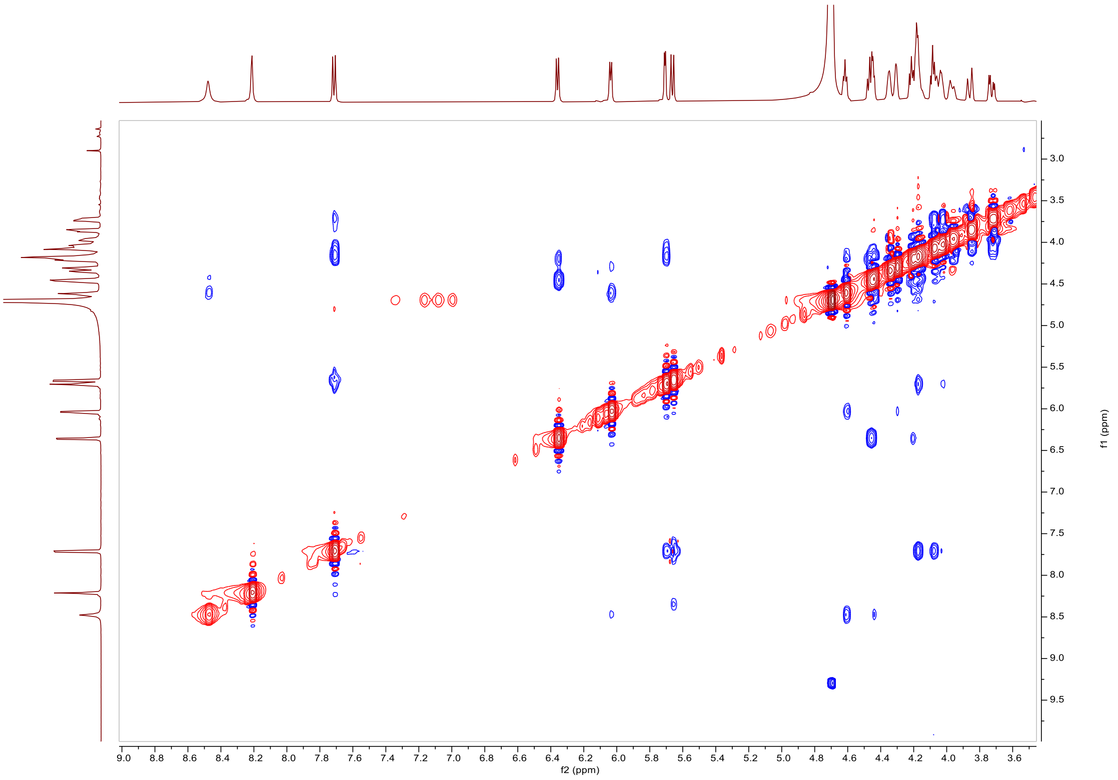

Figure S67. NOESY spectrum of compound **U-α-ADPr** in $CDCl_3$

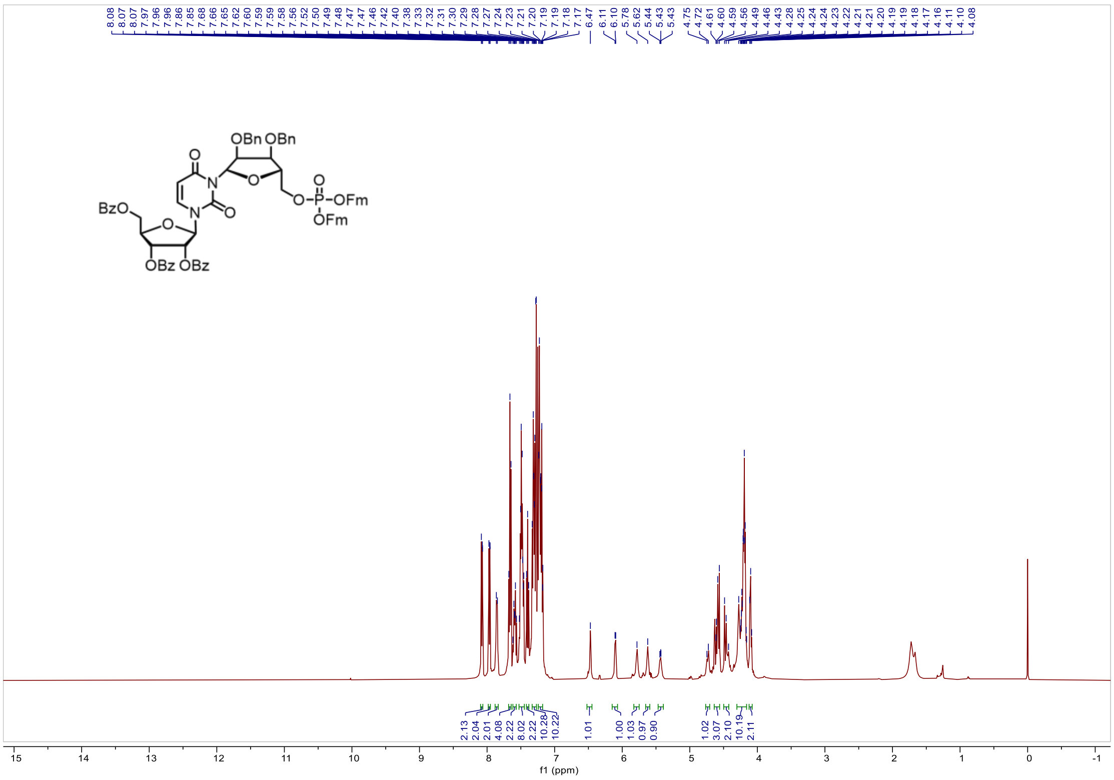

Figure S68. ^{1}H NMR spectrum of compound **6β** in $CDCl_3$

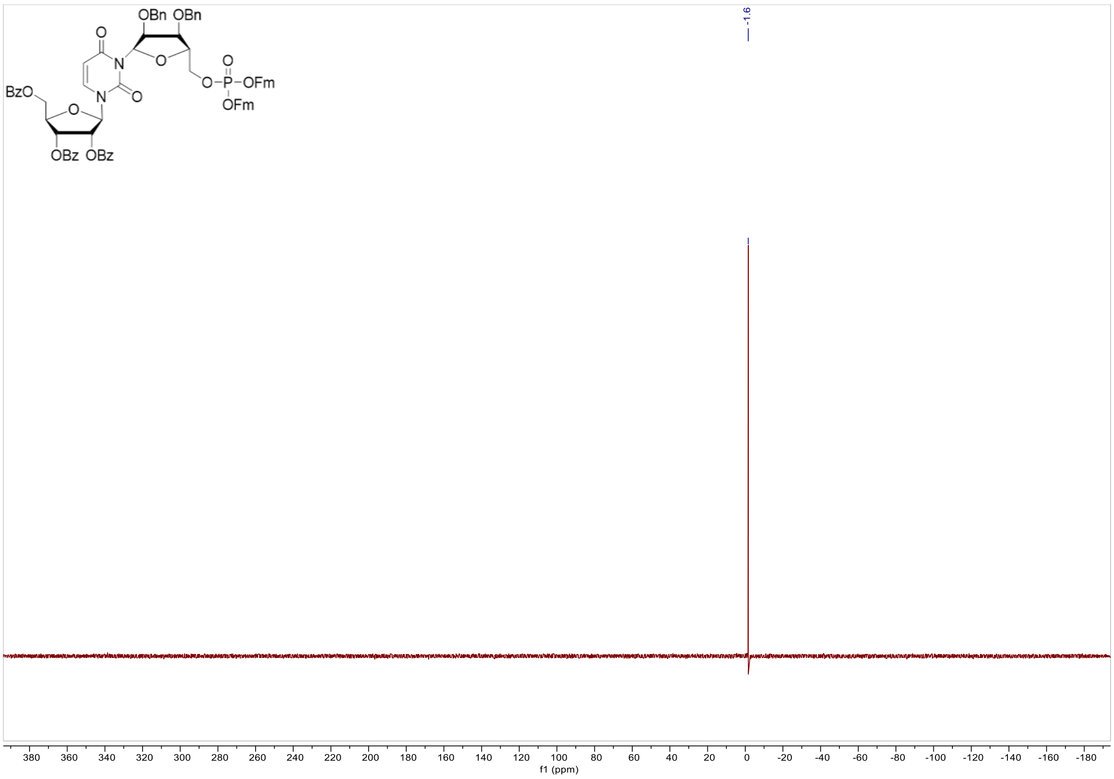

Figure S69. ^{31}P NMR spectrum of compound **6β** in $CDCl_3$

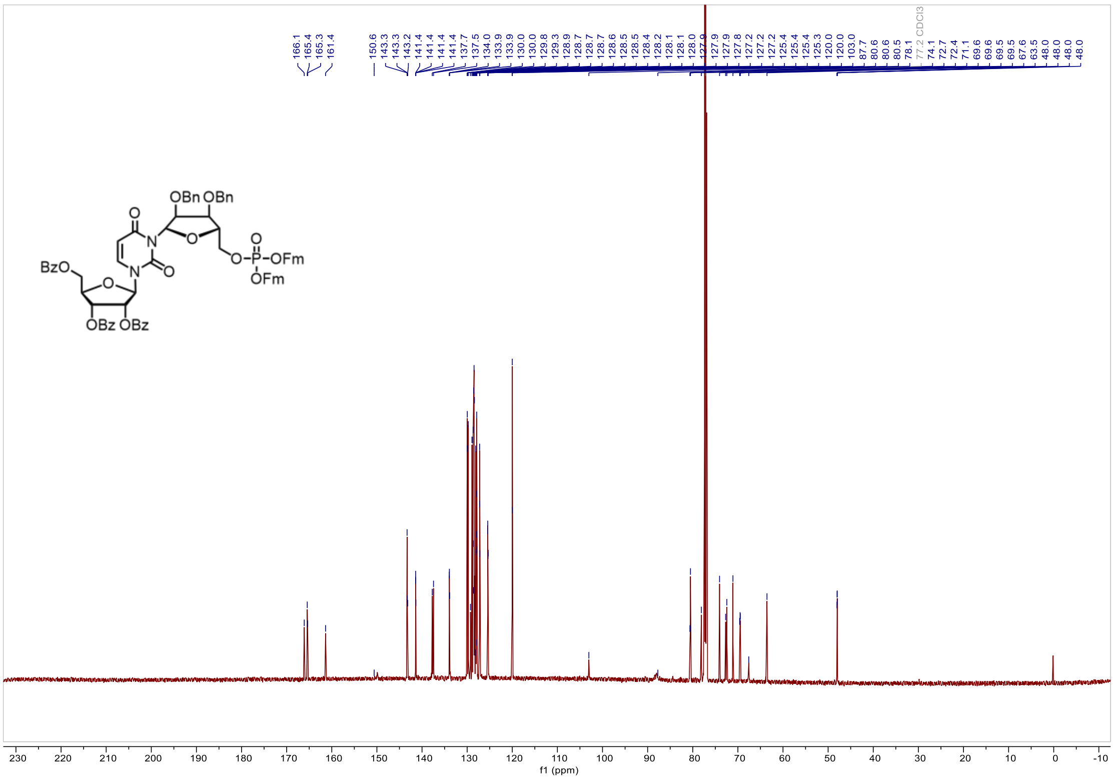

Figure S70. ^{13}C NMR spectrum of compound **6β** in $CDCl_3$

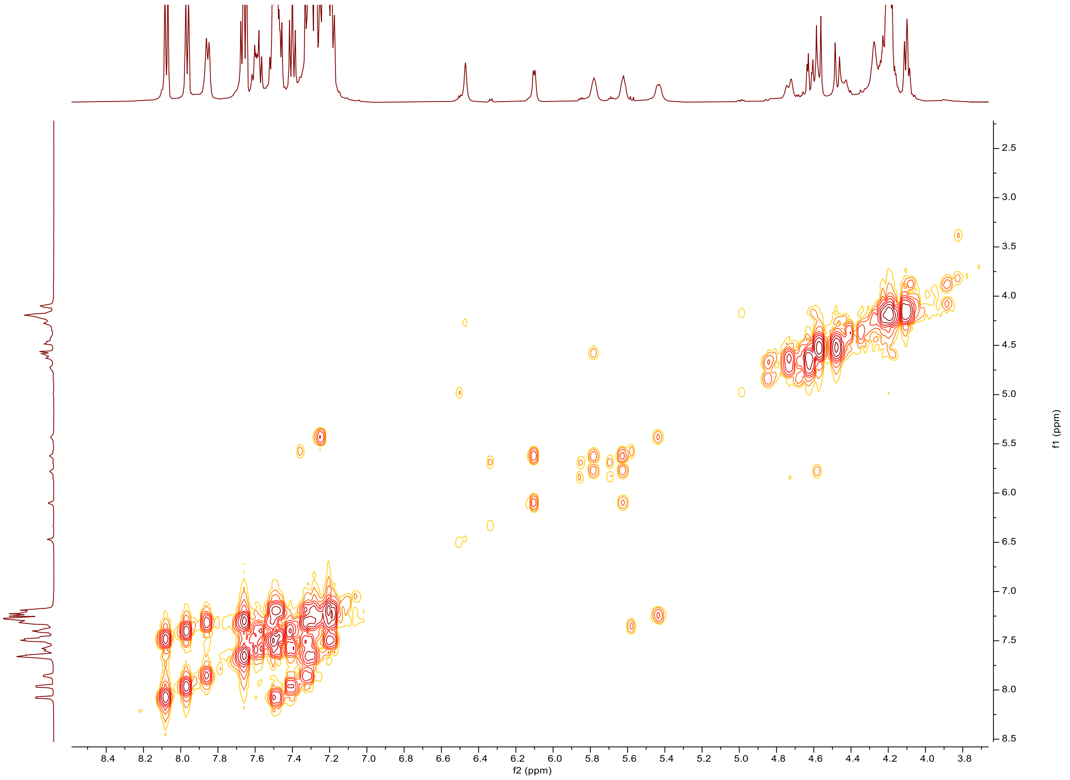

Figure S71. ^{1}H-^{1}H COSY spectrum of compound **6β** in $CDCl_3$

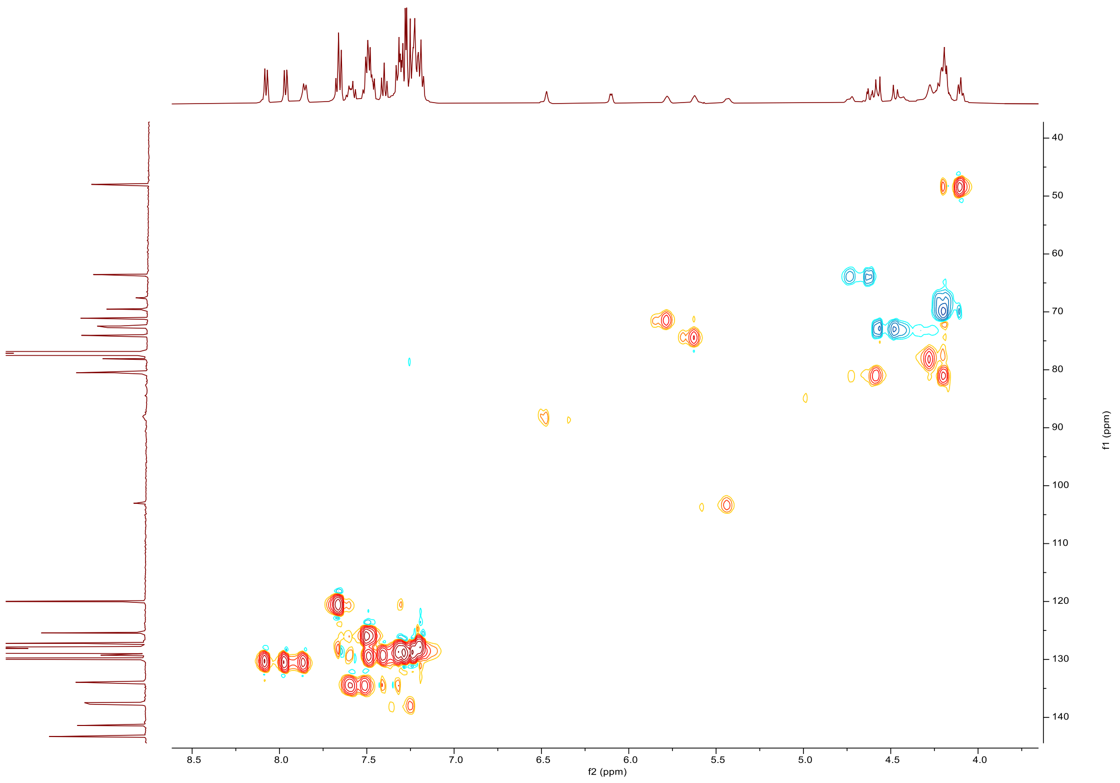

Figure S72. HSQC spectrum of compound **6β** in $CDCl_3$

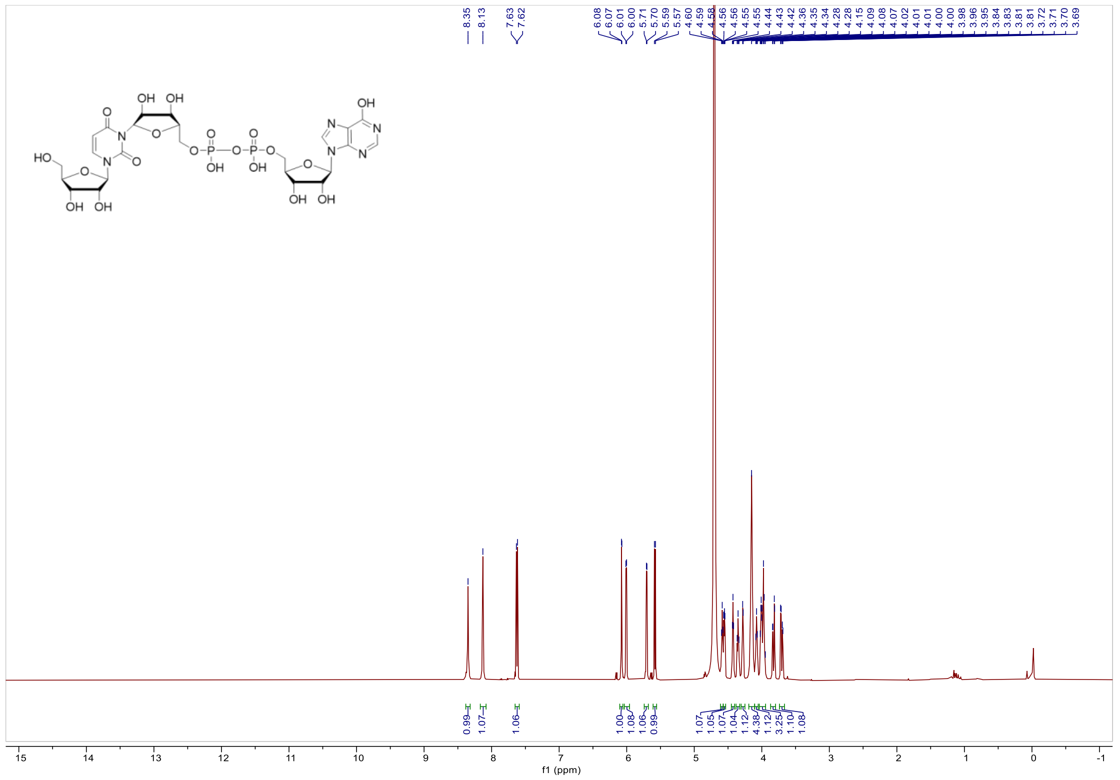

Figure S73. ^{1}H NMR spectrum of compound **U-β-ADPr** in $CDCl_3$

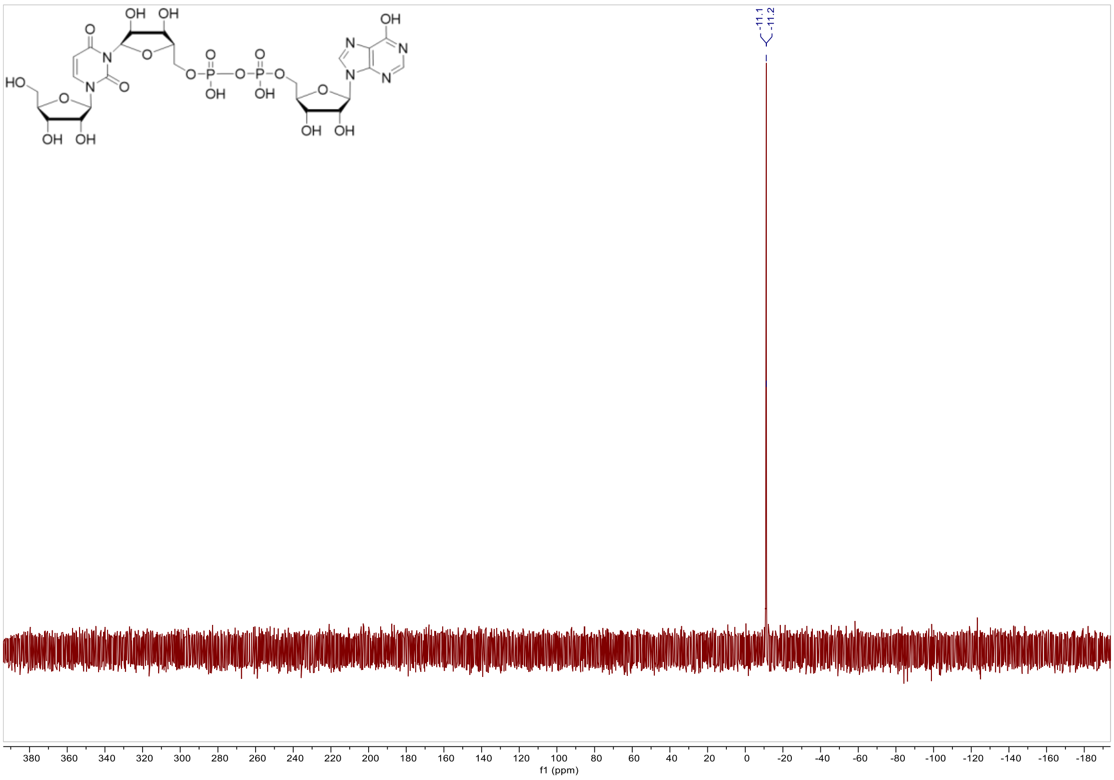

Figure S74. ^{31}P NMR spectrum of compound **U-β-ADPr** in $CDCl_3$

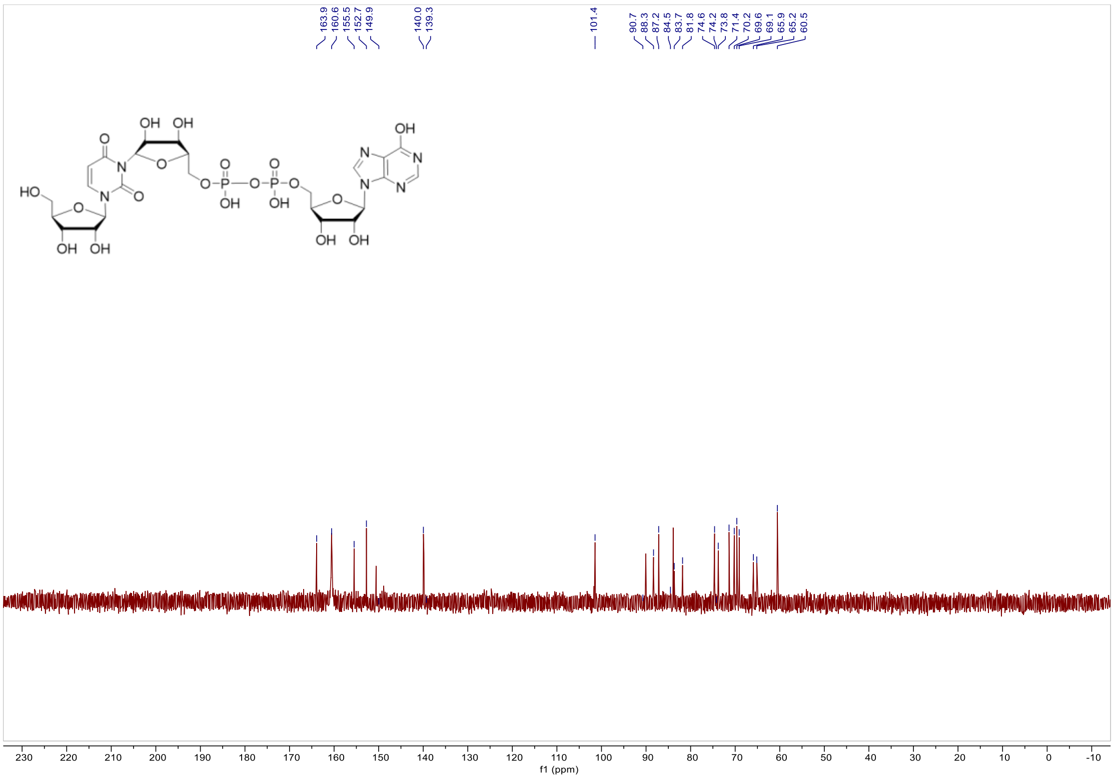

Figure S75. ^{13}C NMR spectrum of compound **U-β-ADPr** in $CDCl_3$

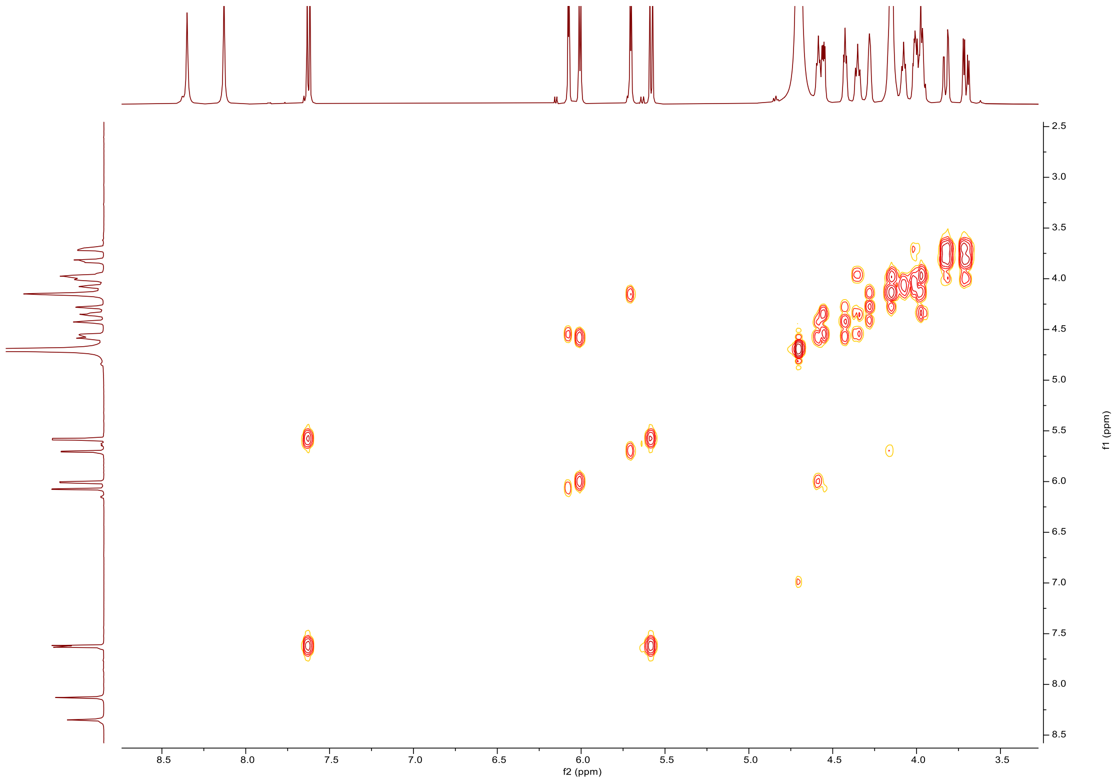

Figure S76. ^{1}H-^{1}H COSY spectrum of compound **U-β-ADPr** in $CDCl_3$

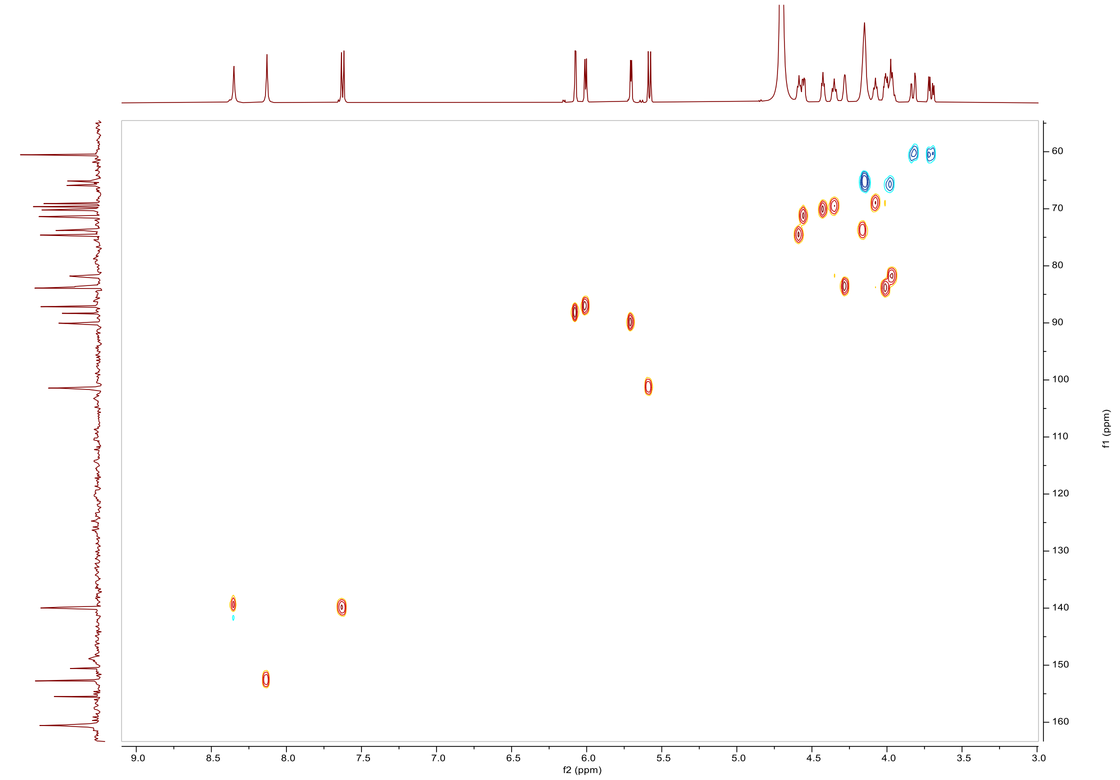

Figure S77. HSQC spectrum of compound **U-β-ADPr** in $CDCl_3$

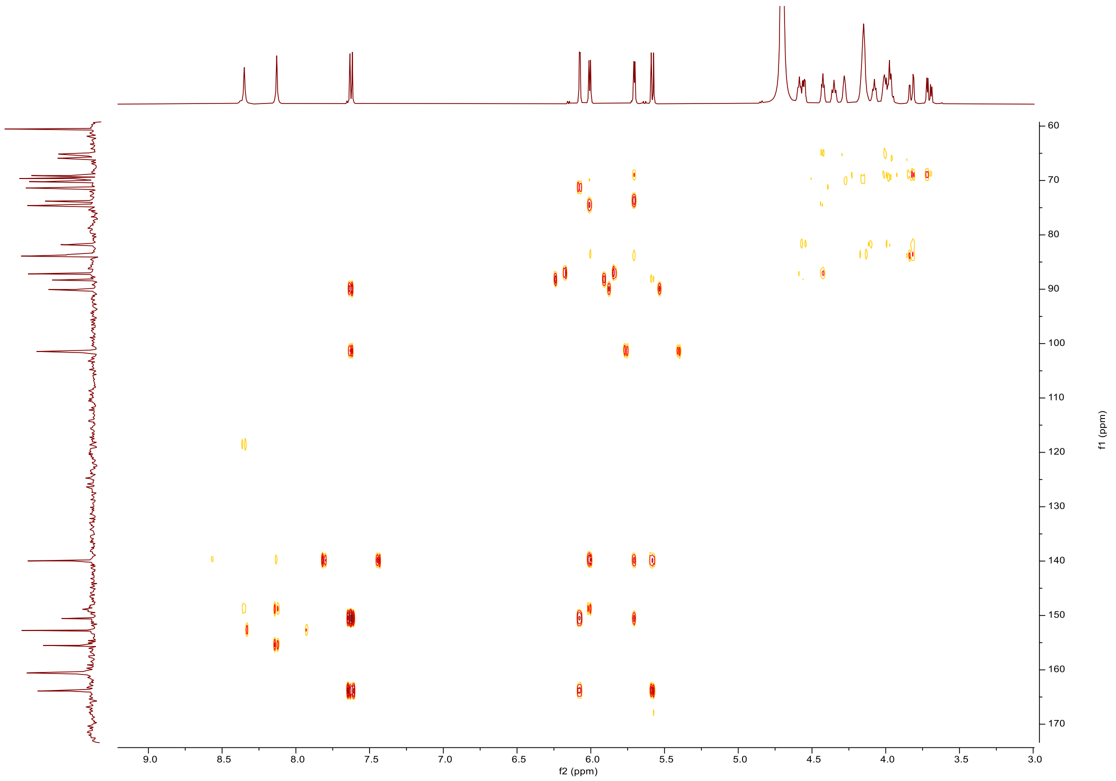

Figure S78. HMBC spectrum of compound **U-β-ADPr** in $CDCl_3$

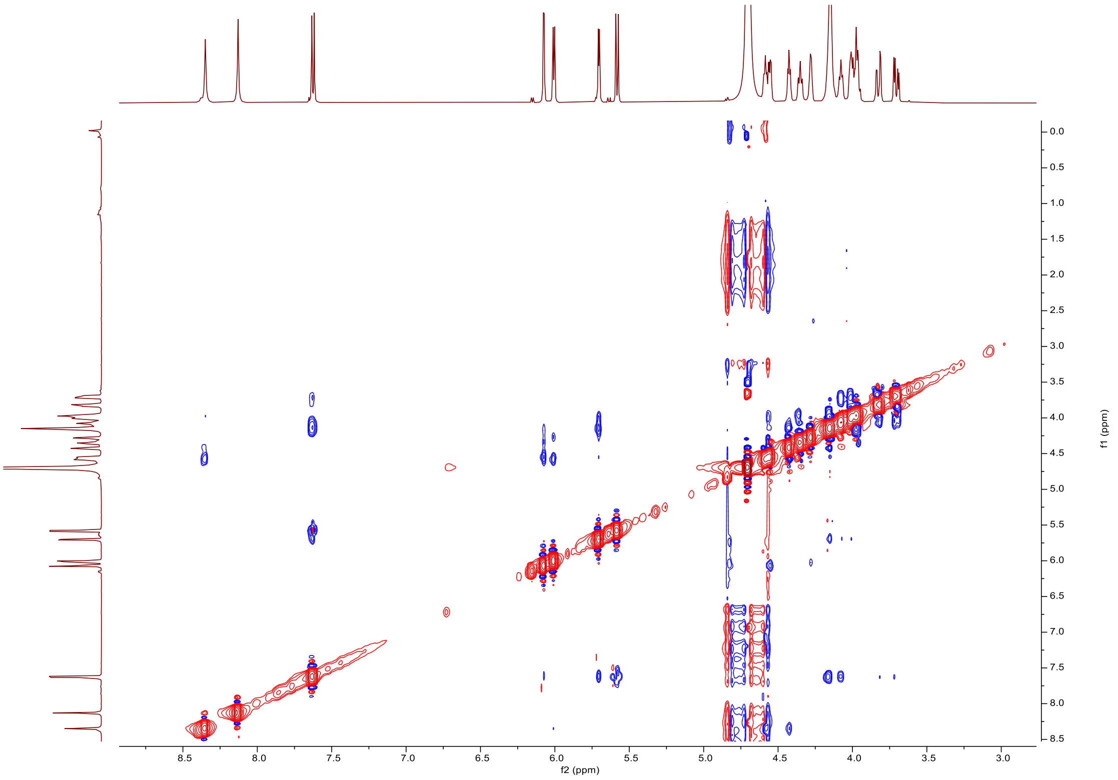

Figure S79. NOESY spectrum of compound **U-β-ADPr** in $CDCl_3$

Reference

1. Sniady, A., Bedore, M.W. and Jamison, T.F. (2011) One-Flow, Multistep Synthesis of Nucleosides by Bronsted Acid-Catalyzed Glycosylation. *Angewandte Chemie-International Edition*, **50**, 2155-2158.

2. Kistemaker, H.A.V., van Noort, G.J.V., Overkleeft, H.S., van der Marel, G.A. and Filippov, D.V. (2013) Stereoselective Ribosylation of Amino Acids. *Organic Letters*, **15**, 2306-2309.

3. Watanabe, Y., Nakamura, T. and Mitsumoto, H. (1997) Protection of phosphate with the 9-fluorenylmethyl group. Synthesis of unsaturated-acyl phosphatidylinositol 4,5-bisphosphate. *Tetrahedron Letters*, **38**, 7407-7410.

4. van Noort, G.J.V., Overkleeft, H.S., van der Marel, G.A. and Filippov, D.V. (2010) Synthesis of Nucleotidylated Poliovirus VPg Proteins. *Journal of Organic Chemistry*, **75**, 5733-5736.
